# Supplementary material for: Understanding VPAC receptor family peptide binding and selectivity
Source: Nat Commun. 2022 Nov 16;13:7013. doi: 10.1038/s41467-022-34629-3 (PMC9668914; doi:10.1038/s41467-022-34629-3)

Supplementary Information
Understanding VPAC receptor family peptide binding and selectivity

Piper et al.

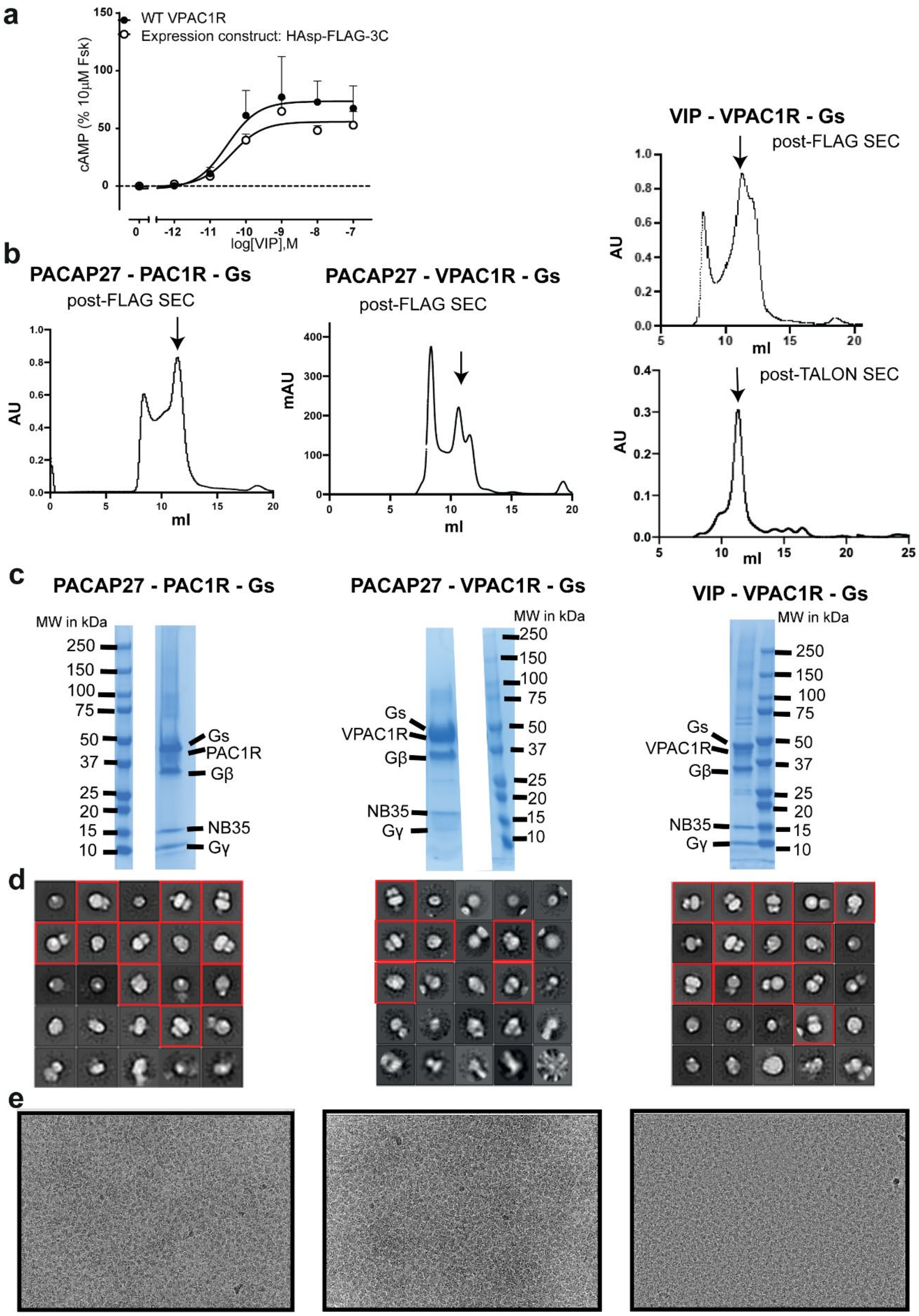
a
WT VPAC1R
Expression construct: HAsp-FLAG-3C
cAMP (% 10μM Fsk)
log[VIP],M
b
PACAP27 - PAC1R - Gs
post-FLAG SEC
PACAP27 - VPAC1R - Gs
post-FLAG SEC
VIP - VPAC1R - Gs
post-FLAG SEC
post-TALON SEC
AU
mAU
ml
c
PACAP27 - PAC1R - Gs
PACAP27 - VPAC1R - Gs
VIP - VPAC1R - Gs
MW in kDa
Gs
PAC1R
VPAC1R
Gβ
NB35
Gγ
d
e

Supplementary Figure 1. Complex purification and quality control. a: cAMP accumulation data for the VPAC1R expression construct used in this study (white circles), in comparison with the wild-type construct (black circles), in response to VIP. Data are presented as mean+SEM of 3 independent experiments. HAsp: HA signal peptide. FLAG, HIS: Purification tags. 3C: 3C protease cleavage site. b: Size-exclusion chromatography (SEC) trace of the PAC1R-PACAP27-Gs, VPAC1R-PACAP27-Gs and VPAC1R-VIP-Gs complex purifications, with the complex peak eluting at ~11-13 ml (indicated by arrows). For the VPAC1R-VIP sample, two size-exclusion chromatography steps were conducted, after FLAG resin elution (1.) and after Talon resin elution (2.). c: Coomassie-stained SDS-PAGE of the complex sample used for cryo-EM imaging, showing bands of all complex components (protein marker molecular weight indicated in kilodalton (kDa)): Dominant-negative Gαs subunit (Gs), receptor VPAC1R (right and middle) or PAC1R (left), Gβ subunit, Gγ subunit and Nanobody 35 (NB35). Uncropped Coomassie-stained SDS-PAGE are available at the end of this Supplementary Information file (below). d: Negative-stain TEM 2D classification of complex sample used for cryo-EM imaging, with 2D classes of complexes framed in red. e: Representative micrographs for each data collection/complex, at around 1 μm defocus, displayed using a lowpass filter (20 Å). The complex purification and data collection for each structure obtained in this manuscript was performed once (n=1).

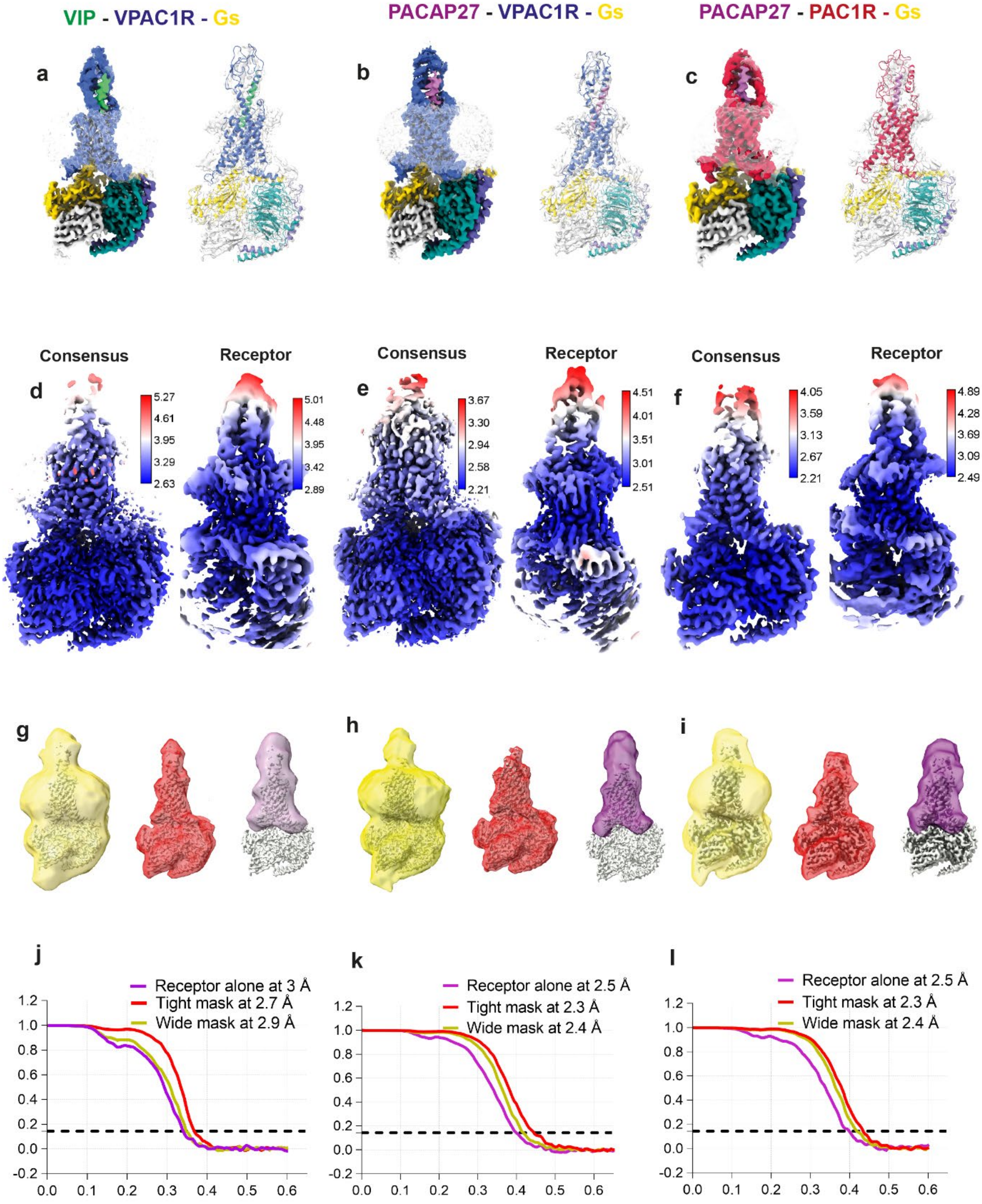

Supplementary Figure 2. Cryo-EM maps and models of active, Gs-coupled PACAP family complexes. Complexes of VPAC1R-VIP-Gs (a,d,g,j), VPAC1R-PACAP27-Gs (b,e,h,k) and PAC1R-PACAP27-Gs (c,f,I,l) are separated by columns and different subunit colouring. a-c: Consensus map and model fit of the receptor-peptide complexes with coloured map surface (left) and transparent map and ribbon model (right). Colours: VIP: spring green, PACAP27: orchid, PAC1R: red, VPAC1R: royal blue, Gs: goldenrod, Gβ: dark cyan, Gγ: slate blue, Nanobody35: white. d-f: Consensus map (left) and receptor-focused map (right) coloured by resolution (blue: high, red: low). g-i: Consensus wide mask (yellow), consensus tight mask (red) and receptor-focused mask (purple) applied during 3D refinement and/or post-

processing. j-l: Fourier-shell correlation (FSC) curves indicating the resolution determined using the gold-standard 0.143 cut-off, for the masked, post-processed maps, coloured according to mask applied (see g-i). Purple: receptor-alone focus; red: tight mask (without micelle and α-helical domain); yellow: wide mask.

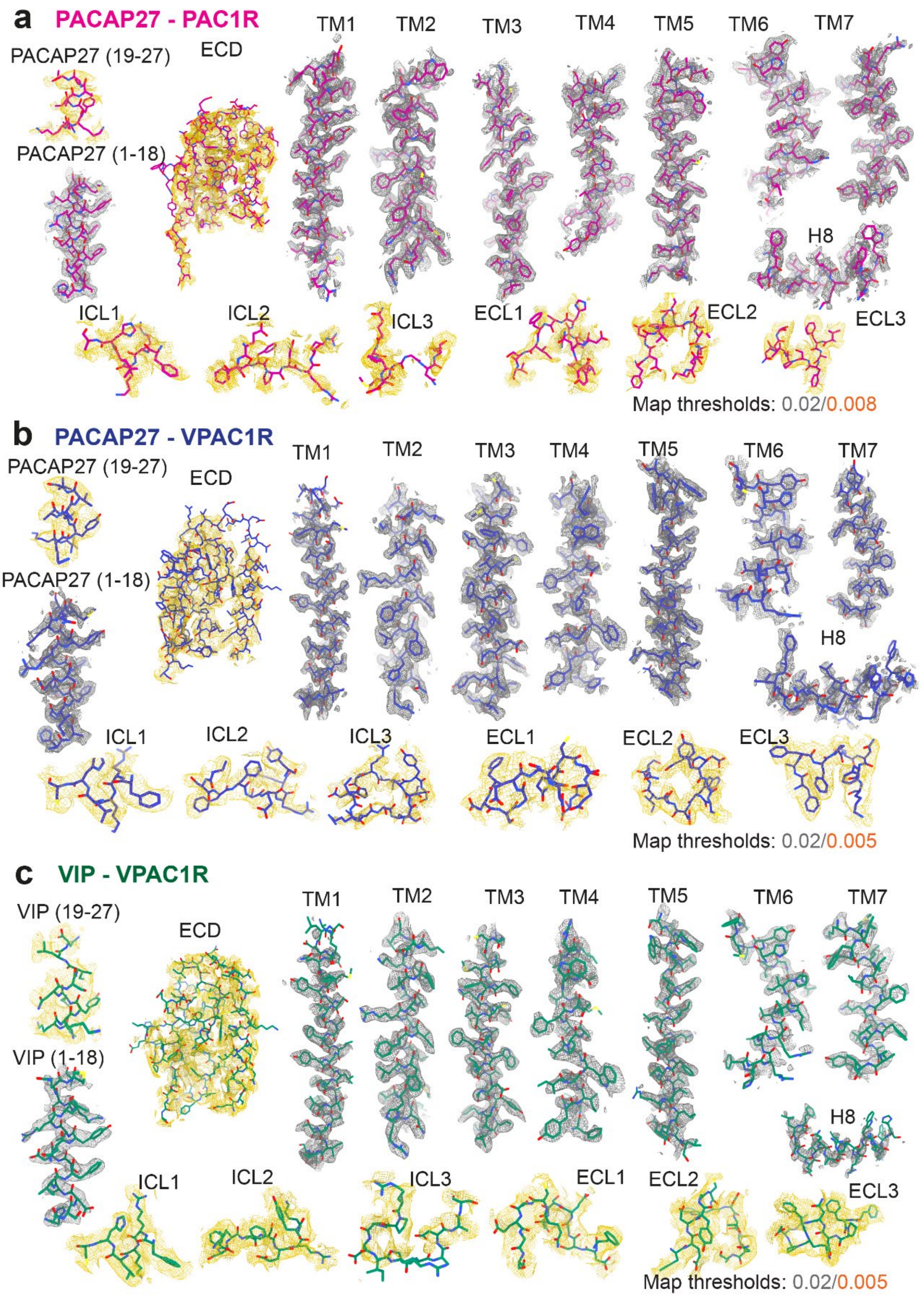

Supplementary Figure 3. Model fit into cryo-EM maps. Maps are displayed as mesh at the indicated volume map threshold in ChimeraX, using the tool 'Surface Zone' with 2 Å radius. The receptor-alone focused map (yellow) was used to build the ECD, peptide C-terminus as well as ECLs and ICLs. The higher resolution consensus map (grey) was used for the TMs, H8 and peptide N-terminus. Models are shown as stick in atom representation. a: PAC1R-PACAP27 (pink), b: VPAC1R-PACAP27 (blue) and c: VPAC1R-VIP (green).

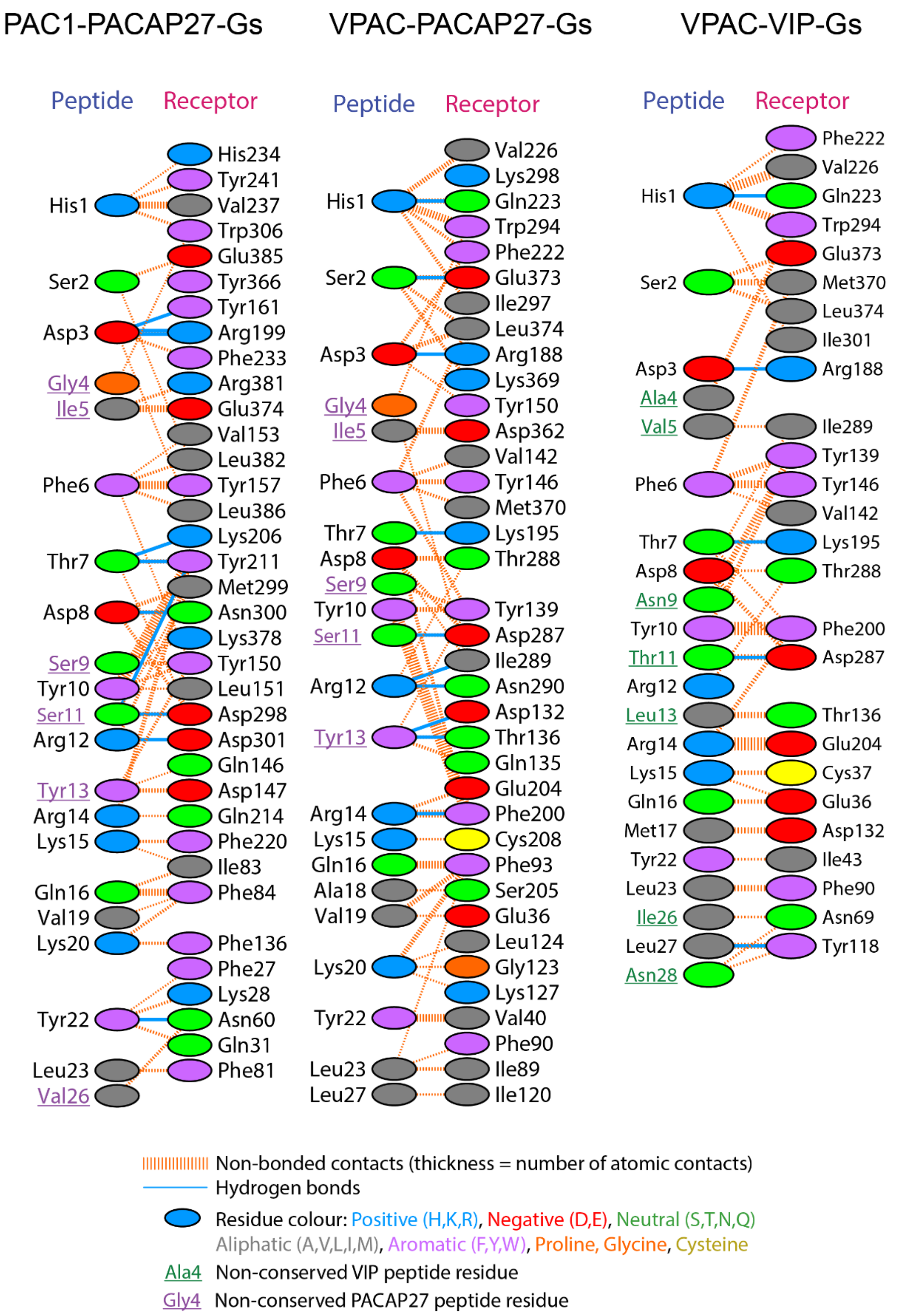

Supplementary Figure 4. Peptide-receptor interaction table. List of generic, van-der-Waals contacts (non-bonded, dotted orange lines) and hydrogen bonds (blue lines) of PAC1R-PACAP27, VPAC1R-PACAP27 and VPAC1R-VIP from the static experimental cryo-EM structures created using Dimplot in Ligplot/LigPlus software. Peptide residues that differ between VIP and PACAP27 are coloured in green and purple, respectively. Residues are represented as oval circles and coloured by residue property/group (positive: blue, negative: red, neutral: green, aliphatic: grey, aromatic: purple, Pro/Gly: orange, Cys: yellow).

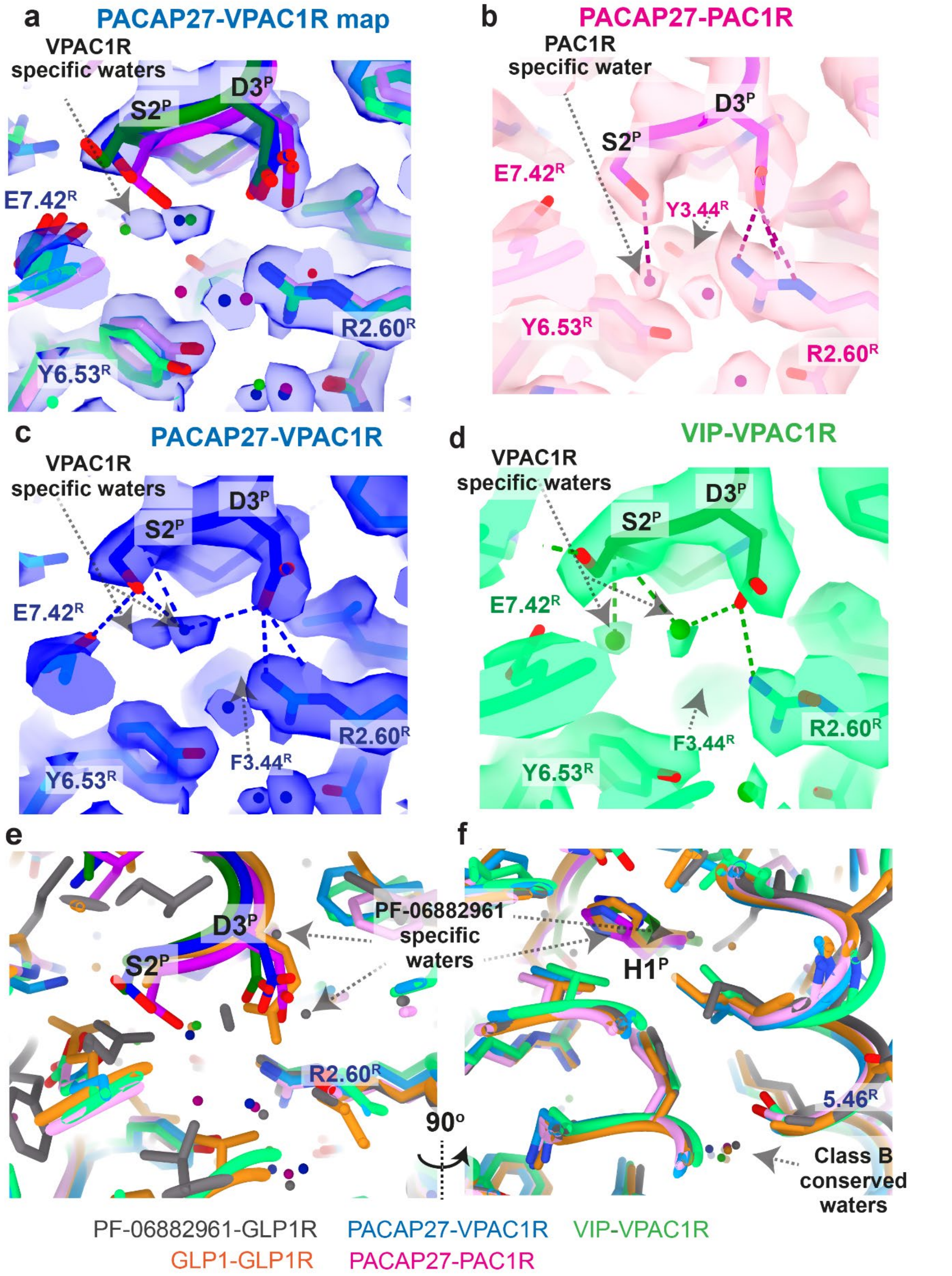

Supplementary Figure 5. Water network near the peptide N-terminus (S2-D3 residues) in the cryo-EM maps. The receptor backbone and peptide backbone are shown in ribbon format, with sidechains displayed in stick format, showing a zoomed-in side view of the map and model environment of the S2-D3 peptide residues. Water molecules are shown as single spheres. PAC1R-PACAP27 is shown in pink-dark pink, VPAC1R-PACAP27 in blue-dark blue

and VPAC1R-VIP in green-dark green, with the cryo-EM consensus maps (post-processed, auto B-factor sharpened with visible densities for water molecules) in the receptor colours shown in transparent. H-bonds involving receptor and peptide residues or waters are shown as dotted lines. Receptor residues are numbered according to the Wootten et al, class B1 scheme. a: Overlay of receptor-aligned models of PAC1R-PACAP27, VPAC1R-VIP and VPAC1R-PACAP27 with the VPAC1R-PACAP27 map (blue transparent) as reference. b-d: Models in their respective maps, of PAC1R-PACAP27 (pink, b), VPAC1R-PACAP27 (blue, c) and VPAC1R-VIP (green, d). e-f: Water network near the peptide N-terminus of GLP1R-GLP1 (orange, PDB 6X18) and below the small molecule binding side of GLP1R-PF-06882961 (grey, PDB 6X1A) as well as PAC1R-PACAP27 (pink), VPAC1R-VIP (green) and VPAC1R-PACAP27 (blue).

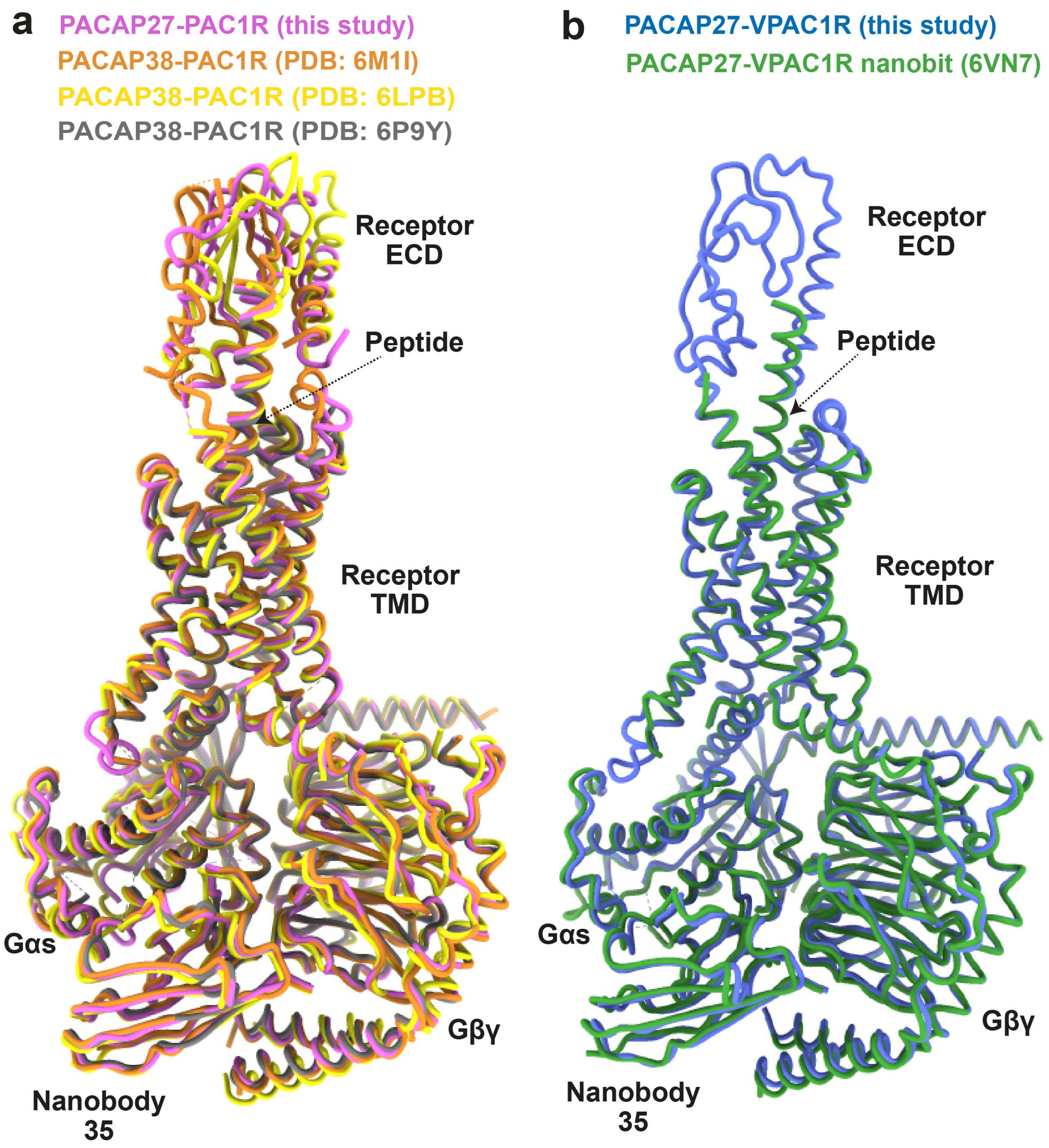

Supplementary Figure 6. Overlay of published PAC1R-PACAP38 and VPAC1R-PACAP27 with structures determined in this study. Models are shown as backbones in ribbon/licorice format. a: PAC1R-PACAP27 (pink) overlaid with available published structures of PAC1R-PACAP38 (PDB 6M1I, orange; PDB 6LPB, yellow; PDB 6P9Y, grey). b: VPAC1R-PACAP27 (blue) overlaid with available published structure of VPAC1R-PACAP27 using the nanobit technology (PDB 6VN7, green).

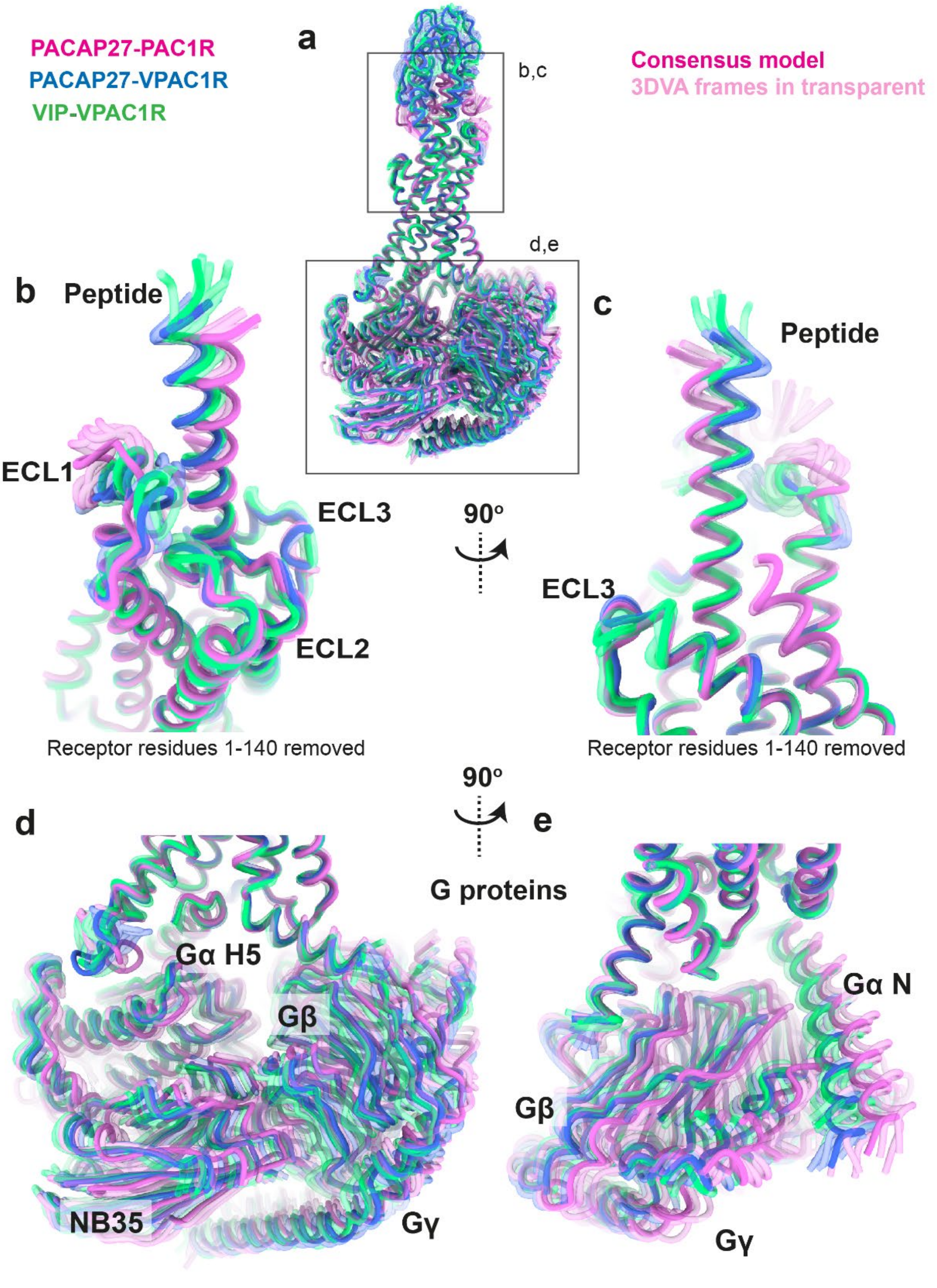

Supplementary Figure 7. Overlay of 3DVA frames aligned by receptor chains. VPAC1R-VIP (green), VPAC1R-PACAP27 (blue) and PAC1R-PACAP27 (pink) frames derived from the cryoSPARC 3D variability analysis (3DVA) were aligned by receptor chains and shown in transparent together with their respective static consensus structure (not transparent). Models are shown as backbones in ribbon/licorice format. The receptor-aligned models show offsets and variability in the peptide C-terminus, ECD as well as G proteins. a: Overview of entire

complex. b-c: Zoom of the peptide and ECLs (with ECD residues removed) as front (b) and side view (c). d-e: Zoom of the G proteins as front (d) and side view (e).

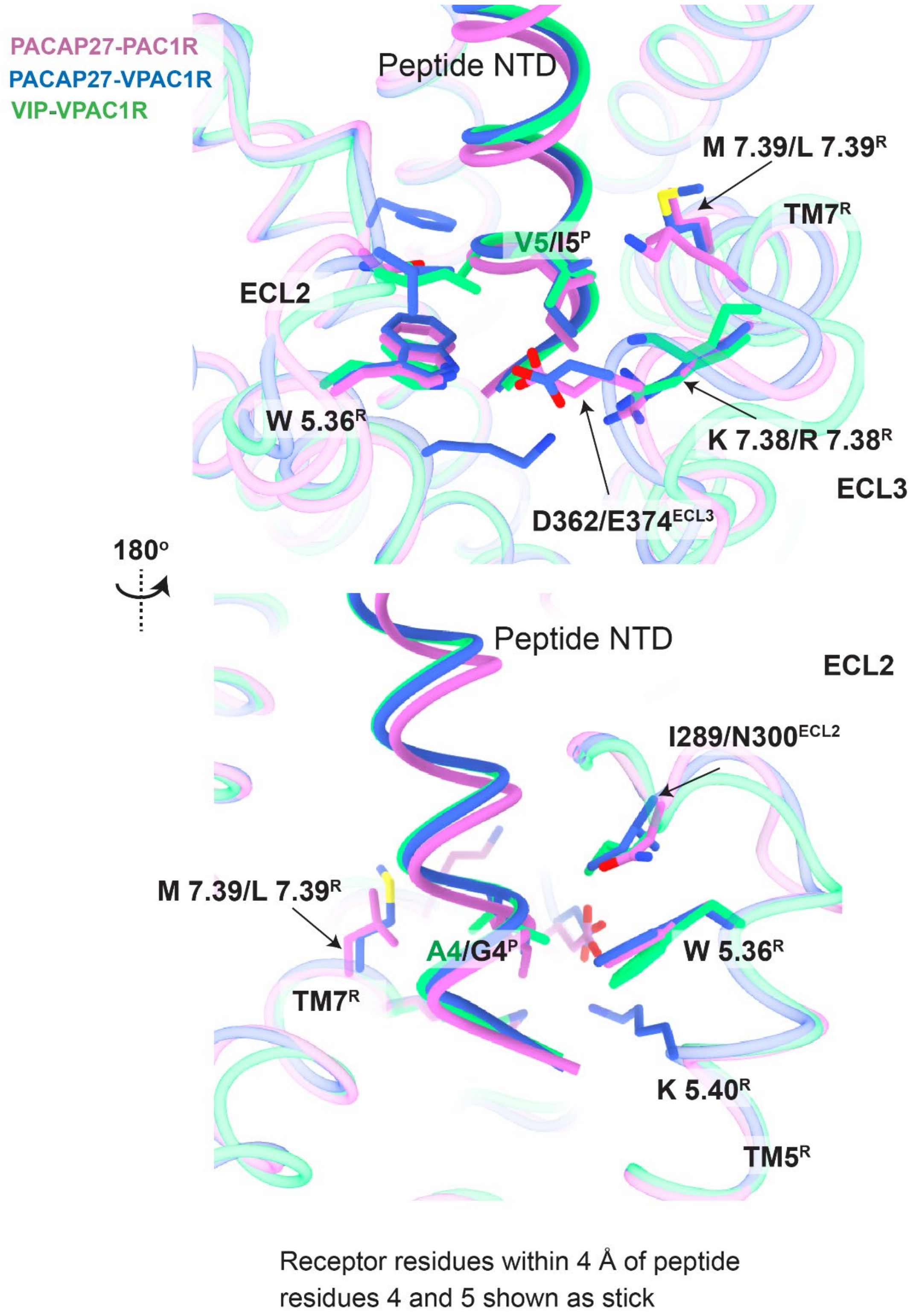

Supplementary Figure 8. Contacts around peptide residues 4 and 5, based on the static experimental structures. Models are shown as backbones in ribbon format, with receptor residues within 4 A of the peptide residues $A4^{VIP}$/$G4^{PACAP27}$ and $V5^{VIP}$/$I5^{PACAP27}$ shown as stick. Models are shown as front (top) and side view (bottom). Receptor residues are numbered according to the Wootten et al, class B1 scheme. Colour of models: VPAC1R-VIP (green), VPAC1R-PACAP27 (blue) and PAC1R-PACAP27 (pink).

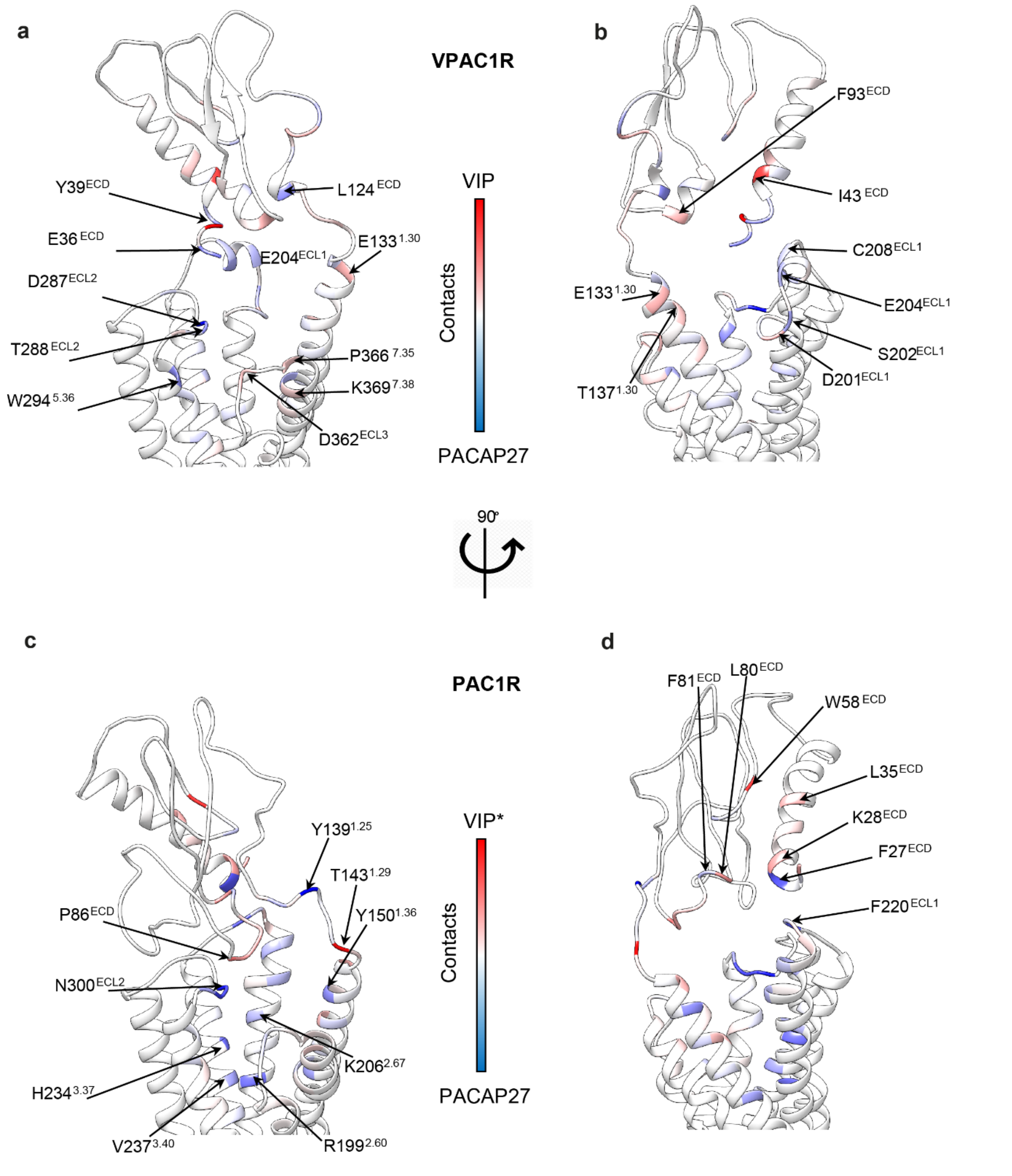

Supplementary Figure 9. Comparison of the generic contacts between PAC1R or VPAC1R and PACAP27 or VIP during MD simulations performed, on the PAC1R-PACAP27, PAC1R-VIP (homology model), VPAC1R-VIP, and VPAC1R-PACAP27 complexes. Residues more involved in contacts with PACAP27 are shown as blue backbone colouring, while residues more involved in contacts with VIP are shown as red backbone colouring. ab: Two-side view of VPAC1R (ribbon representation). c-d: Two-side view of PAC1R (ribbon representation). The PAC1-VIP complex was homology modelled (as indicated by asterisk *) and the MD simulations did not consider the full-length Gs protein (only the Gα H5 was retained). Receptor residues are numbered according to the Wootten et al, class B1 scheme.

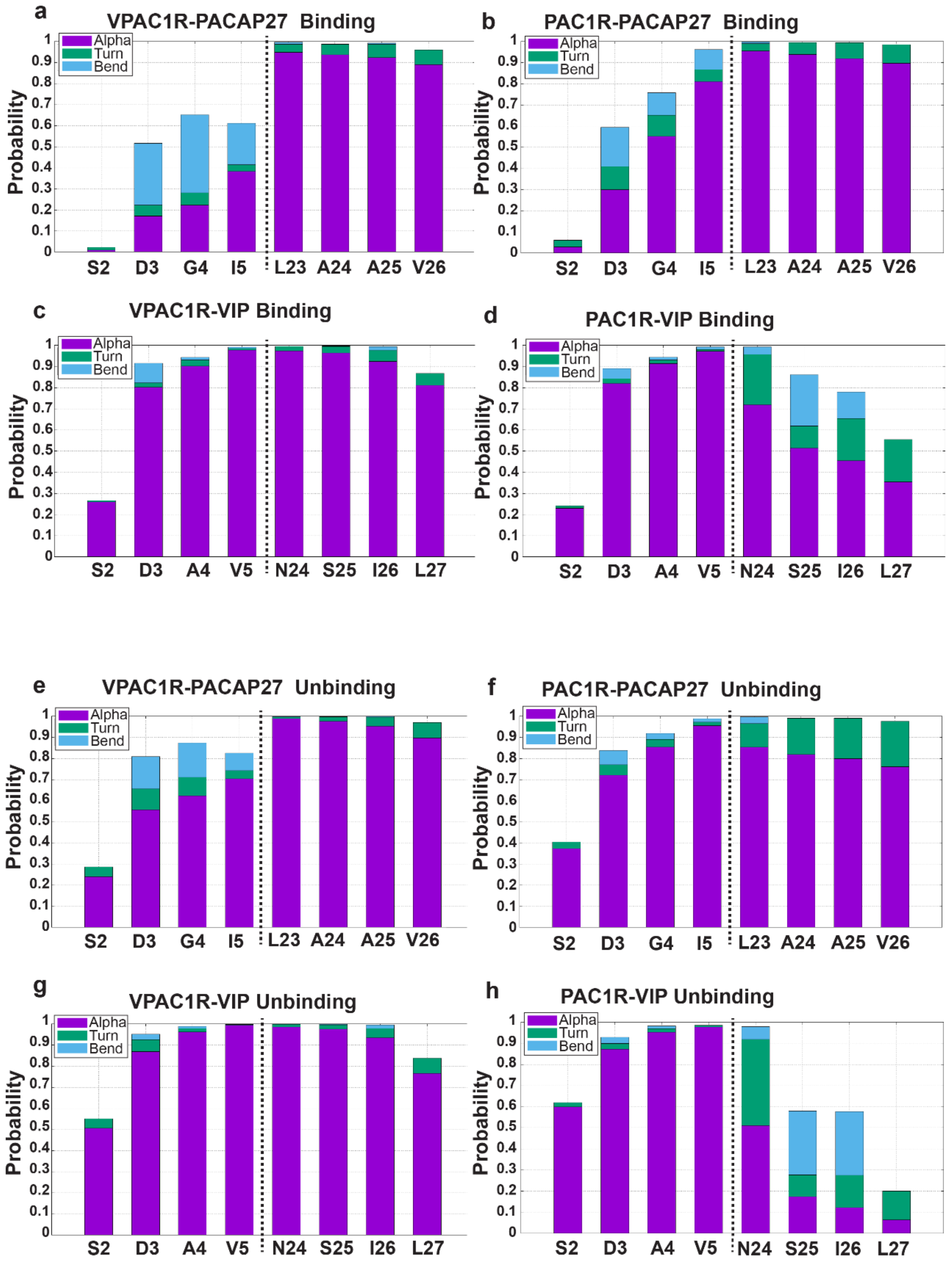

Supplementary Figure 10. DSSP analysis of the peptide residues in position 2-5 (N-terminus) and 23-26/27 (C-terminus) during peptide binding (a-d) and unbinding (e-h) MD simulations. MD simulations were performed on the PAC1R-PACAP27, PAC1R-VIP (homology model), VPAC1R-VIP, and VPAC1R-PACAP27 complexes. Probability of secondary structure of the peptide termini is displayed as height of bar graphs with α helix (purple), turn (green) and bend (blue).

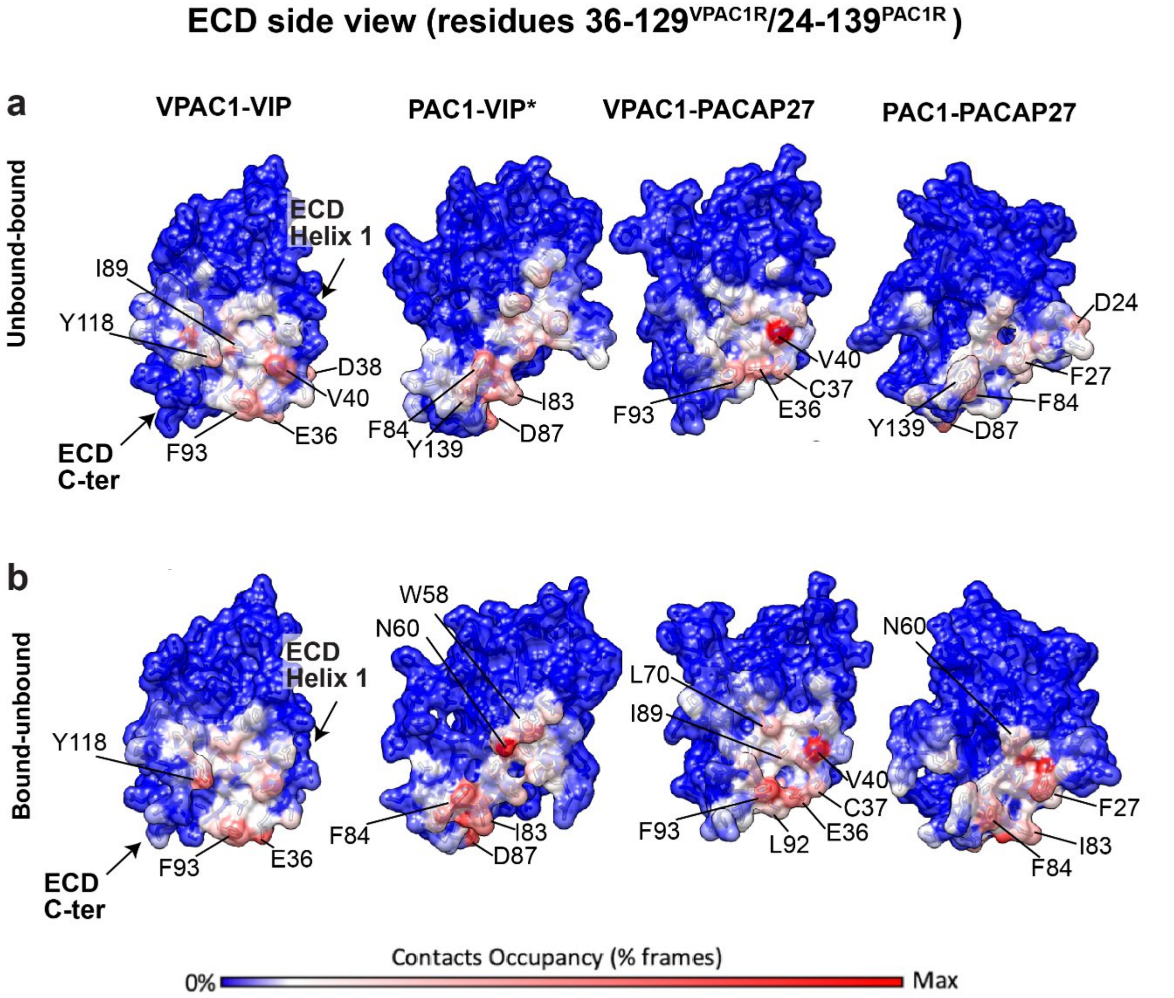

Supplementary Figure 11. Contacts occupancies based on unbinding and binding MD simulations comparing contacts between the peptides VIP and PACAP27 and receptors PAC1R and VPAC1R. **MD simulations were performed on the experimental PAC1R-PACAP27, VPAC1R-VIP and VPAC1R-PACAP27 complexes as well as the PAC1R-VIP (homology model, as indicated by asterisk *).** The total occupancy (% MD frames) for each atom is plotted on the surface of the receptor ECD (a,b) according to a colour scale from 0 % contacts occupancy = blue to maximum contacts occupancy = red. a,b: Side view of receptors PAC1R and VPAC1R (surface representation), showing only the ECD residues (36-129^{VPAC1R}/24-139^{PAC1R}) with occupancies plotted based on the binding simulation (a) or unbinding simulation (b). The PAC1-VIP complex was homology modelled (as indicated by asterisk *)

.

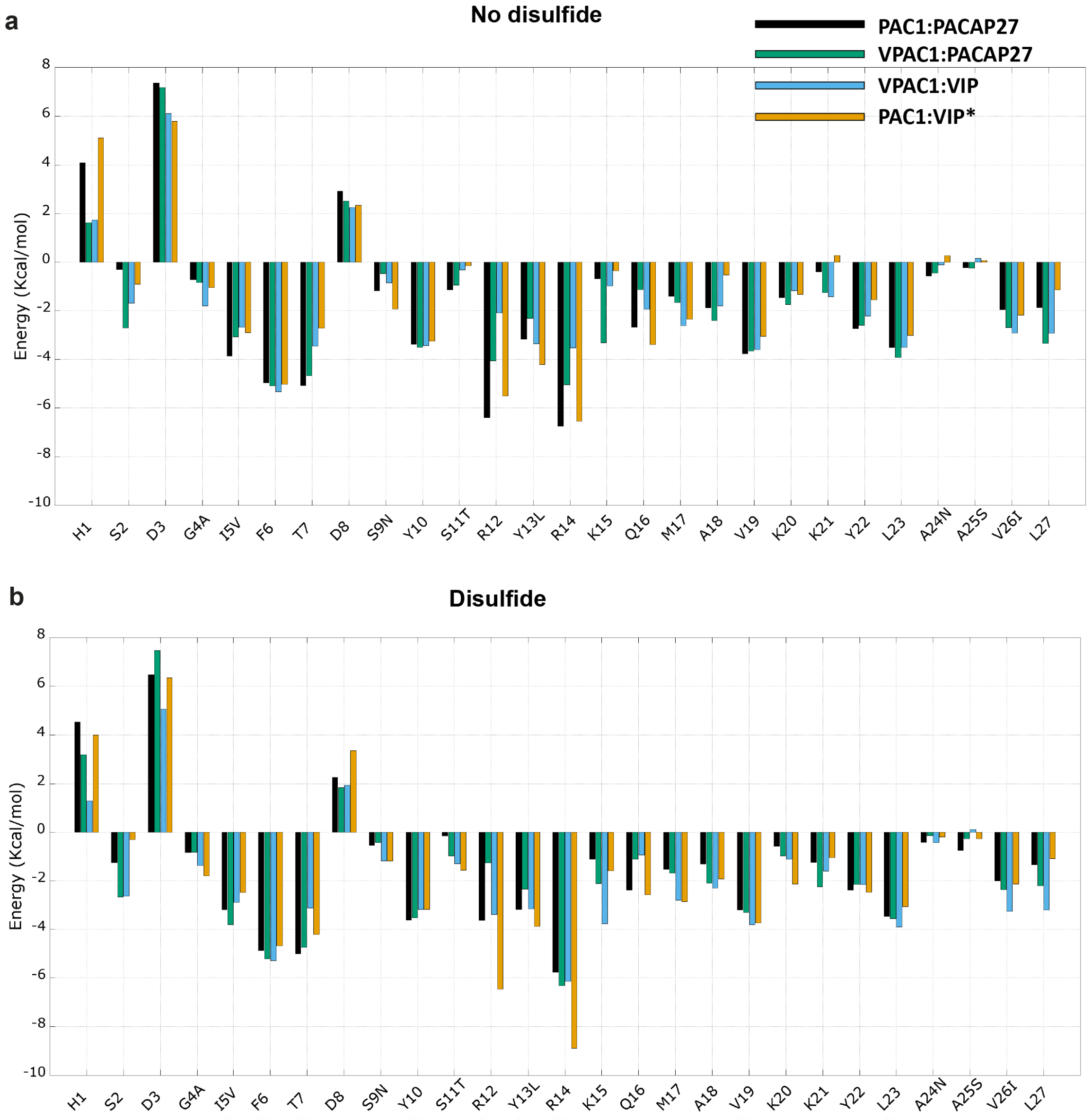

Supplementary Figure 12. MM-GBSA binding energies per peptide residues of VPAC and PAC1 receptor complexes without the ECD-ECL1 disulfide bond (a) and with the disulfide bond present (b). Bar graphs represent peptide residues binding energies in Kcal/mol in the experimental structures of PAC1R-PACAP27 (black), VPAC1R-PACAP27 (green), VPAC1R-VIP (blue), and homology modelled PAC1R-VIP complex (yellow, indicated by asterisk*). MM-GBSA: Molecular Mechanics-Generalized Born Surface Area.

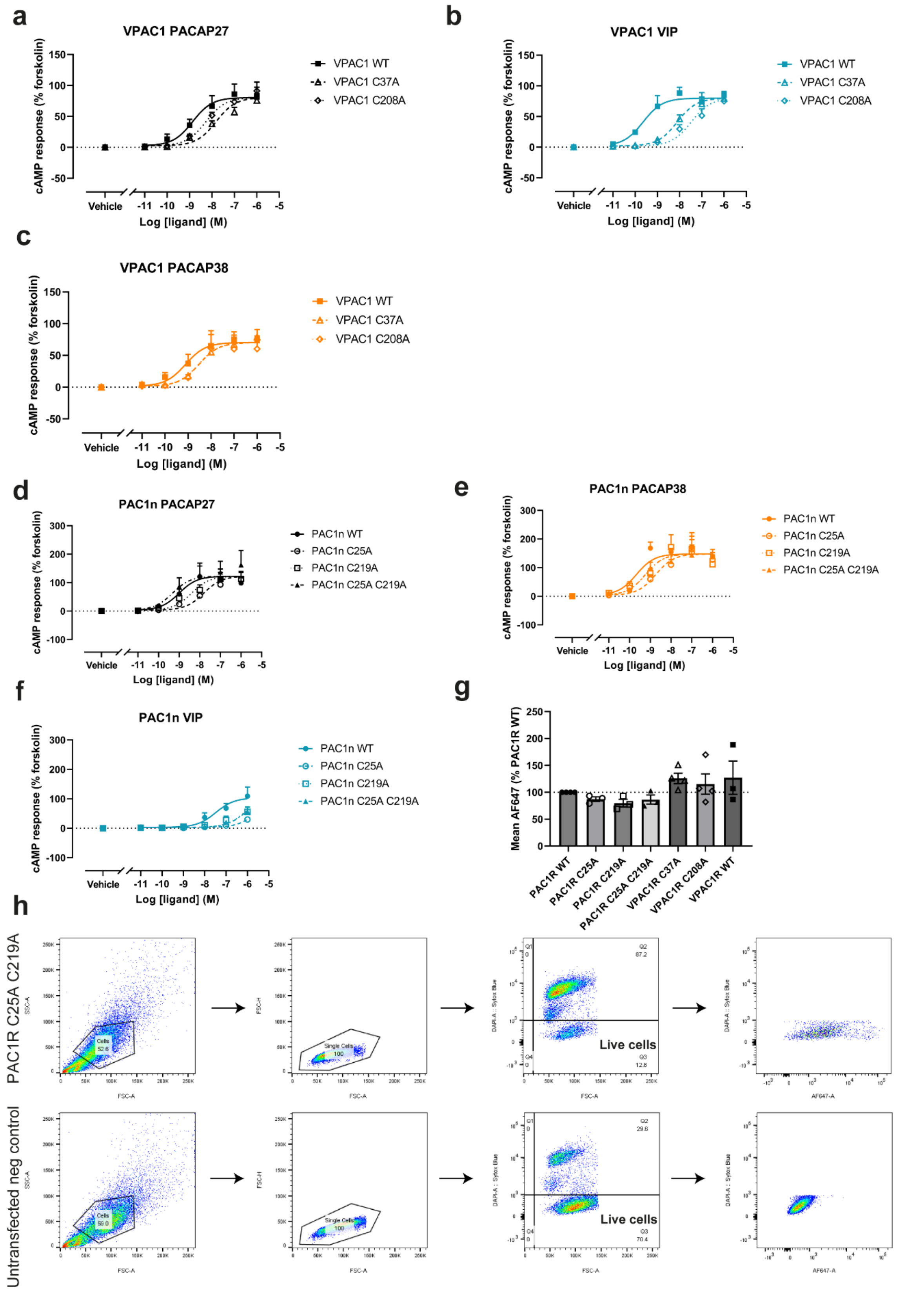

Supplementary Figure 13. Pharmacological analysis of cysteine mutants in cAMP accumulation assay. a-c: Concentration–response data for VPAC1R wild type (WT), C37A,

C208A with PACAP27 (a, black), VIP (b, cyan) and PACAP38 (c, orange). d-f: Concentration–response data for PAC1R WT, C25A, C219A and C25A/C219A with PACAP27 (d, black), PACAP38 (e, orange) and VIP (f, cyan). G: FACS analysis of cell surface expression of Flag tagged wild type and mutants used in the cAMP assays. Mean fluorescence reading (mean AF647) is displayed as scatter plot with bar graphs indicating the average, normalised to untransfected COS-7 control (=0%) and PAC1R wild type expression (=100%). Data in a-g are are mean ± SEM of 3 independent experiments, with the exception of WT PAC1R, VPAC1 C37A and VPAC1R C208A in panel g, which are mean ± SEM of 4 independent experiments. Data were analysed using a one-way ANOVA and Dunnett's post-hoc test with control group being the WT receptor; expression of mutant receptors and WT VPAC1R were not statistically different from the WT PAC1R. h: Gating strategy used for receptor surface expression analysis. Example for PAC1nR C25A C219A (top row) and untransfected COS7 negative control (bottom row). Gating was for single live COS7 cells. Sytox blue was used to discriminate live and dead cells. AF647 levels shown for single live COS7 cells (right column). Source data for panels a-g are provided in the Source Data file.

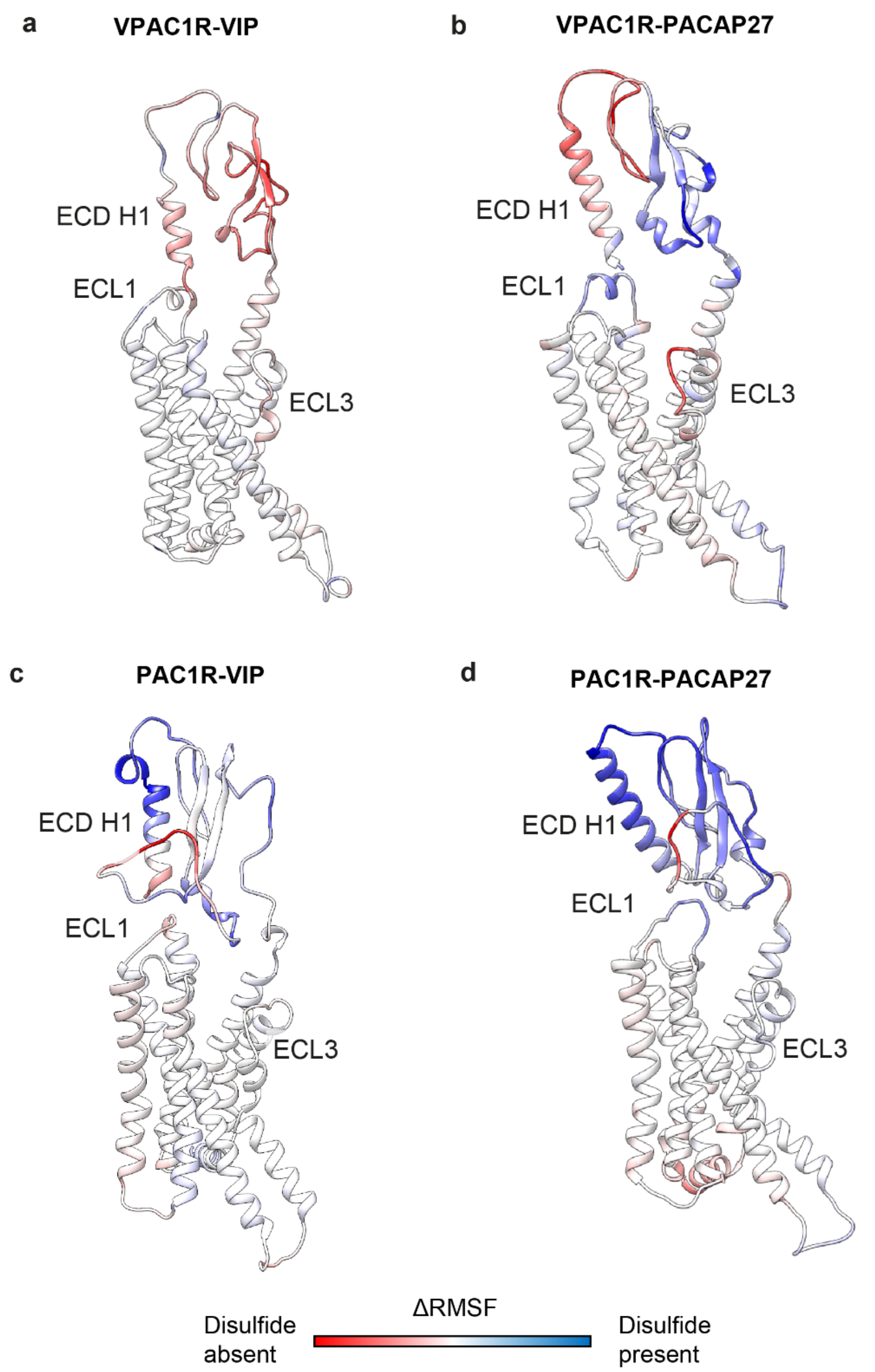

Supplementary Figure 14. RMSF colouring of complexes comparing presence and absence of the ECD-ECL1 disulphide bond during the MD equilibrium simulations. Comparison of the RMSF differences during the MD equilibrium simulations between presence and absence of the ECD-ECL1 disulphide bond performed, on the VPAC1R-VIP (a), VPAC1R-PACAP27 (b), PAC1R-VIP (c) (homology model), and PAC1R-PACAP27 (d). Disulphide bond was either modelled or not modelled between $C25^{ECD}$ - $C219^{ECL1}$ of PAC1R or $C37^{ECD}$ - $C208^{ECL1}$ of VPAC1R. Residues with higher RMSF differences with the disulphide present are shown as blue backbone colouring, while residues with higher RMSF differences with the disulphide bond absent are shown as red backbone colouring, meaning higher structure fluctuations in the presence of the disulphide are coloured in blue, whereas higher structure fluctuations in the absence of the disulphide are coloured in red.

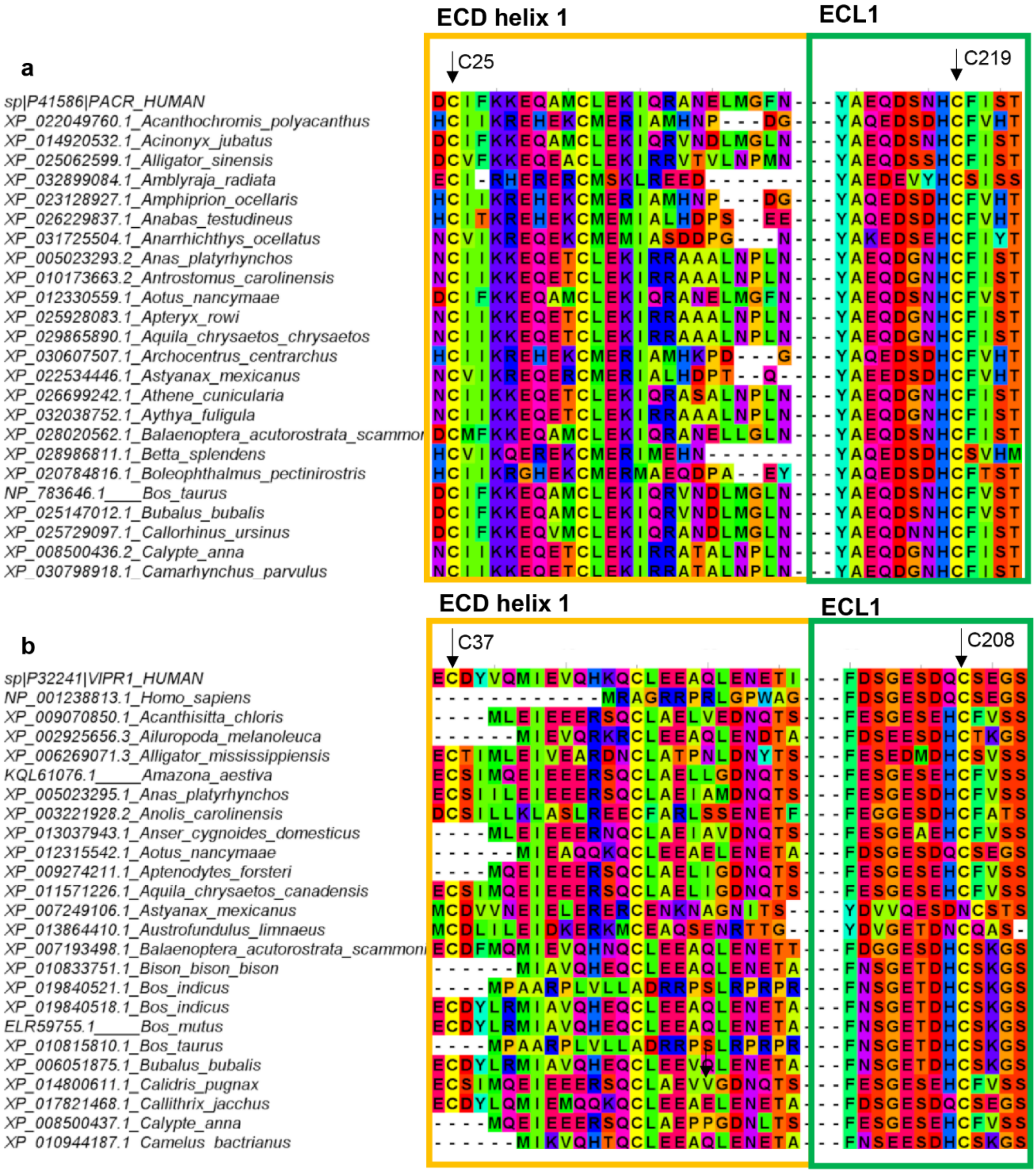

Supplementary Figure 15. PAC1R and VPAC1R multiple sequence alignment for H1 of the ECD (first 41 organisms out of 195). The alignments pf PAC1R (a) and VPAC1R (b) show the N-terminal residues of the ECD for PAC1R in different organisms, with a high conservation of the cysteines within the ECD H1 and ECL1 (yellow background, column is highlighted with a blue arrow). The human sequence of PAC1R is shown in the top row (Uniprot ID: P41586).

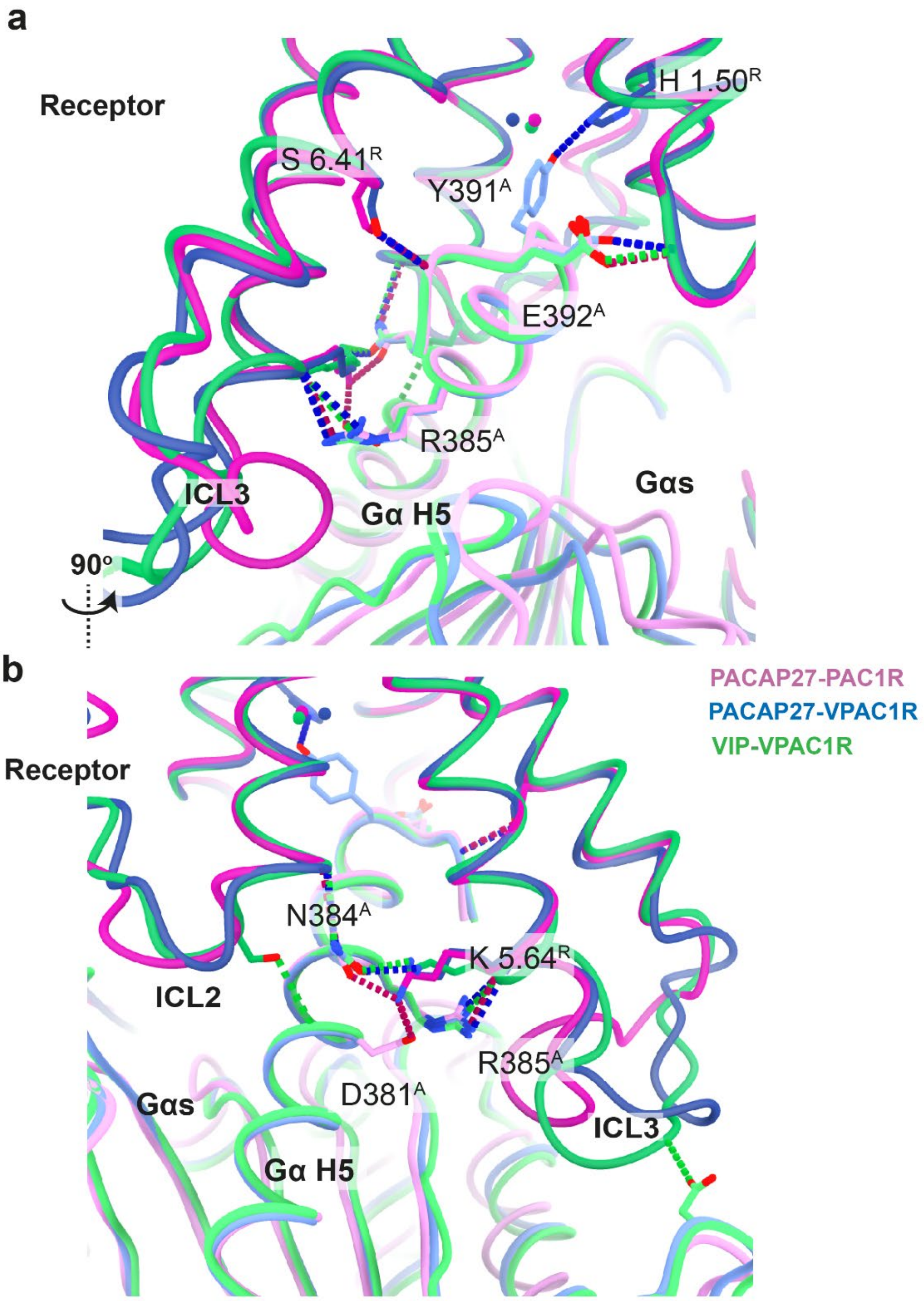

Supplementary Figure 16. Hydrogen bonds between Gαs protein (chain A) and receptor (chain R) residues in the static structures. The Gαs backbone is displayed in transparent in ribbon format and receptor backbone in ribbon format, with sidechains that are involved in H-bonds displayed in stick format. PACAP27-PAC1R-Gs is shown in dark pink - pink, PACAP27-VPAC1R-Gs in dark blue - blue and VIP-VPAC1R-Gs in dark green - green. A: Interactions of the Gs protein and the receptor shown as front view (H5/C-terminus of Gs). B: Interactions of the Gs protein and the receptor shown as back view. H-bonds between peptide and receptor are displayed as dotted lines. H-bonds involving backbones, and not sidechains, are labelled as 'bb'. Receptor residues are numbered according to the Wootten et al, class B1 scheme. Gs residues are labelled according to the residue number.

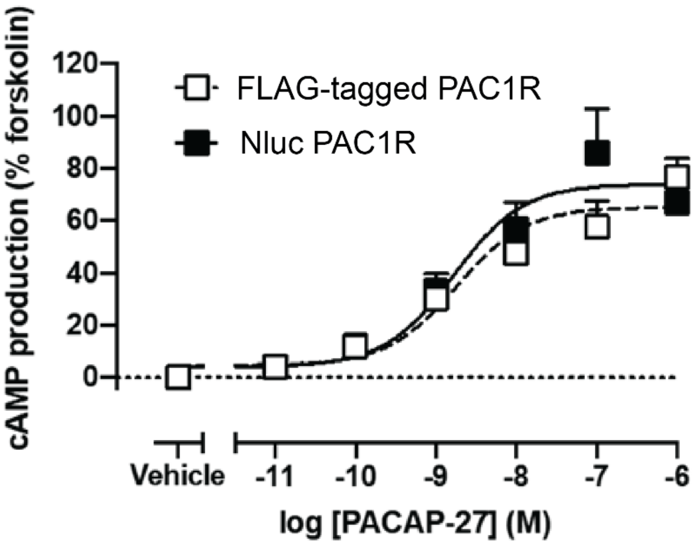

Supplementary Figure 17. Validation of Nanoluc-PAC1R construct for NanoBRET competition binding assay. PACAP27-induced cAMP accumulation comparing N-terminally fused Nanoluc (Nluc)-PAC1R (black squares) to the FLAG tagged PAC1R (white squares). cAMP production is shown as a % of the response to 100 μM forskolin. Data are mean + S.E.M of 4 independent experiments. Source data are provided in the Source Data file.

Supplementary Tables

Supplementary Table 1: Root-mean square deviation (RMSD) of the PAC1R-PACAP27 in this paper compared to (see overlay in Supplementary Figure 6). RMSD calculated using the matchmaker tool in ChimeraX, measuring RMSD between pruned atom pairs and all atom pairs in Å.

**Template**	**Published PDB (PAC1R-PACAP38)**	**Chain**	**RMSD (Å) (pruned)**	**RMSD (Å) (all)**
PAC1R-PACAP27 (this study)	6M1I	Receptor	0.869	2.674
PAC1R-PACAP27 (this study)	6LPB	Receptor	0.719	2.439
PAC1R-PACAP27 (this study)	6P9Y	Receptor	0.589	0.758
PAC1R-PACAP27 (this study)	6M1I	Peptide	0.742	0.742
PAC1R-PACAP27 (this study)	6LPB	Peptide	0.388	0.388
PAC1R-PACAP27 (this study)	6P9Y	Peptide	0.444	0.444
**Template**	**Published PDB (VPAC1R-PACAP27)**	**Chain**	**RMSD (Å) (pruned)**	**RMSD (Å) (all)**
VPAC1R-PACAP27 (this study)	6VN7	Receptor	0.753	0.912
VPAC1R-PACAP27 (this study)	6VN7	Peptide	0.304	0.304
VPAC1R-PACAP27 (this study)	6VN7	Gs	0.686	0.768

Supplementary Table 2: Equilibrium MD simulation details. Summary of the equilibrium MD simulations performed.

Complex	# and Duration MD Replicas	Notes
**PAC1R:PACAP27:Gs:Nb35**	4 x 500 ns (2 μs)	
**VPAC1R:VIP:Gs:Nb35**	4 x 500 ns (2 μs)	
**VPAC1R:PACAP27:Gs:Nb35**	4 x 500 ns (2 μs)	
**PAC1R:VIP:Gs(H5)**	4 x 1000 ns (4 μs)	Homology-modelled; Gs_{α} H5 only

Supplementary Table 3: Generic contacts between receptor and peptide residues during MD equilibrium simulations, with ECD-ECL1 disulphide bond absent (left) and disulphide bond present (right). Columns are sorted by peptide residues and highest to lowest occupancy. Contacts are shown as occupancy of MD frames (in percent), and background is coloured on a scale with highest occupancy in red and lowest occupancy in white. Receptor residues are labelled according to the Wootten numbering system and background is coloured by residue position (TMs, ECLs and ECD, see legend). The PAC1R-VIP simulation is based on a homology model and did not have the full Gs modelled, only the H5 was retained.

MD generic contacts (disulphide bond in ECD-ECL1 absent)											
VPAC1:VIP:Gs			VPAC1:PACAP27:Gs			PAC1:PACAP27:Gs			PAC1:VIP		
E373$^{7.42}$	H1	99.4	E373$^{7.42}$	H1	89.3	E374^{ECL3}	H1	74.0	E374^{ECL3}	H1	74.5
V226$^{3.40}$	H1	46.3	W294$^{5.36}$	H1	73.3	V237$^{3.40}$	H1	63.0	W306$^{5.36}$	H1	43.3
W294$^{5.36}$	H1	29.9	I301$^{5.43}$	H1	57.1				K310$^{5.40}$	H1	25.6
D362^{ECL3}	H1	29.3	V226$^{3.40}$	H1	43.3						
I301$^{5.43}$	H1	26.8									
E373$^{7.42}$	S2	98.7	E373$^{7.42}$	S2	99.5	E385$^{7.42}$	S2	71.9	E385$^{7.42}$	S2	81.6
M370$^{7.39}$	S2	48.6	M370$^{7.39}$	S2	74.0	L386$^{7.43}$	S2	66.0	L382$^{7.39}$	S2	76.0
K369$^{7.38}$	S2	43.3	L374$^{7.43}$	S2	40.8	L382$^{7.39}$	S2	62.6	L386$^{7.43}$	S2	35.4
L374$^{7.43}$	D3	56.6	R188$^{2.60}$	D3	77.0	R199$^{2.60}$	D3	94.9	L386$^{7.43}$	D3	37.4
R188$^{2.60}$	D3	43.7	F222$^{3.36}$	D3	46.0	Y161$^{1.47}$	D3	68.4	Y157$^{1.43}$	D3	29.0
F222$^{3.36}$	D3	37.3	L374$^{7.43}$	D3	43.9	F233$^{3.36}$	D3	63.5			
						L386$^{7.43}$	D3	44.7			
I289^{ECL2}	A4	50.8	I289^{ECL2}	G4	48.3	N300^{ECL2}	G4	29.2	M299^{ECL2}	A4	34.1
W294$^{5.36}$	A4	31.8	W294$^{5.36}$	G4	35.7						
I289^{ECL2}	V5	29.1	M370$^{7.39}$	I5	35.9	L382$^{7.39}$	I5	56.5	L382$^{7.39}$	V5	44.7
			W294$^{5.36}$	I5	33.9	E374^{ECL3}	I5	45.8			
			I289ECL2	I5	30.0	W306$^{5.36}$	I5	36.0			
Y139$^{1.36}$	F6	89.5	L374$^{7.43}$	F6	76.5	V153$^{1.39}$	F6	85.8	Y150$^{1.36}$	F6	69.3
L374$^{7.43}$	F6	72.8	V142$^{1.39}$	F6	67.8	Y157$^{1.43}$	F6	82.8	L386$^{7.43}$	F6	65.3
V142$^{1.39}$	F6	58.1	Y146$^{1.43}$	F6	66.3	Y150$^{1.36}$	F6	79.4	V153$^{1.39}$	F6	60.0
M370$^{7.39}$	F6	57.3	Y139$^{1.36}$	F6	64.8	L386$^{7.43}$	F6	58.2	Y157$^{1.43}$	F6	37.5
Y146$^{1.43}$	F6	46.9	M370$^{7.39}$	F6	59.1				K154$^{1.40}$	F6	30.0
K195$^{2.67}$	T7	58.4	K195$^{2.67}$	T7	60.7	K206$^{2.67}$	T7	85.5	K206$^{2.67}$	T7	40.1
L199$^{2.71}$	T7	52.2	L199$^{2.71}$	T7	48.9	Y211^{ECL1}	T7	40.4	Y211^{ECL1}	T7	37.1
F200^{ECL1}	T7	26.3	F222$^{3.36}$	T7	34.2	F233$^{3.36}$	T7	30.8	L210$^{2.71}$	T7	27.5
F200^{ECL1}	T7	26.3	F200^{ECL1}	T7	25.5						
I289^{ECL2}	D8	62.1	T288^{ECL1}	D8	84.5	N300^{ECL2}	D8	85.8	M299^{ECL2}	D8	30.0
T288^{ECL2}	D8	39.5	I289^{ECL1}	D8	84.4	M299^{ECL2}	D8	80.3			
			D287^{ECL2}	D8	30.2	D298^{ECL2}	D8	41.9			
Y139$^{1.36}$	N9	52.0	Y139$^{1.36}$	S9	76.9	Y150$^{1.36}$	S9	76.5	Y150$^{1.36}$	N9	65.0
									D87^{ECD}	N9	27.2
Y139$^{1.36}$	Y10	84.8	F200^{ECL1}	Y10	85.5	Y150$^{1.36}$	Y10	83.8	Y150$^{1.36}$	Y10	69.8
F200^{ECL1}	Y10	74.6	Y139$^{1.36}$	Y10	81.3	Y211^{ECL1}	Y10	72.0	Y211^{ECL1}	Y10	65.1
G140$^{1.37}$	Y10	33.0	K143$^{1.40}$	Y10	33.3				K154$^{1.40}$	Y10	36.6
F200^{ECL1}	T11	50.5	D287^{ECL2}	S11	93.0	D298^{ECL2}	S11	79.8			
			F200^{ECL1}	S11	42.2	Y211^{ECL1}	S11	61.0			
						M299^{ECL2}	S11	40.4			
T288^{ECL2}	R12	28.6	E36^{ECD}	R12	63.7	M299^{ECL2}	R12	68.0	I83^{ECD}	R12	73.3
			T288^{ECL2}	R12	31.5	D301^{ECL2}	R12	60.1	D87^{ECD}	R12	55.4
									N85^{ECD}	R12	48.3
									F84^{ECD}	R12	39.3
T136$^{1.33}$	L13	74.5	T136$^{1.33}$	Y13	84.1	Y150$^{1.36}$	Y13	95.7	Q146$^{1.32}$	L13	56.9
Y139$^{1.36}$	L13	64.6	Y139$^{1.36}$	Y13	81.9	D147$^{1.33}$	Y13	92.0	Y150$^{1.36}$	L13	55.2
D132$^{1.29}$	L13	48.1	Q135$^{1.32}$	Y13	54.9	Q146$^{1.32}$	Y13	64.3	D147$^{1.33}$	L13	47.8
Q135$^{1.35}$	L13	47.2	D132$^{1.29}$	Y13	48.4	T143$^{1.29}$	Y13	31.7	T143$^{1.29}$	L13	43.0

MD generic contacts (disulphide bond in ECD-ECL1 present)											
VPAC1:VIP:Gs			VPAC1:PACAP27:Gs			PAC1:PACAP27:Gs			PAC1:VIP		
E373$^{7.42}$	H1	91.4	W294$^{5.36}$	H1	73.0	Y241$^{3.44}$	H1	68.7	E385$^{7.42}$	H1	66.7
I301$^{5.43}$	H1	34.9	I301$^{5.43}$	H1	63.5	E385$^{7.42}$	H1	65.6	V237$^{3.40}$	H1	50.6
D362^{ECL3}	H1	34.3	E373$^{7.42}$	H1	62.0	V313$^{5.43}$	H1	49.5	E374^{ECL3}	H1	46.4
K298$^{5.40}$	H1	29.5	V226$^{3.40}$	H1	54.8	V237$^{3.40}$	H1	39.8	V313$^{5.43}$	H1	44.8
									Y241$^{3.44}$	H1	29.8
E373$^{7.42}$	S2	99.7	E373$^{7.42}$	S2	99.5	E385$^{7.42}$	S2	79.1	E385$^{7.42}$	S2	81.5
M370$^{7.39}$	S2	56.8	K369$^{7.38}$	S2	56.0	L386$^{7.43}$	S2	68.5	L386$^{7.43}$	S2	63.9
L374$^{7.43}$	S2	46.6	L374$^{7.43}$	S2	43.3	L382$^{7.39}$	S2	60.6	L382$^{7.39}$	S2	46.6
K369$^{7.38}$	S2	45.7	M370$^{7.39}$	S2	42.0						
L374$^{7.43}$	D3	54.9	R188$^{2.60}$	D3	81.1	R199$^{2.60}$	D3	93.5	R199$^{2.60}$	D3	91.8
R188$^{2.60}$	D3	45.9	L374$^{7.43}$	D3	59.7	Y161$^{1.47}$	D3	75.3	Y161$^{1.47}$	D3	67.7
F222$^{3.36}$	D3	30.8	F222$^{3.36}$	D3	55.8	F233$^{3.36}$	D3	46.0	F233$^{3.36}$	D3	52.8
						L386$^{7.43}$	D3	31.7	L386$^{7.43}$	D3	29.0
W294$^{5.36}$	A4	37.8	I289^{ECL2}	G4	52.3				W306$^{5.36}$	A4	30.8
I289^{ECL2}	A4	36.5	W294$^{5.36}$	G4	30.0						
D362^{ECL3}	V5	44.6	D362^{ECL3}	I5	44.1	L382$^{7.39}$	I5	40.5	L382$^{7.39}$	V5	41.3
			W294$^{5.36}$	I5	33.8				E374^{ECL3}	V5	27.7
			I289^{ECL2}	I5	28.2						
Y139$^{1.36}$	F6	83.4	L374$^{7.43}$	F6	80.5	Y157$^{1.43}$	F6	87.2	Y150$^{1.36}$	F6	73.4
L374$^{7.43}$	F6	68.5	Y139$^{1.36}$	F6	73.3	V153$^{1.39}$	F6	86.2	L386$^{7.43}$	F6	72.5
V142$^{1.39}$	F6	63.5	Y146$^{1.43}$	F6	71.2	Y150$^{1.36}$	F6	77.6	Y157$^{1.43}$	F6	71.3
M370$^{7.39}$	F6	57.2	V142$^{1.39}$	F6	68.2	L386$^{7.43}$	F6	40.8	V153$^{1.39}$	F6	54.9
Y146$^{1.43}$	F6	43.9	M370$^{7.39}$	F6	66.7						
K195$^{2.67}$	T7	51.0	K195$^{2.67}$	T7	68.4	K206$^{2.67}$	T7	86.3	K206$^{2.67}$	T7	87.0
L199$^{2.71}$	T7	31.1	F200^{ECL1}	T7	39.4	F233$^{3.36}$	T7	42.0	F233$^{3.36}$	T7	33.4
			F222$^{3.36}$	T7	35.6	L210$^{2.71}$	T7	40.6	L210$^{2.71}$	T7	28.1
			L199$^{2.71}$	T7	25.3	Y211^{ECL1}	T7	28.2			
I289^{ECL2}	D8	53.1	I289^{ECL2}	D8	90.7	N300^{ECL2}	D8	80.8			
T288^{ECL2}	D8	38.3	T288^{ECL2}	D8	90.6	M299^{ECL2}	D8	47.6			
			D287^{ECL2}	D8	39.1						
Y139$^{1.36}$	N9	68.2	Y139$^{1.36}$	S9	72.5	Y150$^{1.36}$	S9	59.6	Y150$^{1.36}$	N9	52.9
Y139$^{1.36}$	Y10	79.2	F200^{ECL1}	Y10	85.1	Y150$^{1.36}$	Y10	92.2	Y211^{ECL1}	Y10	72.8
F200^{ECL1}	Y10	70.7	Y139$^{1.36}$	Y10	80.1	Y211^{ECL1}	Y10	92.1	Y150$^{1.36}$	Y10	67.5
K143$^{1.40}$	Y10	33.8	K143$^{1.40}$	Y10	29.3				K154$^{1.40}$	Y10	39.0
D287^{ECL2}	T11	47.3	D287^{ECL2}	S11	94.6	D298^{ECL2}	S11	68.8	Y211^{ECL1}	T11	79.5
F200^{ECL1}	T11	39.2	F200^{ECL1}	S11	63.0	Y211^{ECL1}	S11	38.0	D298^{ECL2}	T11	36.9
						M299^{ECL2}	S11	27.8			
E36^{ECD}	R12	42.2	T288^{ECL2}	R12	51.9	M299^{ECL2}	R12	54.0	D87^{ECD}	R12	65.3
						F84^{ECD}	R12	31.1	I83^{ECD}	R12	36.4
									M299^{ECL2}	R12	35.1
T136$^{1.33}$	L13	67.9	Y139$^{1.36}$	Y13	79.1	Y150$^{1.36}$	Y13	92.6	T143$^{1.29}$	L13	59.0
Y139$^{1.36}$	L13	52.8	T136$^{1.33}$	Y13	78.4	D147$^{1.33}$	Y13	84.1	Q146$^{1.32}$	L13	56.3
Q135$^{1.35}$	L13	46.7	D132$^{1.29}$	Y13	52.7	Q146$^{1.32}$	Y13	74.3	Y150$^{1.36}$	L13	54.8
D132$^{1.29}$	L13	37.7	Q135$^{1.32}$	Y13	51.9	T143$^{1.29}$	Y13	30.0	D147$^{1.33}$	L13	47.2

TM1
TM2
TM3
TM4
TM5
TM6
TM7
ECD
ECL1
ECL2
ECL3

Min %

Max %

Supplementary Table 3 continued

MD generic contacts (disulphide bond in ECD-ECL1 absent)												MD generic contacts (disulphide bond in ECD-ECL1 present)											
VPAC1:VIP:Gs			VPAC1:PACAP27:Gs			PAC1:PACAP27:Gs			PAC1:VIP			VPAC1:VIP:Gs			VPAC1:PACAP27:Gs			PAC1:PACAP27:Gs			PAC1:VIP		
F200^{ECL1}	R14	48.1	S202^{ECL1}	R14	68.9	D215^{ECL1}	R14	98.0	D215^{ECL1}	R14	72.4	E204^{ECL1}	R14	83.2	F200^{ECL1}	R14	75.2	Y211^{ECL1}	R14	49.4	D215^{ECL1}	R14	68.1
D201^{ECL1}	R14	43.5	F200^{ECL1}	R14	62.0	Y211^{ECL1}	R14	57.8	Y211^{ECL1}	R14	60.5	S202^{ECL1}	R14	73.8	S205^{ECL1}	R14	48.1	E213^{ECL1}	R14	40.9	Y211^{ECL1}	R14	55.8
E204^{ECL1}	R14	43.3	E204^{ECL1}	R14	52.5	Q214^{ECL1}	R14	33.7				Q207^{ECL1}	R14	72.4	L199$^{2.71}$	R14	37.4	D215^{ECL1}	R14	33.5	D147$^{1.33}$	R14	54.7
Q207^{ECL1}	R14	41.6	Q207^{ECL1}	R14	39.3							F200^{ECL1}	R14	71.0	E204^{ECL1}	R14	35.5						
G203^{ECL1}	R14	33.5	G203^{ECL1}	R14	25.6							G203^{ECL1}	R14	49.7	G203^{ECL1}	R14	33.7						
												L199$^{2.71}$	R14	26.7	S202^{ECL1}	R14	31.4						
															E210^{ECL1}	R14	29.2						
Q207^{ECL1}	K15	51.6	Q207^{ECL1}	K15	81.6	F220^{ECL1}	K15	67.9	I83^{ECD}	K15	30.6	E36^{ECD}	K15	74.6	E36^{ECD}	K15	46.2	D298^{ECL2}	K15	54.8	D298^{ECL2}	K15	79.3
C208^{ECL1}	K15	38.9	D287^{ECL2}	K15	54.1	D298^{ECL2}	K15	39.2	F84^{ECD}	K15	27.5	D287^{ECL2}	K15	64.3	C208^{ECL1}	K15	30.0	M299^{ECL2}	K15	39.5	F220^{ECL1}	K15	63.2
Y39^{ECD}	K15	36.2	C37^{ECD}	K15	34.5	I83^{ECD}	K15	38.5				Q207^{ECL1}	K15	45.6	Y283^{ECL2}	K15	29.8	F84^{ECD}	K15	33.0	I83^{ECD}	K15	39.2
E36^{ECD}	K15	29.3	Y283^{ECL2}	K15	26.4							C37^{ECD}	K15	42.1									
F93^{ECD}	Q16	67.1	E36^{ECD}	Q16	41.2	F84^{ECD}	Q16	83.1	F84^{ECD}	Q16	84.4	F93^{ECD}	Q16	49.0	F93^{ECD}	Q16	35.8	F84^{ECD}	Q16	74.8	F84^{ECD}	Q16	66.6
Y39^{ECD}	Q16	40.6	F93^{ECD}	Q16	39.4	I83^{ECD}	Q16	59.0	T143$^{1.29}$	Q16	44.8				E36^{ECD}	Q16	33.6	I83^{ECD}	Q16	37.4	I83^{ECD}	Q16	47.4
									F136^{ECD}	Q16	41.9							N85^{ECD}	Q16	27.2	T143$^{1.29}$	Q16	33.8
									I83^{ECD}	Q16	40.4							F136^{ECD}	Q16	26.2			
T136$^{1.33}$	M17	34.5	D132$^{1.29}$	M17	33.9				T143$^{1.29}$	M17	67.3	D132$^{1.29}$	M17	37.0				T143$^{1.29}$	M17	28.3	T143$^{1.29}$	M17	70.8
D132$^{1.29}$	M17	34.2							D147$^{1.33}$	M17	43.4	E204^{ECL1}	M17	25.1							D147$^{1.33}$	M17	38.4
E133$^{1.30}$	M17	27.1																					
E204^{ECL1}	A18	41.6	E204^{ECL1}	A18	72.7	F220^{ECL1}	A18	33.0				E204^{ECL1}	A18	60.9	S205^{ECL1}	A18	48.4				F220^{ECL1}	A18	33.7
C208^{ECL1}	A18	32.3	Q207^{ECL1}	A18	59.1	F27^{ECD}	A18	30.2				S205^{ECL1}	A18	28.9	Q207^{ECL1}	A18	39.0				H218^{ECL1}	A18	25.3
			C208^{ECL1}	A18	43.0							Q207^{ECL1}	A18	28.2	C208^{ECL1}	A18	25.9						
Y39^{ECD}	V19	51.7	C208^{ECL1}	V19	41.4	F27^{ECD}	V19	49.0	L80^{ECD}	V19	53.6	F93^{ECD}	V19	54.9	F93^{ECD}	V19	43.6	L80^{ECD}	V19	38.8	L80^{ECD}	V19	50.8
F93^{ECD}	V19	39.5	V40^{ECD}	V19	40.8	F84^{ECD}	V19	46.5	F84^{ECD}	V19	32.5	Y39^{ECD}	V19	52.5	E36^{ECD}	V19	36.7	F81^{ECD}	V19	38.0	F81^{ECD}	V19	45.4
I43^{ECD}	V19	38.8	F93^{ECD}	V19	31.6	F81^{ECD}	V19	44.6				I43^{ECD}	V19	37.9	C37^{ECD}	V19	35.7	F84^{ECD}	V19	37.3	F84^{ECD}	V19	45.1
			I89^{ECD}	V19	26.9							V40^{ECD}	V19	37.3									
			E36^{ECD}	V19	26.8																		
F93^{ECD}	K20	48.2	D132$^{1.29}$	K20	71.8	Y139$^{1.25}$	K20	56.3	E138^{ECD}	K20	53.3	F93^{ECD}	K20	54.2	D132$^{1.29}$	K20	65.8	Y139$^{1.25}$	K20	48.4	T143$^{1.29}$	K20	32.2
D132$^{1.29}$	K20	31.2	F93^{ECD}	K20	47.4	D137^{ECD}	K20	45.3	T143$^{1.29}$	K20	36.6	D132$^{1.29}$	K20	36.8	F93^{ECD}	K20	37.4				E138	K20	29.4
						E138^{ECD}	K20	41.5													E140$^{1.26}$	K20	28.5
E204^{ECL1}	K21	54.9	E204^{ECL1}	K21	56.8							E204^{ECL1}	K21	44.1	E204^{ECL1}	K21	56.5				H218^{ECL1}	K21	45.8
															D206^{ECL1}	K21	50.3				D215^{ECL1}	K21	33.8
															S205^{ECL1}	K21	28.3						
I43^{ECD}	Y22	73.3	V40^{ECD}	Y22	54.8	F27^{ECD}	Y22	82.4	K28^{ECD}	Y22	55.8	I43^{ECD}	Y22	70.3	V40^{ECD}	Y22	65.0	F27^{ECD}	Y22	61.5	F27^{ECD}	Y22	56.4
E44^{ECD}	Y22	47.6	E44^{ECD}	Y22	47.6	Q31^{ECD}	Y22	39.3				E44^{ECD}	Y22	38.8	E44^{ECD}	Y22	45.8	I26^{ECD}	Y22	55.0	K28^{ECD}	Y22	44.7
H47^{ECD}	Y22	43.5	C208^{ECL1}	Y22	32.1	K28^{ECD}	Y22	34.0				V40^{ECD}	Y22	37.3	I89^{ECD}	Y22	28.5	D24^{ECD}	Y22	39.9	Q31^{ECD}	Y22	40.5
			I43^{ECD}	Y22	27.7	C25^{ECD}	Y22	28.9				H47^{ECD}	Y22	30.3				W58^{ECD}	Y22	36.8	N60^{ECD}	Y22	35.7
																					H218^{ECL1}	Y22	29.1
I89^{ECD}	L23	31.4	F90^{ECD}	L23	45.8	F136^{ECD}	L23	51.7	N60^{ECD}	L23	52.4	F90^{ECD}	L23	46.5	I89^{ECD}	L23	55.8	Y139$^{1.25}$	L23	40.4	N60^{ECD}	L23	52.0
F93^{ECD}	L23	25.0	I89^{ECD}	L23	39.6	I61^{ECD}	L23	37.0	W58^{ECD}	L23	32.8	F93^{ECD}	L23	32.1	F90^{ECD}	L23	55.4	F136^{ECD}	L23	38.5			
			L124^{ECD}	L23	30.3	F131^{ECD}	L23	32.8				I43^{ECD}	L23	30.8	F93^{ECD}	L23	38.8	F81^{ECD}	L23	32.3			
						Y139$^{1.25}$	L23	31.8				I89^{ECD}	L23	28.7	L124^{ECD}	L23	25.2	I61^{ECD}	L23	27.4			
Y118^{ECD}	N24	25.9				Y139$^{1.25}$	A24	42.1															
Y118^{ECD}	N24	25.9																					
H47^{ECD}	I26	41.8	N69^{ECD}	V26	32.1	N60^{ECD}	V26	56.7	W58^{ECD}	I26	44.0	H47^{ECD}	I26	41.8	L70^{ECD}	V26	37.8	F27^{ECD}	V26	34.8	W58^{ECD}	I26	31.9
I43^{ECD}	I26	25.8	L70^{ECD}	V26	27.6							N69^{ECD}	I26	40.7									
I43^{ECD}	I26	25.8										I43^{ECD}	I26	30.6									
												W67^{ECD}	I26	25.5									
Y118^{ECD}	L27	26.9	P117^{ECD}	L27	29.5	F127^{ECD}	L27	36.1	W58^{ECD}	L27	31.4	N69^{ECD}	L27	40.0				Y139$^{1.25}$	L27	30.1			
N69^{ECD}	L27	26.3	I120^{ECD}	L27	29.1																		

Supplementary Table 4: Hydrogen bonds between receptor and peptide residues during MD equilibrium simulations, with ECD-ECL1 disulphide bond absent (left) and disulphide bond present (right). Columns are sorted by peptide residues and highest to lowest occupancy. Contacts are shown as occupancy of MD frames (in percent), and background is coloured on a scale with highest occupancy in red and lowest occupancy in white. Receptor residues are labelled according to the Wootten numbering system and background is coloured by residue position (TMs, ECLs and ECD, see legend). The PAC1R-VIP simulation is based on a homology model and did not have the full Gs modelled, only the H5 was retained.

MD H-bonds (disulphide bond in ECD-ECL1 absent)

VPAC1:VIP:Gs			VPAC1:PACAP27:Gs			PAC1:PACAP27:Gs			PAC1:VIP		
W294$^{5.36}$	H1	10.6				Y241$^{3.44}$	H1	17.2	E374^{ECL3}	H1	25.9
Q223$^{3.37}$	H1	10.3				H234$^{3.37}$	H1	11.3			
E373$^{7.42}$	H1	7.4				E374^{ECL3}	H1	9.0			
K298$^{5.40}$	H1	5.8									
E373$^{7.42}$	S2	94.2	E373$^{7.42}$	S2	92.9	E385$^{7.42}$	S2	5.4	E385$^{7.42}$	S2	28.8
K369$^{7.38}$	S2	6.4	K369$^{7.38}$	S2	7.2						
R188$^{2.60}$	D3	43.0	R188$^{2.60}$	D3	73.8	R199$^{2.60}$	D3	92.4	Y157$^{1.43}$	D3	25.8
						Y161$^{1.47}$	D3	56.3	R199$^{2.60}$	D3	17.8
									Y161$^{1.47}$	D3	6.7
K195$^{2.67}$	T7	49.8	K195$^{2.67}$	T7	48.1	K206$^{2.67}$	T7	54.7	K206$^{2.67}$	T7	32.8
T288^{ECL2}	D8	37.4	T288^{ECL2}	D8	31.1	N300^{ECL2}	D8	6.1			
Y139$^{1.36}$	N9	22.9	Y139$^{1.36}$	S9	50.1	Y150$^{1.36}$	S9	48.2	Y150$^{1.36}$	N9	20.4
N363^{ECL3}	N9	7.8				K378$^{7.35}$	S9	20.0	D87^{ECD}	N9	8.3
									D147$^{1.33}$	Y10	18.8
D287^{ECL2}	T11	5.0	D287^{ECL2}	S11	82.5	D298^{ECL2}	S11	65.8	Y211^{ECL1}	T11	6.5
			Q207^{ECL1}	S11	4.2	Y211^{ECL1}	S11	32.5			
E36^{ECD}	R12	14.9	E36^{ECD}	R12	61.5	D301^{ECL2}	R12	59.7	D87^{ECD}	R12	53.2
T288^{ECL2}	R12	10.2				D87^{ECD}	R12	12.9			
N290^{ECL2}	R12	7.4									
			T136$^{1.33}$	Y13	50.6	D147$^{1.33}$	Y13	86.6			
			D132$^{1.29}$	Y13	21.4	T143$^{1.29}$	Y13	6.0			
E204^{ECL1}	R14	30.0	E204^{ECL1}	R14	49.3	D215^{ECL1}	R14	97.6	D215^{ECL1}	R14	72.3
Q207^{ECL1}	R14	9.1				Q214^{ECL1}	R14	14.2	D147$^{1.33}$	R14	22.9
E36^{ECD}	K15	13.4	D287^{ECL2}	K15	44.2	D298^{ECL2}	K15	38.0	D24^{ECD}	K15	16.4
D206^{ECL1}	K15	6.5	Q207^{ECL1}	K15	28.5						
D38^{ECD}	K15	5.8	E36^{ECD}	K15	11.0						
Y39^{ECD}	Q16	22.3	E36^{ECD}	Q16	15.9	N85^{ECD}	Q16	9.9	T143$^{1.29}$	Q16	15.4
D132$^{1.29}$	Q16	10.7							Y139$^{1.25}$	Q16	5.6
D132$^{1.29}$	K20	29.3	D132$^{1.29}$	K20	69.5	D137^{ECD}	K20	36.2	E138^{ECD}	K20	51.7
E133$^{1.30}$	K20	19.7				E142$^{1.28}$	K20	7.6	T143$^{1.29}$	K20	9.8
D126^{ECD}	K20	15.3									
D125^{ECD}	K20	9.5									
E204^{ECL1}	K21	48.2	E204^{ECL1}	K21	32.4						
E44^{ECD}	Y22	28.5	E44^{ECD}	Y22	44.1	N60^{ECD}	Y22	5.6			
			Q41^{ECD}	Y22	7.9						
Y118^{ECD}	N24	17.9									
			E114^{ECD}	L27	4.3						

MD H-bonds (disulphide bond in ECD-ECL1 present)

VPAC1:VIP:Gs			VPAC1:PACAP27:Gs			PAC1:PACAP27:Gs			PAC1:VIP		
E373$^{7.42}$	H1	11.1				Y241$^{3.44}$	H1	33.9	E374^{ECL3}	H1	20.2
D362^{ECL3}	H1	7.8							Y241$^{3.44}$	H1	15.3
E373$^{7.42}$	S2	85.5	E373$^{7.42}$	S2	94.4	E385$^{7.42}$	S2	34.3	E385$^{7.42}$	S2	22.9
			K369$^{7.38}$	S2	17.1						
R188$^{2.60}$	D3	43.1	R188$^{2.60}$	D3	79.7	R199$^{2.60}$	D3	92.0	R199$^{2.60}$	D3	90.1
Y146$^{1.43}$	D3	13.6				Y161$^{1.47}$	D3	63.8	Y161$^{1.47}$	D3	57.8
						Y241$^{3.44}$	D3	5.4	Y157$^{1.43}$	D3	19.2
K195$^{2.67}$	T7	43.0	K195$^{2.67}$	T7	47.3	K206$^{2.67}$	T7	61.9	K206$^{2.67}$	T7	48.2
T288^{ECL2}	D8	35.4	T288^{ECL2}	D8	58.5	N300^{ECL2}	D8	28.6			
						W306$^{5.36}$	D8	7.8			
Y139$^{1.36}$	N9	26.8	Y139$^{1.36}$	S9	41.9	Y150$^{1.36}$	S9	46.1	N375^{ECL3}	N9	17.8
N363^{ECL3}	N9	9.2	D201^{ECL1}	Y10	6.5	K378$^{7.35}$	S9	6.7	Y150$^{1.36}$	N9	16.9
									D147$^{1.33}$	Y10	10.5
D287^{ECL2}	T11	33.4	D287^{ECL2}	S11	90.4	D298^{ECL2}	S11	21.2	Y211^{ECL1}	T11	55.1
						Y211^{ECL1}	S11	12.5			
E36^{ECD}	R12	34.2	N290$^{5.32}$	R12	8.1	D301^{ECL2}	R12	18.2	D87^{ECD}	R12	64.9
			T288^{ECL2}	R12	7.8				D301^{ECL2}	R12	7.3
			T136$^{1.33}$	Y13	44.9	D147$^{1.33}$	Y13	78.9			
			D132$^{1.29}$	Y13	21.7						
E204^{ECL1}	R14	59.7	E210^{ECL1}	R14	29.1	D215^{ECL1}	R14	33.0	D215^{ECL1}	R14	67.2
Q207^{ECL1}	R14	32.6	E204^{ECL1}	R14	15.5	E213^{ECL1}	R14	26.6	D147$^{1.33}$	R14	53.6
			S205^{ECL1}	R14	13.3	D147$^{1.33}$	R14	15.1			
			D201^{ECL1}	R14	6.7	D298^{ECL2}	R14	15.0			
D287^{ECL2}	K15	63.8	E36^{ECD}	K15	34.2	D298^{ECL2}	K15	53.3	D298^{ECL2}	K15	77.1
E36^{ECD}	K15	59.3	E210^{ECL1}	K15	15.3						
			D287^{ECL2}	K15	14.9						
			Y283^{ECL2}	K15	8.0						
			E36^{ECD}	Q16	15.6				N85^{ECD}	Q16	13.2
									S141$^{1.27}$	Q16	5.1
D132$^{1.29}$	K20	35.7	D132$^{1.29}$	K20	63.7	D132^{ECD}	K20	6.5	E138^{ECD}	K20	27.3
D126^{ECD}	K20	14.4							E140$^{1.26}$	K20	25.6
E133$^{1.30}$	K20	7.8							T143$^{1.29}$	K20	14.2
									D137^{ECD}	K20	8.1
									E142$^{1.28}$	K20	5.2
E204^{ECL1}	K21	35.7	E204^{ECL1}	K21	41.0	E213^{ECL1}	K21	15.9	D215^{ECL1}	K21	27.4
D206^{ECL1}	K21	6.7	D206^{ECL1}	K21	38.0	D215^{ECL1}	K21	13.8	H218^{ECL1}	K21	6.0
S205^{ECL1}	K21	6.3				E142$^{1.28}$	K21	5.1			
E44^{ECD}	Y22	15.4	E44^{ECD}	Y22	42.0				Q31^{ECD}	Y22	10.6
			Q41^{ECD}	Y22	5.0						
									D137^{ECD}	N24	10.2
									K28^{ECD}	S25	12.5

Legend: TM1, TM2, TM3, TM4, TM5, TM6, TM7, ECD, ECL1, ECL2, ECL3; Min % – Max %

Supplementary Table 5: Water bridges between receptor and peptide residues during MD equilibrium simulations. Columns are sorted by peptide residues and highest to lowest occupancy. Contacts are shown as occupancy of MD frames (in percent), and background is coloured on a scale with highest occupancy in red and lowest occupancy in white. Receptor residues are labelled according to the Wootten numbering system and background is coloured by residue position (TMs, ECLs and ECD, see legend). The PAC1R-VIP simulation is based on a homology model and did not have the full Gs modelled, only the H5 was retained. The ECD-ECL1 disulphide bond was present for the PAC1R-VIP simulation (right column), and absent for all other simulations. (b) indicates interactions with the backbone.

Water bridge occupancy (% MD Frames)										
	VPAC1R-VIP		VPAC1R-PACAP27		PAC1R-PACAP27		PAC1R-VIP		PAC1R-VIP + disulf	
**His1**	R188$^{2.60}$	30	Q223$^{3.37}$	46.1	E374^{ECL3}	39.8	E374^{ECL3}	66.5	E374^{ECL3}	58.1
	E373$^{7.42}$	29.9	D362^{ECL3}	18.2	H234$^{3.37}$	25.2	E385$^{7.42}$	27.7	E385$^{7.42}$	46.7
	D362^{ECL3}	27.6	E373$^{7.42}$	15.9	E385$^{7.42}$	21.1	Y241$^{3.44}$	13.8	Y241$^{3.44}$	31.2
	Q223$^{3.37}$	20.4	Y354$^{6.53}$	11.7	R199$^{2.60}$	18.7	F369$^{6.56}$	10.5	R381$^{7.38}$	16.1
	Y354$^{6.53}$	15.9	R188$^{2.60}$	9	W306$^{5.36}$	18.7	H234$^{3.37}$	9.4	H234$^{3.37}$	11.7
	K369$^{7.38}$	9.6	K369$^{7.38}$	8.4	Y241$^{3.44}$	13.9	S372$^{6.59}$	6.9	F369$^{6.56}$	11.5
	F357$^{6.56}$	6.2	F357$^{6.56}$	5.2	N300^{ECL2}	6.4	Y366$^{6.53}$	6.9	Y366$^{6.53}$	11
					I309$^{5.39}$ (b)	5.1	I309$^{5.39}$ (b)	6.6	R199$^{2.60}$	6.3
							R381$^{7.38}$	6.5		
							W306$^{5.36}$	6.4		
							N300^{ECL2}	5.7		
							R199$^{2.60}$	4.2		
**Ser2**	E373$^{7.42}$	46.3	E373$^{7.42}$	21.5	E385$^{7.42}$	74.8	E385$^{7.42}$	78.1	E385$^{7.42}$	69.2
	D362^{ECL3}	9.1	K369$^{7.38}$	13.8	Y241$^{3.44}$	25.1			R199$^{2.60}$	16.9
	K369$^{7.38}$	8.4	D362^{ECL3}	6.2	R199$^{2.60}$	25.1			Y241$^{3.44}$	12.1
					Y366$^{6.53}$	6.9			Y161$^{1.47}$	10.2
									Y366$^{6.53}$	5.8
**Asp3**	E373$^{7.42}$	70.5	R188$^{2.60}$	92.1	R199$^{2.60}$	62.7	Y161$^{1.47}$	61.1	R199$^{2.60}$	59.4
	R188$^{2.60}$	66.1	E373$^{7.42}$	65.2	Y161$^{1.47}$	53	R199$^{2.60}$	53	Y161$^{1.47}$	56.2
	Y150$^{1.47}$	62.4	Y150$^{1.47}$	55.3	K206$^{2.67}$	22.1	K206$^{2.67}$	35.1	L386$^{7.43}$ (b)	17.7
	K195$^{2.67}$	26.9	K195$^{2.67}$	9.4	L386$^{7.43}$ (b)	21.1	Y157$^{1.43}$	20.7	Y241$^{3.44}$	16.1
	Y146$^{1.43}$	7.9	L374$^{7.43}$	8.2	Y241$^{3.44}$	18.3	E385$^{7.42}$	10.1	Y157$^{1.43}$	7.7
			Y354$^{6.53}$	5.8	S390$^{7.47}$	6.8				

TM1
TM2
TM3
TM4
TM5
TM6
TM7
ECD
ECL1
ECL2
ECL3

Min %
Max %

Supplementary Table 6: Non-equilibrium MD simulation details. Summary of the non-equilibrium MD simulations performed.

Complex	Unbinding (well-tempered MetaD) # Replicas	SuMD Binding # Replicas	Analyzed Unbinding/Binding Total Path Sampling
**PAC1R:VIP:Gs(H5)**	4	12	3.04 μs / 3.10 μs
**PAC1R:PACAP27:Gs(H5)**	4	12	3.04 μs / 2.64 μs
**VPAC1R:VIP:Gs(H5)**	4	12	3.12 μs / 2.46 μs
**VPAC1R:PACAP27:Gs(H5)**	4	12	3.01 μs / 2.34 μs

Supplementary Table 7: Contacts between receptor and peptide residues during MD partial binding and unbinding simulations. Columns are sorted by peptide residues and highest to lowest occupancy. Contacts are shown as occupancy of MD frames (in percent), and background is coloured on a scale with highest occupancy in red and lowest occupancy in white. Receptor residues are labelled according to the Wootten numbering system and background is coloured by residue position (TMs, ECLs and ECD, see legend).

Contacts Partial Binding

PACAP27-PAC1R

H1	D301^{ECL2}	10.0
H1	M299^{ECL2}	7.3
H1	T294^{ECL2}	4.9
H1	N375^{ECL3}	4.3
H1	D292$^{4.68}$	4.3
H1	D293^{ECL2}	3.5
H1	E374^{ECL3}	3.5
H1	K378$^{7.35}$	3.2
S2	M299^{ECL2}	6.3
S2	D301^{ECL2}	5.8
S2	N375^{ECL3}	2.6
D3	K378$^{7.35}$	10.7
D3	T294^{ECL2}	4.7
D3	N375^{ECL3}	4.4
D3	M299^{ECL2}	3.0
D3	D293^{ECL2}	2.2
G4	M299^{ECL2}	4.7
I5	R379$^{7.36}$	6.3
I5	K378$^{7.35}$	4.8
I5	L382$^{7.39}$	4.4
I5	N375^{ECL3}	2.8
I5	M299^{ECL2}	2.4

PACAP27-VPAC1R

S2	N363^{ECL3}	6.2
D3	N363^{ECL3}	8.6
D3	N290$^{5.32}$	6.7
G4	N363^{ECL3}	9.4
I5	N363^{ECL3}	7.2
I5	C208^{ECL1}	4.8
I5	I289^{ECL2}	4.7

VIP-VPAC1R

H1	D362^{ECL3}	10.8
H1	E367$^{7.36}$	7.3
H1	P366$^{7.35}$	6.6
H1	K365$^{7.34}$	6.5
H1	F280$^{4.67}$	6.5
H1	S292$^{5.34}$	4.3
H1	E281$^{4.68}$	3.8
H1	P361^{ECL3}	3.2
H1	Q135$^{1.32}$	3.1
S2	F280$^{4.67}$	6.8
S2	P366$^{7.35}$	5.4
D3	K365$^{7.34}$	14.4
D3	K369$^{7.38}$	7.6
D3	F280$^{4.67}$	4.9
D3	Y283^{ECL2}	4.7
D3	M370$^{7.39}$	4.5
D3	D282^{ECL2}	3.1
D3	P366$^{7.35}$	3.0
A4	W294$^{5.36}$	3.6
V5	Y139$^{1.36}$	3.0

VIP-PAC1R

H1	M299^{ECL2}	8.2
H1	N375^{ECL3}	7.3
H1	D298^{ECL2}	7.1
H1	E374^{ECL3}	6.5
H1	D147$^{1.33}$	5.4
H1	N300^{ECL2}	5.1
H1	W306$^{5.36}$	4.5
H1	D215^{ECL1}	4.3
H1	E91^{ECD}	4.2
H1	W90^{ECD}	3.8
H1	F220^{ECL1}	3.4
H1	E385$^{7.42}$	3.0
H1	H218^{ECL1}	2.4
H1	C219^{ECL1}	2.1
H1	Y150$^{1.36}$	2.0
S2	N375^{ECL3}	5.0
S2	E91^{ECD}	4.1
S2	E374^{ECL3}	3.6
S2	D147$^{1.33}$	2.6
S2	N300^{ECL2}	2.5
S2	K378$^{7.35}$	2.3
D3	W306$^{5.36}$	6.5
D3	K310$^{5.40}$	6.2
D3	K378$^{7.35}$	2.5
D3	K206$^{2.67}$	2.2
A4	K378$^{7.35}$	2.8
A4	M299^{ECL2}	2.6
A4	N300^{ECL2}	2.5
A4	R379$^{7.36}$	2.1
V5	W90^{ECD}	7.3
V5	S141$^{1.27}$	4.7
V5	N375^{ECL3}	3.8
V5	L382$^{7.39}$	3.7
V5	E91^{ECD}	3.3
V5	K378$^{7.35}$	3.3
V5	Y150$^{1.36}$	2.5
V5	R379$^{7.36}$	2.3

Contacts Partial Unbinding

PACAP27-PAC1R

H1	E374^{ECL3}	22.6
H1	W306$^{5.36}$	22.4
H1	N300^{ECL2}	16.1
H1	E385$^{7.42}$	12.3
H1	M299^{ECL2}	8.0
H1	D301^{ECL2}	7.3
H1	N375^{ECL3}	6.3
H1	V237$^{3.40}$	5.8
H1	V313$^{5.43}$	5.6
H1	T303$^{5.33}$	4.5
H1	Y241$^{3.44}$	4.5
H1	K310$^{5.40}$	3.0
H1	R381$^{7.38}$	2.6
H1	D298^{ECL2}	2.1
S2	L382$^{7.39}$	17.7
S2	E385$^{7.42}$	13.5
S2	L386$^{7.43}$	10.8
S2	K378$^{7.35}$	8.4
S2	E374^{ECL3}	8.3
S2	N375^{ECL3}	6.8
S2	R199$^{2.60}$	3.3
S2	R381$^{7.38}$	3.0
D3	K378$^{7.35}$	13.5
D3	R199$^{2.60}$	10.5
D3	F233$^{3.36}$	8.8
D3	Y161$^{1.47}$	7.5
D3	M299^{ECL2}	7.1
D3	L386$^{7.43}$	5.5
D3	K206$^{2.67}$	4.1
D3	Y211^{ECL1}	3.0
D3	D301^{ECL2}	2.4
D3	L382$^{7.39}$	2.3
D3	N375^{ECL3}	2.1
G4	M299^{ECL2}	21.2
G4	D298^{ECL2}	5.0
G4	D301^{ECL2}	3.2
G4	N300^{ECL2}	2.8
G4	W306$^{5.36}$	2.4
I5	L382$^{7.39}$	7.9
I5	Y150$^{1.36}$	4.2
I5	K378$^{7.35}$	3.4
I5	E374^{ECL3}	3.2
I5	N375^{ECL3}	3.1
I5	M299^{ECL2}	2.8
I5	W306$^{5.36}$	2.1

PACAP27-VPAC1R

H1	E373$^{7.42}$	21.8
H1	D362^{ECL3}	19.2
H1	W294$^{5.36}$	10.9
H1	E367$^{7.36}$	10.4
H1	K369$^{7.38}$	9.5
H1	K298$^{5.40}$	8.9
H1	I289^{ECL2}	7.7
H1	P366$^{7.35}$	5.4
H1	I301$^{5.43}$	5.0
H1	N363^{ECL3}	3.9
H1	N290$^{5.32}$	3.5
S2	E373$^{7.42}$	21.3
S2	M370$^{7.39}$	17.3
S2	K369$^{7.38}$	13.9
S2	E367$^{7.36}$	7.2
S2	Y139$^{1.36}$	6.9
S2	Q135$^{1.32}$	4.4
S2	P366$^{7.35}$	4.0
S2	L374$^{7.43}$	3.9
D3	L374$^{7.43}$	6.4
D3	Y139$^{1.36}$	5.0
D3	N290$^{5.32}$	3.8
D3	I289^{ECL2}	3.7
D3	M370$^{7.39}$	3.7
D3	P366$^{7.35}$	3.3
G4	I289^{ECL2}	11.0
G4	N290$^{5.32}$	4.5
I5	Q135$^{1.32}$	6.2
I5	D362^{ECL3}	5.5
I5	C208^{ECL1}	4.7
I5	M370$^{7.39}$	4.5
I5	Y283^{ECL2}	4.5
I5	L92^{ECD}	4.3
I5	I289^{ECL2}	4.2
I5	N363^{ECL3}	4.0

VIP-VPAC1R

H1	E373$^{7.42}$	12.5
H1	W294$^{5.36}$	12.3
H1	K298$^{5.40}$	8.1
H1	P366$^{7.35}$	7.0
H1	K365$^{7.34}$	5.9
H1	E367$^{7.36}$	5.8
H1	K369$^{7.38}$	5.4
H1	I289^{ECL2}	4.8
H1	I301$^{5.43}$	4.4
H1	Q135$^{1.32}$	4.3
H1	Y139$^{1.36}$	3.9
H1	D362^{ECL3}	3.7
H1	V226$^{3.40}$	3.1
H1	Q223$^{3.37}$	2.6
H1	Y354$^{6.53}$	2.5
S2	M370$^{7.39}$	26.0
S2	E373$^{7.42}$	21.1
S2	K369$^{7.38}$	10.4
S2	P366$^{7.35}$	7.2
S2	Y139$^{1.36}$	6.5
S2	K365$^{7.34}$	5.4
S2	L374$^{7.43}$	2.6
D3	L374$^{7.43}$	8.6
D3	Y139$^{1.36}$	8.5
D3	M370$^{7.39}$	8.2
D3	F222$^{3.36}$	7.4
D3	R188$^{2.60}$	5.3
D3	I289^{ECL2}	3.8
D3	P366$^{7.35}$	2.8
A4	I289^{ECL2}	22.9
A4	T288^{ECL2}	6.3
A4	N290$^{5.32}$	4.9
A4	W294$^{5.36}$	4.5
V5	I289^{ECL2}	7.6
V5	M370$^{7.39}$	4.7

VIP-PAC1R

H1	E374^{ECL3}	25.9
H1	W306$^{5.36}$	16.7
H1	N300^{ECL2}	9.5
H1	D215^{ECL1}	8.9
H1	M299^{ECL2}	8.6
H1	D298^{ECL2}	7.1
H1	Y211^{ECL1}	6.8
H1	V237$^{3.40}$	6.3
H1	V313$^{5.43}$	5.5
H1	K310$^{5.40}$	5.4
H1	E385$^{7.42}$	5.0
H1	H218^{ECL1}	4.6
H1	N375^{ECL3}	4.1
H1	F220^{ECL1}	2.7
H1	K378$^{7.35}$	2.4
H1	L382$^{7.39}$	2.3
H1	C219^{ECL1}	2.1
S2	L382$^{7.39}$	24.5
S2	E385$^{7.42}$	21.5
S2	L386$^{7.43}$	15.0
S2	E374^{ECL3}	5.5
S2	D215^{ECL1}	2.9
S2	K378$^{7.35}$	2.9
S2	Y150$^{1.36}$	2.6
S2	H218^{ECL1}	2.2
D3	K206$^{2.67}$	10.5
D3	L386$^{7.43}$	9.7
D3	R199$^{2.60}$	6.7
D3	Y211^{ECL1}	6.7
D3	F233$^{3.36}$	6.6
D3	K154$^{1.40}$	6.4
D3	Y161$^{1.47}$	3.3
D3	Y157$^{1.43}$	2.2
A4	M299^{ECL2}	11.0
A4	W306$^{5.36}$	6.1
A4	N300^{ECL2}	5.1
A4	Y211^{ECL1}	2.4
V5	L382$^{7.39}$	13.1
V5	K378$^{7.35}$	6.5
V5	E374^{ECL3}	6.1
V5	N375^{ECL3}	3.8
V5	W306$^{5.36}$	2.6
V5	R379$^{7.36}$	2.3

TM1
TM2
TM3
TM4
TM5
TM6
TM7
ECD
ECL1
ECL2
ECL3

Min %

Max %

Supplementary Table 7 continued

Contacts Partial Binding

PACAP27-PAC1R		
F6	Y150$^{1.36}$	17.3
F6	L382$^{7.39}$	11.6
F6	R379$^{7.36}$	11.1
F6	F220^{ECL1}	6.4
F6	T294^{ECL2}	4.8
F6	V383$^{7.40}$	4.5
F6	Y149$^{1.35}$	4.0
F6	K378$^{7.35}$	3.6
T7	F220^{ECL1}	10.2
T7	K378$^{7.35}$	4.0
T7	C219^{ECL1}	3.7
T7	M299^{ECL2}	3.5
T7	Y150$^{1.36}$	3.1
T7	H218^{ECL1}	2.7
T7	T294^{ECL2}	2.5
T7	L382$^{7.39}$	2.4
S9	D147$^{1.33}$	9.9
S9	Y150$^{1.36}$	9.4
S9	Q146$^{1.32}$	3.2
Y10	Y150$^{1.36}$	15.9
Y10	F220^{ECL1}	15.1
Y10	D215^{ECL1}	9.8
Y10	H218^{ECL1}	7.7
Y10	Q214^{ECL1}	5.1
Y10	C219^{ECL1}	4.1
Y10	Y211^{ECL1}	4.0
Y10	D147$^{1.33}$	2.2
S11	H218^{ECL1}	12.6
S11	F220^{ECL1}	9.3
S11	N217^{ECL1}	8.0
S11	C219^{ECL1}	4.5

PACAP27-VPAC1R		
F6	Y139$^{1.36}$	13.4
F6	T136$^{1.33}$	8.7
F6	P366$^{7.35}$	8.1
F6	M370$^{7.39}$	7.9
F6	Q135$^{1.32}$	7.6
F6	N363^{ECL3}	7.3
F6	D132$^{1.29}$	6.3
T7	C208^{ECL1}	6.7
T7	Y139$^{1.36}$	6.5
T7	N290$^{5.32}$	4.9
S9	D132$^{1.29}$	12.1
S9	Y139$^{1.36}$	8.2
S9	Q135$^{1.32}$	5.3
Y10	T136$^{1.33}$	18.5
Y10	Y139$^{1.36}$	11.9
Y10	E36^{ECD}	9.6
Y10	D132$^{1.29}$	7.9
Y10	E133$^{1.30}$	7.0
Y10	Q207^{ECL1}	6.8
Y10	L199$^{2.71}$	5.5
Y10	C208^{ECL1}	5.4
Y10	E204^{ECL1}	5.4
S11	E204^{ECL1}	5.4

VIP-VPAC1R		
F6	Y283^{ECL2}	19.9
F6	D282^{ECL2}	11.4
F6	F280$^{4.67}$	11.3
F6	E281$^{4.68}$	9.9
F6	Y139$^{1.36}$	9.3
F6	P366$^{7.35}$	8.8
F6	H279$^{4.66}$	8.3
F6	M370$^{7.39}$	8.0
F6	K365$^{7.34}$	4.4
F6	Q135$^{1.32}$	2.9
F6	W286^{ECL2}	2.7
T7	Y283^{ECL2}	6.2
T7	I289^{ECL2}	3.9
T7	M370$^{7.39}$	3.5
T7	C208^{ECL1}	3.4
T7	S209^{ECL1}	2.9
D8	T288^{ECL2}	2.7
N9	F93^{ECD}	17.4
N9	Y283^{ECL2}	10.4
N9	Y139$^{1.36}$	9.3
Y10	C208^{ECL1}	11.5
Y10	Y283^{ECL2}	11.2
Y10	Y139$^{1.36}$	9.2
Y10	D287^{ECL2}	7.7
Y10	K143$^{1.40}$	6.4
Y10	D206^{ECL1}	6.0
Y10	F200^{ECL1}	5.9
Y10	L374$^{7.43}$	4.4
Y10	M370$^{7.39}$	3.9
Y10	D282^{ECL2}	3.2
Y10	T288^{ECL2}	2.9
Y10	E210^{ECL1}	2.7
T11	C208^{ECL1}	5.6
T11	T288^{ECL2}	3.9
T11	D287^{ECL2}	3.3

VIP-PAC1R		
F6	N375^{ECL3}	4.6
F6	E374^{ECL3}	2.9
F6	K154$^{1.40}$	2.7
F6	Y150$^{1.36}$	2.4
T7	M299^{ECL2}	2.8
T7	L210$^{2.71}$	2.3
D8	R379$^{7.36}$	5.5
D8	K378$^{7.35}$	4.0
D8	Y139$^{1.25}$	3.1
D8	W90^{ECD}	2.7
D8	S141$^{1.27}$	2.2
N9	Y139$^{1.25}$	19.7
N9	S141$^{1.27}$	7.6
N9	P86^{ECD}	6.9
N9	N375^{ECL3}	5.2
N9	E140$^{1.26}$	3.5
N9	W90^{ECD}	3.2
N9	Y150$^{1.36}$	3.1
N9	R379$^{7.36}$	2.5
N9	Q146$^{1.32}$	2.3
N9	E142$^{1.28}$	2.0
Y10	D215^{ECL1}	5.6
Y10	Y211^{ECL1}	5.5
Y10	L210$^{2.71}$	2.2
Y10	D298^{ECL2}	2.0
T11	C219^{ECL1}	3.7
T11	F220^{ECL1}	3.0

Contacts Partial Unbinding

PACAP27-PAC1R		
F6	Y150$^{1.36}$	33.1
F6	V153$^{1.39}$	20.6
F6	Y157$^{1.43}$	13.1
F6	L382$^{7.39}$	11.7
F6	Y211^{ECL1}	6.7
F6	Y149$^{1.35}$	6.5
F6	L386$^{7.43}$	5.7
F6	Q146$^{1.32}$	4.3
F6	K154$^{1.40}$	3.5
F6	R379$^{7.36}$	3.3
T7	Y211^{ECL1}	17.3
T7	D298^{ECL2}	15.5
T7	M299^{ECL2}	15.5
T7	K206$^{2.67}$	10.6
T7	L210$^{2.71}$	5.1
T7	F233$^{3.36}$	3.7
T7	F220^{ECL1}	3.5
D8	M299^{ECL2}	16.0
D8	N300^{ECL2}	10.0
D8	D298^{ECL2}	5.7
S9	Y150$^{1.36}$	26.5
S9	E142$^{1.28}$	3.5
Y10	Y150$^{1.36}$	34.3
Y10	Y211^{ECL1}	29.7
Y10	D215^{ECL1}	8.7
Y10	K154$^{1.40}$	6.5
Y10	Q146$^{1.32}$	5.7
Y10	F220^{ECL1}	5.0
Y10	D147$^{1.33}$	4.1
Y10	T294^{ECL2}	2.6
Y10	M299^{ECL2}	2.3
Y10	G295^{ECL2}	2.1
S11	F220^{ECL1}	8.8
S11	D298^{ECL2}	8.4
S11	M299^{ECL2}	8.1
S11	Y211^{ECL1}	7.7

PACAP27-VPAC1R		
F6	Y139$^{1.36}$	32.4
F6	V142$^{1.39}$	18.3
F6	M370$^{7.39}$	16.2
F6	F200^{ECL1}	9.9
F6	L374$^{7.43}$	9.7
F6	Y146$^{1.43}$	8.1
F6	F138$^{1.35}$	5.3
F6	T136$^{1.33}$	5.1
F6	Q135$^{1.32}$	4.9
F6	L92^{ECD}	3.9
T7	F200^{ECL1}	15.7
T7	L199$^{2.71}$	9.4
T7	Y139$^{1.36}$	4.8
T7	D287^{ECL2}	4.5
D8	T288^{ECL2}	13.9
D8	I289^{ECL2}	10.8
D8	K127^{ECD}	6.3
D8	D287^{ECL2}	4.0
S9	Y139$^{1.36}$	22.2
S9	D132$^{1.29}$	9.8
Y10	F200^{ECL1}	32.2
Y10	Y139$^{1.36}$	31.3
Y10	K143$^{1.40}$	9.4
Y10	T136$^{1.33}$	8.6
Y10	C208^{ECL1}	6.5
Y10	D132$^{1.29}$	6.0
Y10	E204^{ECL1}	5.3
Y10	Q135$^{1.32}$	5.2
Y10	Q207^{ECL1}	4.5
Y10	S205^{ECL1}	4.5
Y10	L92^{ECD}	3.8
Y10	D201^{ECL1}	3.7
Y10	S130$^{1.27}$	3.6
S11	F200^{ECL1}	12.1
S11	D287^{ECL2}	10.6
S11	Q207^{ECL1}	5.2
S11	Y139$^{1.36}$	3.8

VIP-VPAC1R		
F6	Y139$^{1.36}$	56.9
F6	M370$^{7.39}$	20.8
F6	V142$^{1.39}$	19.0
F6	Y146$^{1.43}$	14.5
F6	F200^{ECL1}	11.8
F6	Q135$^{1.32}$	11.3
F6	L374$^{7.43}$	9.9
F6	T136$^{1.33}$	8.9
F6	L131$^{1.28}$	6.3
F6	D132$^{1.29}$	4.5
F6	A128^{ECD}	3.2
F6	F138$^{1.35}$	3.0
F6	K127^{ECD}	2.8
F6	P366$^{7.35}$	2.8
T7	D287^{ECL2}	24.8
T7	F200^{ECL1}	20.4
T7	L199$^{2.71}$	16.8
T7	K195$^{2.67}$	6.7
T7	I289^{ECL2}	6.2
T7	Y139$^{1.36}$	4.0
T7	T288^{ECL2}	3.9
D8	I289^{ECL2}	17.2
D8	T288^{ECL2}	17.0
D8	D287^{ECL2}	6.6
N9	Y139$^{1.36}$	29.7
N9	F93^{ECD}	11.1
N9	D132$^{1.29}$	7.1
N9	M370$^{7.39}$	2.8
Y10	F200^{ECL1}	56.8
Y10	Y139$^{1.36}$	39.3
Y10	K143$^{1.40}$	10.2
Y10	T136$^{1.33}$	8.2
Y10	G140$^{1.37}$	7.3
Y10	L131$^{1.28}$	4.9
Y10	Q135$^{1.32}$	4.3
Y10	D132$^{1.29}$	3.7
Y10	A128^{ECD}	3.2
Y10	Q207^{ECL1}	3.0
Y10	D287^{ECL2}	2.7
T11	D287^{ECL2}	30.4
T11	F200^{ECL1}	16.1
T11	L199$^{2.71}$	4.0
T11	Q207^{ECL1}	3.6

VIP-PAC1R		
F6	Y150$^{1.36}$	41.4
F6	V153$^{1.39}$	20.8
F6	L386$^{7.43}$	18.2
F6	Y157$^{1.43}$	15.2
F6	L382$^{7.39}$	13.6
F6	K154$^{1.40}$	8.0
F6	Y211^{ECL1}	5.0
F6	T143$^{1.29}$	4.5
F6	Q146$^{1.32}$	3.6
F6	R379$^{7.36}$	2.9
F6	D147$^{1.33}$	2.6
F6	K378$^{7.35}$	2.6
T7	Y211^{ECL1}	17.8
T7	K206$^{2.67}$	6.7
T7	M299^{ECL2}	6.4
T7	L210$^{2.71}$	5.8
T7	D298^{ECL2}	4.4
D8	M299^{ECL2}	8.5
D8	N300^{ECL2}	4.2
N9	Y150$^{1.36}$	35.3
N9	P86^{ECD}	9.0
N9	K378$^{7.35}$	8.9
N9	R379$^{7.36}$	5.5
N9	S141$^{1.27}$	5.4
N9	T143$^{1.29}$	4.1
N9	Q146$^{1.32}$	4.0
N9	L382$^{7.39}$	3.8
N9	D87^{ECD}	2.2
N9	Y139$^{1.25}$	2.2
Y10	Y150$^{1.36}$	30.5
Y10	D147$^{1.33}$	23.0
Y10	Y211^{ECL1}	22.1
Y10	Q146$^{1.32}$	10.0
Y10	T143$^{1.29}$	8.6
Y10	K154$^{1.40}$	7.9
Y10	L151$^{1.37}$	5.7
T11	Y211^{ECL1}	7.9
T11	M299^{ECL2}	3.7
T11	D298^{ECL2}	2.7

Supplementary Table 7 continued

Contacts Partial Binding

PACAP27-PAC1R			PACAP27-VPAC1R			VIP-VPAC1R			VIP-PAC1R		
R12	D87^{ECD}	36.8	R12	E36^{ECD}	41.2	R12	F93^{ECD}	46.6	R12	D87^{ECD}	53.8
R12	D147$^{1.33}$	9.4	R12	D132$^{1.29}$	17.9	R12	E36^{ECD}	23.8	R12	I83^{ECD}	51.9
R12	N85^{ECD}	4.9	R12	F200^{ECL1}	8.3	R12	Y39^{ECD}	15.8	R12	R82^{ECD}	27.3
R12	I83^{ECD}	2.6	R12	D287^{ECL2}	6.0	R12	L92^{ECD}	8.2	R12	N85^{ECD}	26.8
R12	P86^{ECD}	2.6				R12	D132$^{1.29}$	7.6	R12	Y139$^{1.25}$	25.0
						R12	I89^{ECD}	2.9	R12	P86^{ECD}	10.6
						R12	T136$^{1.33}$	2.6	R12	D147$^{1.33}$	7.2
									R12	Q146$^{1.32}$	4.0
									R12	F84^{ECD}	3.8
									R12	E140$^{1.26}$	3.3
									R12	W90^{ECD}	2.7
									R12	S141$^{1.27}$	2.3
									R12	D110^{ECD}	2.0
									R12	E142$^{1.28}$	2.0
Y13	Y139$^{1.25}$	24.1	Y13	T136$^{1.33}$	18.8	L13	F93^{ECD}	16.7	L13	Y139$^{1.25}$	12.7
Y13	D87^{ECD}	17.0	Y13	Y139$^{1.36}$	14.3	L13	Y283^{ECL2}	8.7	L13	E140$^{1.26}$	4.1
Y13	P86^{ECD}	15.4	Y13	E133$^{1.30}$	14.2	L13	C208^{ECL1}	4.2	L13	E142$^{1.28}$	3.9
Y13	Y150$^{1.36}$	8.6	Y13	S130$^{1.27}$	10.9				L13	K378$^{7.35}$	3.9
Y13	D147$^{1.33}$	8.5	Y13	D132$^{1.29}$	8.2				L13	Y150$^{1.36}$	3.3
Y13	L151$^{1.37}$	7.8	Y13	F200^{ECL1}	7.6				L13	S141$^{1.27}$	2.7
Y13	S141$^{1.27}$	5.0							L13	T143$^{1.29}$	2.7
Y13	Q146$^{1.32}$	2.8									
Y13	E140$^{1.26}$	2.8									
R14	H218^{ECL1}	33.3	R14	E204^{ECL1}	41.9	R14	D287^{ECL2}	23.4	R14	D215^{ECL1}	9.7
R14	D215^{ECL1}	24.3	R14	S202^{ECL1}	14.6	R14	E204^{ECL1}	14.7	R14	Y150$^{1.36}$	4.5
R14	D24^{ECD}	23.4	R14	L199$^{2.71}$	13.6	R14	E210^{ECL1}	12.2	R14	H218^{ECL1}	4.4
R14	E142$^{1.28}$	20.1	R14	Q207^{ECL1}	12.9	R14	S202^{ECL1}	9.5	R14	C219^{ECL1}	4.2
R14	Q214^{ECL1}	15.7	R14	E133$^{1.30}$	9.0	R14	G203^{ECL1}	8.8	R14	D147$^{1.33}$	2.8
R14	N217^{ECL1}	8.4	R14	G203^{ECL1}	7.5	R14	L199$^{2.71}$	8.4			
R14	C219^{ECL1}	6.6	R14	F200^{ECL1}	7.4	R14	C208^{ECL1}	7.3			
R14	F220^{ECL1}	6.5	R14	D287^{ECL2}	7.2	R14	D206^{ECL1}	6.3			
R14	D147$^{1.33}$	5.1				R14	D196$^{2.68}$	4.9			
R14	E140$^{1.26}$	4.9				R14	F200^{ECL1}	4.5			
R14	Y211^{ECL1}	4.7				R14	E36^{ECD}	3.0			
R14	S216^{ECL1}	3.8				R14	S209^{ECL1}	3.0			
R14	E213^{ECL1}	2.4				R14	S205^{ECL1}	2.7			
K15	D24^{ECD}	11.9	K15	E36^{ECD}	61.2	K15	D38^{ECD}	54.1	K15	I83^{ECD}	50.9
K15	H218^{ECL1}	11.0	K15	C37^{ECD}	15.4	K15	F93^{ECD}	31.4	K15	D24^{ECD}	6.7
K15	N217^{ECL1}	10.3	K15	C208^{ECL1}	7.0	K15	Y39^{ECD}	28.8	K15	H218^{ECL1}	4.1
K15	S216^{ECL1}	7.1	K15	E204^{ECL1}	6.5	K15	E36^{ECD}	21.7	K15	L80^{ECD}	4.0
K15	D215^{ECL1}	5.6	K15	Q207^{ECL1}	6.3	K15	D287^{ECL2}	7.7	K15	E79^{ECD}	3.9
K15	C219^{ECL1}	5.0	K15	E210^{ECL1}	6.1	K15	C37^{ECD}	7.2	K15	D215^{ECL1}	3.5
K15	I83^{ECD}	3.0				K15	I89^{ECD}	3.2	K15	F84^{ECD}	3.1
K15	Q214^{ECL1}	2.6				K15	V40^{ECD}	3.2	K15	C25^{ECD}	2.9
K15	F220^{ECL1}	2.3							K15	F220^{ECL1}	2.1
Q16	F84^{ECD}	51.9	Q16	F93^{ECD}	49.6	Q16	F93^{ECD}	51.3	Q16	F84^{ECD}	68.2
Q16	I83^{ECD}	27.9	Q16	E36^{ECD}	36.4	Q16	L92^{ECD}	35.7	Q16	I83^{ECD}	60.6
Q16	Y139$^{1.25}$	24.1	Q16	L92^{ECD}	14.5	Q16	I89^{ECD}	31.5	Q16	Y139$^{1.25}$	34.8
Q16	N85^{ECD}	7.1	Q16	T136$^{1.33}$	10.1	Q16	Y118^{ECD}	15.0	Q16	D137^{ECD}	15.7
Q16	P86^{ECD}	6.0	Q16	D132$^{1.29}$	9.5	Q16	Y39^{ECD}	10.3	Q16	E138^{ECD}	12.7
Q16	F136^{ECD}	2.2	Q16	E133$^{1.30}$	6.2	Q16	F90^{ECD}	8.1	Q16	S141$^{1.27}$	6.0
						Q16	P117^{ECD}	7.3	Q16	E140$^{1.26}$	5.5
						Q16	S94^{ECD}	5.5	Q16	F136^{ECD}	5.5
									Q16	E142$^{1.28}$	4.3

Contacts Partial Unbinding

PACAP27-PAC1R			PACAP27-VPAC1R			VIP-VPAC1R			VIP-PAC1R		
R12	I83^{ECD}	46.6	R12	E36^{ECD}	14.6	R12	F93^{ECD}	51.4	R12	D87^{ECD}	65.2
R12	D87^{ECD}	28.7	R12	D132$^{1.29}$	14.1	R12	E36^{ECD}	31.1	R12	I83^{ECD}	46.1
R12	N85^{ECD}	23.1	R12	D287^{ECL2}	12.7	R12	D287^{ECL2}	5.7	R12	N85^{ECD}	30.1
R12	M299^{ECL2}	9.5	R12	K127^{ECD}	9.6	R12	T288^{ECL2}	4.6	R12	R82^{ECD}	14.4
R12	D301^{ECL2}	9.2	R12	L92^{ECD}	8.6	R12	D132$^{1.29}$	3.5	R12	P86^{ECD}	13.4
R12	E142$^{1.28}$	5.2	R12	F200^{ECL1}	8.0	R12	Y39^{ECD}	3.3	R12	Y139$^{1.25}$	7.6
R12	P86^{ECD}	3.7	R12	T288^{ECL2}	7.7				R12	M299^{ECL2}	4.6
R12	Y139$^{1.25}$	2.2	R12	F93^{ECD}	7.1						
			R12	L199$^{2.71}$	4.6						
			R12	I289^{ECL2}	3.7						
Y13	D147$^{1.33}$	31.9	Y13	T136$^{1.33}$	26.1	L13	T136$^{1.33}$	30.2	L13	T143$^{1.29}$	29.5
Y13	Y150$^{1.36}$	27.4	Y13	D132$^{1.29}$	23.9	L13	D132$^{1.29}$	24.5	L13	Q146$^{1.32}$	27.8
Y13	Q146$^{1.32}$	18.2	Y13	Y139$^{1.36}$	23.2	L13	Y139$^{1.36}$	21.5	L13	D147$^{1.33}$	16.7
Y13	Y139$^{1.25}$	10.3	Y13	Q135$^{1.32}$	14.0	L13	Q135$^{1.32}$	18.5	L13	Y150$^{1.36}$	16.7
Y13	T143$^{1.29}$	8.2	Y13	L131$^{1.28}$	10.8	L13	F93^{ECD}	10.6	L13	S141$^{1.27}$	11.0
Y13	E142$^{1.28}$	6.2	Y13	S130$^{1.27}$	6.9	L13	A128^{ECD}	5.2	L13	Y139$^{1.25}$	6.4
Y13	S141$^{1.27}$	5.5	Y13	A129$^{1.26}$	5.2	L13	A129$^{1.26}$	3.3	L13	E142$^{1.28}$	6.3
Y13	G144$^{1.30}$	3.7	Y13	A128^{ECD}	3.8				L13	E140$^{1.26}$	4.6
Y13	E140$^{1.26}$	3.1									
R14	D215^{ECL1}	59.9	R14	E204^{ECL1}	62.4	R14	E204^{ECL1}	63.3	R14	D215^{ECL1}	29.8
R14	Y211^{ECL1}	27.7	R14	S202^{ECL1}	27.6	R14	S202^{ECL1}	56.3	R14	Y211^{ECL1}	13.8
R14	F220^{ECL1}	24.4	R14	Q207^{ECL1}	24.1	R14	F200^{ECL1}	44.2	R14	D147$^{1.33}$	13.5
R14	H218^{ECL1}	9.2	R14	F200^{ECL1}	21.3	R14	Q207^{ECL1}	24.4	R14	F220^{ECL1}	4.7
R14	Q214^{ECL1}	5.3	R14	G203^{ECL1}	9.0	R14	L199$^{2.71}$	20.2	R14	T143$^{1.29}$	4.7
R14	D147$^{1.33}$	3.6	R14	C208^{ECL1}	5.8	R14	G203^{ECL1}	19.9	R14	D145$^{1.31}$	3.9
R14	C219^{ECL1}	3.5	R14	D132$^{1.29}$	5.2	R14	C208^{ECL1}	7.4			
			R14	E133$^{1.30}$	4.1	R14	D132$^{1.29}$	7.3			
						R14	D287^{ECL2}	6.3			
						R14	T136$^{1.33}$	2.7			
K15	I83^{ECD}	43.1	K15	E36^{ECD}	59.7	K15	E36^{ECD}	48.4	K15	I83^{ECD}	58.2
K15	F220^{ECL1}	35.6	K15	Q207^{ECL1}	28.3	K15	F93^{ECD}	39.5	K15	F220^{ECL1}	6.3
K15	L80^{ECD}	16.0	K15	L92^{ECD}	19.9	K15	C208^{ECL1}	32.8	K15	D298^{ECL2}	2.6
K15	D298^{ECL2}	5.0	K15	E210^{ECL1}	18.4	K15	D287^{ECL2}	32.6			
K15	M299^{ECL2}	5.0	K15	C37^{ECD}	14.1	K15	Q207^{ECL1}	18.8			
K15	H218^{ECL1}	4.1	K15	C208^{ECL1}	8.7	K15	C37^{ECD}	10.9			
K15	I26^{ECD}	2.4	K15	F93^{ECD}	7.1	K15	Y39^{ECD}	5.7			
K15	F84^{ECD}	2.4	K15	D287^{ECL2}	6.2	K15	D38^{ECD}	4.0			
Q16	I83^{ECD}	65.9	Q16	F93^{ECD}	63.7	Q16	F93^{ECD}	62.0	Q16	I83^{ECD}	77.6
Q16	F84^{ECD}	49.1	Q16	E36^{ECD}	27.4	Q16	L92^{ECD}	25.8	Q16	F84^{ECD}	76.0
Q16	Y139$^{1.25}$	12.9	Q16	L92^{ECD}	25.5	Q16	I89^{ECD}	11.5	Q16	Y139$^{1.25}$	38.0
Q16	D137^{ECD}	5.1	Q16	K127^{ECD}	6.3	Q16	D132$^{1.29}$	9.8	Q16	T143$^{1.29}$	14.7
			Q16	Y39^{ECD}	5.4	Q16	E36^{ECD}	8.8	Q16	E140$^{1.26}$	8.0
						Q16	K127^{ECD}	8.7	Q16	F136^{ECD}	6.8
						Q16	Y39^{ECD}	7.3	Q16	S141$^{1.27}$	6.8
						Q16	Y118^{ECD}	3.8	Q16	D137^{ECD}	6.0

Supplementary Table 7 continued

Contacts Partial Binding

PACAP27-PAC1R

M17	Y139$^{1.25}$	30.1
M17	E142$^{1.28}$	14.9
M17	S141$^{1.27}$	11.2
M17	E140$^{1.26}$	10.7
M17	H218^{ECL1}	9.5
M17	D24^{ECD}	3.9
M17	F136^{ECD}	2.9
A18	H218^{ECL1}	20.8
A18	D24^{ECD}	17.6
A18	N217^{ECL1}	8.9
A18	F27^{ECD}	8.3
A18	S216^{ECL1}	8.1
A18	D215^{ECL1}	7.9
A18	C25^{ECD}	7.9
A18	I26^{ECD}	6.2
A18	Q214^{ECL1}	3.6
A18	C219^{ECL1}	2.3
V19	F84^{ECD}	52.4
V19	L80^{ECD}	29.4
V19	F27^{ECD}	9.8
V19	F81^{ECD}	9.3
V19	I83^{ECD}	5.6
V19	C25^{ECD}	4.3
V19	I26^{ECD}	3.0
K20	F84^{ECD}	48.4
K20	E138^{ECD}	30.6
K20	Y139$^{1.25}$	30.0
K20	E140$^{1.26}$	17.8
K20	F136^{ECD}	13.3
K20	E142$^{1.28}$	10.2
K20	F131^{ECD}	9.1
K20	D147$^{1.33}$	5.1
K20	S141$^{1.27}$	3.8
K21	E142$^{1.28}$	23.6
K21	D147$^{1.33}$	17.6
K21	H218^{ECL1}	17.6
K21	E140$^{1.26}$	16.4
K21	D215^{ECL1}	12.9
K21	S216^{ECL1}	7.1
K21	D24^{ECD}	4.8
K21	S141$^{1.27}$	3.6
K21	C25^{ECD}	3.0
K21	G144$^{1.30}$	3.0
K21	N217^{ECL1}	2.9
K21	Q214^{ECL1}	2.6

PACAP27-VPAC1R

M17	T136$^{1.33}$	10.3
M17	F200^{ECL1}	7.0
A18	C208^{ECL1}	15.6
A18	E204^{ECL1}	9.7
A18	C37^{ECD}	9.6
A18	Q207^{ECL1}	7.2
V19	E36^{ECD}	55.5
V19	F93^{ECD}	38.1
V19	I89^{ECD}	30.6
V19	V40^{ECD}	28.1
V19	C37^{ECD}	25.3
V19	F90^{ECD}	9.0
V19	C208^{ECL1}	4.8
V19	L92^{ECD}	4.8
K20	F93^{ECD}	47.9
K20	E133$^{1.30}$	16.6
K20	L124^{ECD}	11.4
K20	D132$^{1.29}$	10.0
K20	D125^{ECD}	5.6
K21	E204^{ECL1}	29.1
K21	F200^{ECL1}	8.8
K21	D201^{ECL1}	6.1
K21	S205^{ECL1}	5.6
K21	C208^{ECL1}	5.0

VIP-VPAC1R

M17	E204^{ECL1}	13.6
M17	C208^{ECL1}	11.1
M17	S209^{ECL1}	10.0
M17	G203^{ECL1}	6.0
M17	S205^{ECL1}	5.4
M17	E210^{ECL1}	4.1
A18	V40^{ECD}	30.5
A18	C37^{ECD}	5.5
A18	E36^{ECD}	3.5
A18	E204^{ECL1}	2.7
V19	Y39^{ECD}	39.7
V19	V40^{ECD}	36.5
V19	I89^{ECD}	33.7
V19	L92^{ECD}	17.3
V19	I43^{ECD}	17.0
V19	F93^{ECD}	10.4
V19	Y118^{ECD}	10.3
V19	E36^{ECD}	7.6
V19	F90^{ECD}	7.6
V19	C37^{ECD}	2.7
K20	P117^{ECD}	25.3
K20	Y118^{ECD}	22.7
K20	S205^{ECL1}	14.4
K20	E204^{ECL1}	12.2
K20	I89^{ECD}	11.2
K20	D206^{ECL1}	9.4
K20	P119^{ECD}	6.4
K20	F90^{ECD}	6.0
K20	C208^{ECL1}	4.9
K21	E204^{ECL1}	22.7
K21	Q207^{ECL1}	13.2
K21	D287^{ECL2}	11.7
K21	S205^{ECL1}	9.0
K21	C208^{ECL1}	8.9
K21	E210^{ECL1}	8.8
K21	D206^{ECL1}	8.3
K21	S202^{ECL1}	8.3
K21	G203^{ECL1}	6.8
K21	Y283^{ECL2}	3.5

VIP-PAC1R

M17	T143$^{1.29}$	4.3
M17	G144$^{1.30}$	3.0
M17	S141$^{1.27}$	2.8
M17	E142$^{1.28}$	2.7
M17	D145$^{1.31}$	2.0
A18	D24^{ECD}	5.7
A18	L80^{ECD}	3.9
A18	C25^{ECD}	3.8
A18	H218^{ECL1}	2.3
V19	L80^{ECD}	59.7
V19	F84^{ECD}	52.2
V19	I83^{ECD}	25.4
V19	F81^{ECD}	12.7
V19	C25^{ECD}	4.6
V19	D24^{ECD}	2.7
V19	I61^{ECD}	2.4
K20	F84^{ECD}	24.0
K20	D137^{ECD}	21.5
K20	E142$^{1.28}$	12.8
K20	E138^{ECD}	10.7
K20	E140$^{1.26}$	5.8
K20	S141$^{1.27}$	5.8
K20	Y139$^{1.25}$	5.1
K21	H218^{ECL1}	2.0

Contacts Partial Unbinding

PACAP27-PAC1R

M17	Y139$^{1.25}$	7.1
M17	H218^{ECL1}	5.4
M17	S141$^{1.27}$	4.3
M17	F220^{ECL1}	4.1
M17	E142$^{1.28}$	4.0
M17	E140$^{1.26}$	3.2
M17	C219^{ECL1}	2.3
A18	F27^{ECD}	39.4
A18	H218^{ECL1}	31.8
A18	F220^{ECL1}	15.5
A18	C25^{ECD}	3.7
A18	N217^{ECL1}	3.2
A18	I26^{ECD}	2.5
A18	L80^{ECD}	2.1
A18	S216^{ECL1}	2.1
V19	F84^{ECD}	70.6
V19	F81^{ECD}	50.8
V19	L80^{ECD}	46.6
V19	F27^{ECD}	39.5
V19	I83^{ECD}	11.5
V19	F136^{ECD}	5.0
K20	F84^{ECD}	57.3
K20	E140$^{1.26}$	29.1
K20	Y139$^{1.25}$	25.7
K20	E138^{ECD}	16.2
K20	E142$^{1.28}$	9.3
K20	S141$^{1.27}$	3.3
K20	D137^{ECD}	3.0
K21	H218^{ECL1}	27.0
K21	D215^{ECL1}	15.7
K21	E142$^{1.28}$	5.5
K21	S216^{ECL1}	4.3
K21	N217^{ECL1}	2.5
K21	F27^{ECD}	2.3

PACAP27-VPAC1R

M17	E204^{ECL1}	8.4
M17	D132$^{1.29}$	8.0
A18	E204^{ECL1}	38.7
A18	C208^{ECL1}	30.4
A18	Q207^{ECL1}	22.7
A18	E36^{ECD}	9.8
V19	E36^{ECD}	45.2
V19	I89^{ECD}	44.2
V19	F93^{ECD}	38.7
V19	V40^{ECD}	22.8
V19	C37^{ECD}	16.6
V19	C208^{ECL1}	12.9
V19	Y39^{ECD}	10.6
V19	L92^{ECD}	10.5
V19	F90^{ECD}	8.3
K20	F93^{ECD}	47.1
K20	L124^{ECD}	30.0
K20	D132$^{1.29}$	19.6
K20	D126^{ECD}	8.9
K20	G123^{ECD}	7.2
K20	E133$^{1.30}$	5.2
K20	D125^{ECD}	4.4
K20	P117^{ECD}	4.0
K20	K127^{ECD}	3.4
K21	E204^{ECL1}	42.4
K21	E133$^{1.30}$	4.7

VIP-VPAC1R

M17	E204^{ECL1}	20.5
M17	T136$^{1.33}$	9.6
M17	D132$^{1.29}$	7.2
M17	Y118^{ECD}	5.5
M17	E133$^{1.30}$	3.8
A18	E204^{ECL1}	50.4
A18	C208^{ECL1}	45.3
A18	S205^{ECL1}	18.1
A18	Q207^{ECL1}	5.5
V19	I89^{ECD}	44.3
V19	V40^{ECD}	24.8
V19	L92^{ECD}	20.1
V19	E36^{ECD}	16.8
V19	Y39^{ECD}	11.5
V19	F93^{ECD}	10.2
V19	C37^{ECD}	5.4
V19	C208^{ECL1}	4.9
K20	Y118^{ECD}	42.8
K20	D132$^{1.29}$	14.4
K20	P117^{ECD}	10.6
K20	F90^{ECD}	9.0
K20	I89^{ECD}	8.5
K20	E133$^{1.30}$	3.5
K20	E204^{ECL1}	2.8
K20	D126^{ECD}	2.7
K21	E204^{ECL1}	67.7
K21	S205^{ECL1}	17.5
K21	Y118^{ECD}	4.8
K21	C208^{ECL1}	4.1
K21	D201^{ECL1}	3.8
K21	E210^{ECL1}	2.7

VIP-PAC1R

M17	T143$^{1.29}$	25.8
M17	Y139$^{1.25}$	10.1
M17	E142$^{1.28}$	8.4
M17	D147$^{1.33}$	7.0
M17	S141$^{1.27}$	5.6
M17	G144$^{1.30}$	4.8
M17	E140$^{1.26}$	2.6
M17	Q146$^{1.32}$	2.2
A18	F220^{ECL1}	4.0
V19	F84^{ECD}	61.0
V19	L80^{ECD}	57.2
V19	F81^{ECD}	32.9
V19	I83^{ECD}	24.9
K20	F84^{ECD}	26.9
K20	Y139$^{1.25}$	25.0
K20	D137^{ECD}	17.9
K20	T143$^{1.29}$	12.2
K20	E142$^{1.28}$	8.2
K20	F136^{ECD}	6.3
K20	S141$^{1.27}$	3.9
K20	E138^{ECD}	3.2
K21	T143$^{1.29}$	3.5

Supplementary Table 7 continued

Contacts Partial Binding

PACAP27-PAC1R			PACAP27-VPAC1R			VIP-VPAC1R			VIP-PAC1R		
Y22	F27^{ECD}	46.8	Y22	V40^{ECD}	56.2	Y22	V40^{ECD}	52.9	Y22	L80^{ECD}	44.8
Y22	L80^{ECD}	24.7	Y22	E44^{ECD}	36.7	Y22	I43^{ECD}	43.0	Y22	C25^{ECD}	34.3
Y22	F81^{ECD}	23.8	Y22	Q41^{ECD}	27.2	Y22	E44^{ECD}	39.1	Y22	F27^{ECD}	33.0
Y22	C25^{ECD}	21.9	Y22	C37^{ECD}	21.9	Y22	H47^{ECD}	19.0	Y22	N60^{ECD}	25.3
Y22	Q31^{ECD}	16.6	Y22	I43^{ECD}	15.6	Y22	C37^{ECD}	12.8	Y22	K28^{ECD}	24.7
Y22	I26^{ECD}	11.3	Y22	C208^{ECL1}	13.6	Y22	I89^{ECD}	12.6	Y22	Q31^{ECD}	10.1
Y22	N60^{ECD}	11.0	Y22	S205^{ECL1}	12.8	Y22	E36^{ECD}	8.9	Y22	I61^{ECD}	8.8
Y22	D24^{ECD}	10.5	Y22	E204^{ECL1}	5.5	Y22	L70^{ECD}	5.1	Y22	D24^{ECD}	8.3
Y22	E30^{ECD}	9.8	Y22	I89^{ECD}	5.3	Y22	W67^{ECD}	3.7	Y22	F81^{ECD}	8.0
Y22	K28^{ECD}	9.4	Y22	F90^{ECD}	4.8				Y22	I26^{ECD}	4.2
Y22	H218^{ECL1}	5.5							Y22	P78^{ECD}	2.3
Y22	Q214^{ECL1}	3.5							Y22	W58^{ECD}	2.1
Y22	I61^{ECD}	3.2									
Y22	N217^{ECL1}	3.0									
Y22	P78^{ECD}	2.3									
L23	F84^{ECD}	52.9	L23	I89^{ECD}	35.3	L23	L70^{ECD}	35.5	L23	N60^{ECD}	41.1
L23	F81^{ECD}	45.5	L23	V40^{ECD}	25.9	L23	I89^{ECD}	30.3	L23	F81^{ECD}	35.6
L23	F136^{ECD}	27.4	L23	F90^{ECD}	24.6	L23	Y118^{ECD}	24.2	L23	F84^{ECD}	20.0
L23	L80^{ECD}	27.0	L23	L124^{ECD}	22.3	L23	F90^{ECD}	21.6	L23	L80^{ECD}	15.9
L23	Y139$^{1.25}$	24.9	L23	L70^{ECD}	18.5	L23	I43^{ECD}	17.2	L23	I61^{ECD}	8.8
L23	F131^{ECD}	9.4	L23	F93^{ECD}	18.4	L23	P119^{ECD}	16.7	L23	F27^{ECD}	7.6
L23	F27^{ECD}	7.3	L23	I43^{ECD}	8.0	L23	W67^{ECD}	10.0	L23	Y139$^{1.25}$	3.6
L23	C25^{ECD}	6.4				L23	I120^{ECD}	8.8			
L23	D137^{ECD}	5.6				L23	V40^{ECD}	4.2			
A24	Y139$^{1.25}$	14.3	A24	L124^{ECD}	7.1	N24	C208^{ECL1}	13.3	N24	N60^{ECD}	3.4
A24	F131^{ECD}	7.6				N24	P119^{ECD}	12.0			
A24	E140$^{1.26}$	5.5				N24	P115^{ECD}	11.1			
A24	G144$^{1.30}$	5.1				N24	Y118^{ECD}	10.2			
A24	S141$^{1.27}$	3.7				N24	E204^{ECL1}	9.7			
A24	F136^{ECD}	2.4				N24	G116^{ECD}	9.0			
						N24	S205^{ECL1}	7.3			
						N24	P117^{ECD}	5.8			
						N24	D206^{ECL1}	4.3			
						N24	Q207^{ECL1}	4.0			
A25	F27^{ECD}	18.5				S25	C208^{ECL1}	15.0	S25	K28^{ECD}	19.1
A25	N60^{ECD}	13.1				S25	S209^{ECL1}	8.9	S25	N60^{ECD}	11.5
A25	Y148$^{1.34}$	7.1				S25	Q207^{ECL1}	4.8	S25	W58^{ECD}	3.4
A25	D145$^{1.31}$	3.2							S25	C25^{ECD}	2.2
A25	H218^{ECL1}	2.5									
V26	N60^{ECD}	37.5	V26	L70^{ECD}	26.8	I26	L70^{ECD}	24.5	I26	K28^{ECD}	39.1
V26	I61^{ECD}	21.5	V26	E44^{ECD}	23.9	I26	H47^{ECD}	23.3	I26	F27^{ECD}	21.6
V26	F27^{ECD}	17.6	V26	V40^{ECD}	17.5	I26	W67^{ECD}	18.6	I26	W58^{ECD}	18.8
V26	K28^{ECD}	13.7	V26	N69^{ECD}	17.1	I26	P119^{ECD}	9.9	I26	N60^{ECD}	14.9
V26	F81^{ECD}	11.2	V26	H47^{ECD}	16.1	I26	I43^{ECD}	9.7	I26	C25^{ECD}	14.2
V26	C25^{ECD}	10.0	V26	I43^{ECD}	15.3	I26	V40^{ECD}	8.2	I26	Q31^{ECD}	9.0
V26	F136^{ECD}	6.4				I26	E44^{ECD}	7.0	I26	L80^{ECD}	5.2
V26	D24^{ECD}	5.2				I26	W110^{ECD}	6.0			
V26	L80^{ECD}	4.2				I26	P117^{ECD}	5.7			
						I26	Y118^{ECD}	5.1			
						I26	H112^{ECD}	4.4			

Contacts Partial Unbinding

PACAP27-PAC1R			PACAP27-VPAC1R			VIP-VPAC1R			VIP-PAC1R		
Y22	F27^{ECD}	84.9	Y22	V40^{ECD}	61.2	Y22	V40^{ECD}	43.5	Y22	N60^{ECD}	42.9
Y22	N60^{ECD}	62.6	Y22	E44^{ECD}	24.2	Y22	E44^{ECD}	29.9	Y22	F27^{ECD}	41.3
Y22	F81^{ECD}	36.0	Y22	I43^{ECD}	23.0	Y22	I43^{ECD}	26.6	Y22	K28^{ECD}	37.7
Y22	Q31^{ECD}	32.2	Y22	C37^{ECD}	20.1	Y22	I89^{ECD}	11.4	Y22	L80^{ECD}	31.9
Y22	C25^{ECD}	20.4	Y22	C208^{ECL1}	17.9	Y22	C37^{ECD}	10.7	Y22	C25^{ECD}	25.8
Y22	K28^{ECD}	18.3	Y22	Q41^{ECD}	15.9	Y22	S205^{ECL1}	10.6	Y22	Q31^{ECD}	22.1
Y22	H218^{ECL1}	15.6	Y22	E36^{ECD}	15.5	Y22	C208^{ECL1}	9.8	Y22	W58^{ECD}	8.5
Y22	I61^{ECD}	14.7	Y22	I89^{ECD}	9.7	Y22	E36^{ECD}	5.4	Y22	F81^{ECD}	5.8
Y22	L80^{ECD}	10.5	Y22	S205^{ECL1}	9.6	Y22	H47^{ECD}	5.3	Y22	D24^{ECD}	4.2
Y22	W58^{ECD}	3.4	Y22	L70^{ECD}	6.2	Y22	Q41^{ECD}	4.8			
Y22	P78^{ECD}	2.1				Y22	Y39^{ECD}	4.6			
						Y22	W67^{ECD}	3.8			
						Y22	L70^{ECD}	3.4			
						Y22	S209^{ECL1}	2.9			
L23	F136^{ECD}	45.9	L23	F90^{ECD}	36.2	L23	L70^{ECD}	38.0	L23	N60^{ECD}	59.9
L23	F84^{ECD}	37.4	L23	I89^{ECD}	32.5	L23	F90^{ECD}	37.8	L23	F81^{ECD}	47.3
L23	F131^{ECD}	25.8	L23	L70^{ECD}	29.4	L23	I89^{ECD}	35.1	L23	I61^{ECD}	15.6
L23	F81^{ECD}	23.0	L23	L124^{ECD}	27.7	L23	Y118^{ECD}	30.5	L23	F84^{ECD}	13.2
L23	I61^{ECD}	21.2	L23	F93^{ECD}	20.1	L23	I43^{ECD}	21.8	L23	L80^{ECD}	8.0
L23	Y139$^{1.25}$	16.2	L23	V40^{ECD}	14.6	L23	P119^{ECD}	7.3	L23	F27^{ECD}	8.0
L23	N60^{ECD}	5.8	L23	I43^{ECD}	9.0	L23	V40^{ECD}	6.6	L23	Y139$^{1.25}$	3.1
L23	Y130^{ECD}	3.6	L23	P117^{ECD}	7.5				L23	W58^{ECD}	2.1
L23	L80^{ECD}	3.0	L23	N69^{ECD}	4.2						
			L23	I120^{ECD}	3.8						
A24	Y139$^{1.25}$	9.1	A24	L124^{ECD}	5.6	N24	Y118^{ECD}	32.6	N24	N60^{ECD}	4.7
						N24	P117^{ECD}	16.2	N24	T143$^{1.29}$	2.1
						N24	P119^{ECD}	14.3			
						N24	G116^{ECD}	7.8			
						N24	P115^{ECD}	7.3			
						N24	C208^{ECL1}	3.4			
A25	N60^{ECD}	7.3							S25	N60^{ECD}	34.9
A25	H218^{ECL1}	6.7							S25	K28^{ECD}	13.6
									S25	W58^{ECD}	4.4
V26	N60^{ECD}	49.1	V26	L70^{ECD}	31.6	I26	W67^{ECD}	25.0	I26	W58^{ECD}	18.0
V26	F131^{ECD}	20.0	V26	V40^{ECD}	17.3	I26	L70^{ECD}	23.7	I26	K28^{ECD}	17.5
V26	Y130^{ECD}	14.7	V26	N69^{ECD}	16.9	I26	H47^{ECD}	20.9	I26	F27^{ECD}	9.3
V26	I61^{ECD}	13.1	V26	E44^{ECD}	13.2	I26	I43^{ECD}	14.4	I26	Q31^{ECD}	8.5
V26	F127^{ECD}	8.3	V26	I43^{ECD}	13.0	I26	V40^{ECD}	11.8	I26	N60^{ECD}	5.8
V26	F81^{ECD}	3.1	V26	H47^{ECD}	10.5	I26	E44^{ECD}	10.9	I26	C25^{ECD}	3.9
V26	F27^{ECD}	2.8	V26	W67^{ECD}	5.5						
V26	F136^{ECD}	2.2									

Supplementary Table 7 continued

Contacts Partial Binding

PACAP27-PAC1R		
L27	F136^{ECD}	27.7
L27	N60^{ECD}	23.5
L27	I61^{ECD}	20.0
L27	Y139$^{1.25}$	12.6
L27	D137^{ECD}	10.6
L27	F81^{ECD}	10.1
L27	F27^{ECD}	9.5
L27	F131^{ECD}	9.2
L27	E138^{ECD}	7.9
L27	K28^{ECD}	6.2
L27	Y130^{ECD}	6.1
L27	L80^{ECD}	5.3

PACAP27-VPAC1R		
L27	L70^{ECD}	38.8
L27	T71^{ECD}	18.7
L27	F90^{ECD}	18.1
L27	N69^{ECD}	17.9
L27	A121^{ECD}	17.2
L27	H47^{ECD}	16.5
L27	L124^{ECD}	14.9
L27	I120^{ECD}	13.7
L27	P117^{ECD}	10.0
L27	I43^{ECD}	6.7

VIP-VPAC1R		
L27	P119^{ECD}	20.0
L27	L113^{ECD}	18.5
L27	P117^{ECD}	18.2
L27	Y118^{ECD}	15.7
L27	L70^{ECD}	15.2
L27	W110^{ECD}	13.9
L27	H112^{ECD}	11.3
L27	W67^{ECD}	6.3
L27	I120^{ECD}	5.6
L27	P115^{ECD}	4.9
L27	G116^{ECD}	3.8
L27	T111^{ECD}	3.5
N28	C208^{ECL1}	12.3
N28	H112^{ECD}	10.0
N28	P117^{ECD}	9.3
N28	L113^{ECD}	9.1
N28	P115^{ECD}	8.1
N28	S205^{ECL1}	5.3
N28	Y118^{ECD}	4.4
N28	P119^{ECD}	3.8
N28	S209^{ECL1}	3.7
N28	Q207^{ECL1}	3.4
N28	G116^{ECD}	3.0

VIP-PAC1R		
L27	W58^{ECD}	40.0
L27	N60^{ECD}	19.9
L27	K28^{ECD}	17.7
L27	Q31^{ECD}	12.8
L27	L35^{ECD}	12.6
L27	A32^{ECD}	6.5
L27	F27^{ECD}	6.1
N28	K28^{ECD}	19.4
N28	W58^{ECD}	17.2
N28	N60^{ECD}	8.3
N28	Q31^{ECD}	7.8
N28	L35^{ECD}	6.2
N28	F27^{ECD}	4.8
N28	A32^{ECD}	4.5
N28	D24^{ECD}	2.5

Contacts Partial Unbinding

PACAP27-PAC1R		
L27	F131^{ECD}	25.1
L27	Y130^{ECD}	14.9
L27	Y139$^{1.25}$	12.9
L27	F136^{ECD}	9.6
L27	F127^{ECD}	8.1
L27	F84^{ECD}	5.6
L27	N60^{ECD}	4.8
L27	I61^{ECD}	3.0
L27	H129^{ECD}	2.4

PACAP27-VPAC1R		
L27	L70^{ECD}	32.7
L27	N69^{ECD}	22.7
L27	L124^{ECD}	16.7
L27	P117^{ECD}	13.4
L27	H47^{ECD}	12.3
L27	I120^{ECD}	12.0
L27	A121^{ECD}	11.0
L27	T71^{ECD}	9.5
L27	F90^{ECD}	8.9
L27	P115^{ECD}	6.7
L27	I43^{ECD}	5.6
L27	G116^{ECD}	4.7
L27	W67^{ECD}	3.8

VIP-VPAC1R		
L27	P119^{ECD}	25.8
L27	L70^{ECD}	22.1
L27	Y118^{ECD}	22.0
L27	I120^{ECD}	14.9
L27	W67^{ECD}	11.9
L27	L113^{ECD}	9.9
L27	P117^{ECD}	7.2
L27	P115^{ECD}	5.6
L27	N69^{ECD}	4.4
L27	E114^{ECD}	3.2
L27	W110^{ECD}	2.7
N28	Y118^{ECD}	3.9
N28	P115^{ECD}	3.8
N28	P117^{ECD}	3.8
N28	P119^{ECD}	3.6

VIP-PAC1R		
L27	W58^{ECD}	48.3
L27	L35^{ECD}	23.3
L27	K28^{ECD}	11.2
L27	N60^{ECD}	9.3
L27	Q31^{ECD}	7.6
L27	A32^{ECD}	7.4
N28	W58^{ECD}	40.0
N28	L35^{ECD}	20.7
N28	K28^{ECD}	16.6
N28	Q31^{ECD}	11.1
N28	N60^{ECD}	8.3
N28	A32^{ECD}	7.6
N28	F27^{ECD}	4.0
N28	C25^{ECD}	2.1

Supplementary Table 8: Hydrogen bonds between receptor and peptide residues during MD partial binding and unbinding simulations. Columns are sorted by peptide residues and highest to lowest occupancy. Contacts are shown as occupancy of MD frames (in percent), and background is coloured on a scale with highest occupancy in red and lowest occupancy in white. Receptor residues are labelled according to the Wootten numbering system and background is coloured by residue position (TMs, ECLs and ECD, see legend).

Legend: TM1, TM2, TM3, TM4, TM5, TM6, TM7, ECD, ECL1, ECL2, ECL3; Min % – Max %

Hydrogen bonds partial binding

PACAP27-PAC1R

Peptide	Receptor	Occupancy (%)
H1	D301^{ECL2}	4.3
S2	D298^{ECL2}	1.0
S2	D301^{ECL2}	2.9
D3	K378$^{7.35}$	7.9
D3	T294^{ECL2}	2.1
D3	R379$^{7.36}$	1.5
T7	Y150$^{1.36}$	1.8
T7	T294^{ECL2}	1.0
S9	D147$^{1.33}$	9.7
S9	Y150$^{1.36}$	1.2
Y10	D215^{ECL1}	9.1
Y10	Q214^{ECL1}	2.0
Y10	E140$^{1.26}$	1.5
S11	H218^{ECL1}	2.4
S11	N217^{ECL1}	1.3
S11	Q214^{ECL1}	1.1
R12	D87^{ECD}	36.6
R12	D147$^{1.33}$	9.4

PACAP27-VPAC1R

Peptide	Receptor	Occupancy (%)
D3	N290$^{5.32}$	1.7
D3	N363^{ECL3}	1.2
D3	T288^{ECL2}	1.0
T7	N290$^{5.32}$	1.7
D8	T288^{ECL2}	3.0
D8	Y283^{ECL2}	1.4
S9	D132$^{1.29}$	10.0
S9	Y139$^{1.36}$	3.6
S9	Q135$^{1.32}$	1.7
S9	E36^{ECD}	1.7
Y10	D132$^{1.29}$	4.0
Y10	E36^{ECD}	3.7
Y10	D287^{ECL2}	2.7
Y10	Q207^{ECL1}	2.2
Y10	E133$^{1.30}$	2.2
S11	E204^{ECL1}	2.3
S11	Q207^{ECL1}	2.2
R12	E36^{ECD}	40.1
R12	D132$^{1.29}$	16.2
R12	D287^{ECL2}	6.0
R12	E210^{ECL1}	3.2
R12	Q207^{ECL1}	1.7
R12	E204^{ECL1}	1.3

VIP-VPAC1R

Peptide	Receptor	Occupancy (%)
H1	D362^{ECL3}	4.5
H1	E367$^{7.36}$	2.4
S2	D362^{ECL3}	1.3
D3	K365$^{7.34}$	9.0
D3	K369$^{7.38}$	7.5
T7	Y283^{ECL2}	1.5
D8	T288^{ECL2}	2.6
D8	N290$^{5.32}$	1.6
N9	Y283^{ECL2}	5.1
N9	Y139$^{1.36}$	1.6
N9	D132$^{1.29}$	1.3
Y10	D206^{ECL1}	2.9
Y10	E210^{ECL1}	2.2
Y10	D287^{ECL2}	1.4
Y10	N290$^{5.32}$	1.1
T11	T288^{ECL2}	1.5
R12	E36^{ECD}	22.8
R12	D132$^{1.29}$	7.3
R12	Y39^{ECD}	5.1
R12	D38^{ECD}	1.0

VIP-PAC1R

Peptide	Receptor	Occupancy (%)
H1	E91^{ECD}	3.7
H1	N300^{ECL2}	3.5
H1	N375^{ECL3}	3.2
H1	E374^{ECL3}	1.4
H1	D147$^{1.33}$	1.3
H1	E385$^{7.42}$	1.2
S2	E91^{ECD}	4.0
S2	E374^{ECL3}	2.4
S2	N300^{ECL2}	1.4
S2	D147$^{1.33}$	1.3
D3	K310$^{5.40}$	6.2
D3	W306$^{5.36}$	4.6
D3	K206$^{2.67}$	2.2
D3	K378$^{7.35}$	2.0
D3	K154$^{1.40}$	1.1
D8	R379$^{7.36}$	5.1
D8	K378$^{7.35}$	3.6
D8	Y139$^{1.25}$	2.0
D8	S141$^{1.27}$	1.9
N9	Y139$^{1.25}$	3.5
N9	S141$^{1.27}$	2.4
N9	R379$^{7.36}$	1.7
N9	Q146$^{1.32}$	1.4
Y10	D215^{ECL1}	5.1
Y10	D298^{ECL2}	1.9
R12	D87^{ECD}	53.1
R12	D147$^{1.33}$	7.2
R12	E140$^{1.26}$	3.0
R12	D110^{ECD}	2.0
R12	E142$^{1.28}$	1.9
R12	R82^{ECD}	1.2
R12	D145$^{1.31}$	1.0

Hydrogen bonds partial unbinding

PACAP27-PAC1R

Peptide	Receptor	Occupancy (%)
H1	N300^{ECL2}	8.0
H1	E374^{ECL3}	5.9
H1	Y241$^{3.44}$	3.8
H1	W306$^{5.36}$	2.8
H1	D301^{ECL2}	1.2
H1	K310$^{5.40}$	1.0
H1	T303$^{5.33}$	1.0
S2	E385$^{7.42}$	5.2
S2	K378$^{7.35}$	4.1
S2	N375^{ECL3}	2.8
S2	E374^{ECL3}	2.7
D3	K378$^{7.35}$	12.8
D3	R199$^{2.60}$	10.4
D3	Y161$^{1.47}$	6.5
D3	K206$^{2.67}$	4.0
D3	R379$^{7.36}$	1.5
T7	K206$^{2.67}$	6.5
T7	D298^{ECL2}	5.9
T7	Y211^{ECL1}	5.8
S9	Y150$^{1.36}$	13.6
S9	E142$^{1.28}$	2.6
Y10	D215^{ECL1}	8.1
Y10	D147$^{1.33}$	3.9
Y10	Y211^{ECL1}	2.3
S11	Y211^{ECL1}	6.3
S11	D298^{ECL2}	3.6
R12	D87^{ECD}	28.6
R12	D301^{ECL2}	9.0
R12	E142$^{1.28}$	4.7

PACAP27-VPAC1R

Peptide	Receptor	Occupancy (%)
H1	W294$^{5.36}$	5.3
H1	E367$^{7.36}$	2.7
H1	K369$^{7.38}$	2.6
H1	K298$^{5.40}$	2.5
H1	D362^{ECL3}	2.3
H1	E373$^{7.42}$	2.1
S2	E373$^{7.42}$	15.7
S2	E367$^{7.36}$	5.0
S2	Y139$^{1.36}$	3.7
S2	K369$^{7.38}$	2.7
S2	D362^{ECL3}	1.0
D3	R188$^{2.60}$	2.5
D3	K369$^{7.38}$	2.0
D3	K195$^{2.67}$	1.9
D3	N290$^{5.32}$	1.5
D3	Y139$^{1.36}$	1.3
T7	K195$^{2.67}$	1.9
T7	Y139$^{1.36}$	1.2
D8	T288^{ECL2}	6.4
D8	K127^{ECD}	6.1
S9	Y139$^{1.36}$	8.9
S9	D132$^{1.29}$	7.6
S9	E36^{ECD}	2.6
Y10	D132$^{1.29}$	4.7
Y10	D201^{ECL1}	3.3
Y10	Y39^{ECD}	2.1
Y10	E204^{ECL1}	1.8
Y10	S205^{ECL1}	1.3
Y10	T136$^{1.33}$	1.3
Y10	Q207^{ECL1}	1.2
S11	D287^{ECL2}	10.0
S11	Q207^{ECL1}	3.2
R12	D132$^{1.29}$	14.0
R12	D287^{ECL2}	12.7
R12	E36^{ECD}	11.4
R12	N290$^{5.32}$	2.3
R12	E210^{ECL1}	1.0

VIP-VPAC1R

Peptide	Receptor	Occupancy (%)
H1	W294$^{5.36}$	3.9
H1	K298$^{5.40}$	3.6
H1	K369$^{7.38}$	2.2
H1	Q223$^{3.37}$	2.0
S2	E373$^{7.42}$	18.0
S2	K369$^{7.38}$	4.5
D3	R188$^{2.60}$	5.1
D3	Y139$^{1.36}$	3.4
T7	K195$^{2.67}$	6.0
D8	T288^{ECL2}	15.5
N9	Y139$^{1.36}$	11.8
N9	D132$^{1.29}$	3.8
T11	D287^{ECL2}	15.5
R12	E36^{ECD}	29.4
R12	D287^{ECL2}	4.7
R12	D132$^{1.29}$	3.4

VIP-PAC1R

Peptide	Receptor	Occupancy (%)
H1	N300^{ECL2}	4.9
H1	E374^{ECL3}	4.1
H1	D215^{ECL1}	2.6
H1	W306$^{5.36}$	1.7
H1	K310$^{5.40}$	1.6
H1	N375^{ECL3}	1.1
S2	E385$^{7.42}$	5.9
S2	E374^{ECL3}	2.3
S2	D215^{ECL1}	1.7
D3	K206$^{2.67}$	10.2
D3	R199$^{2.60}$	6.5
D3	K154$^{1.40}$	6.4
D3	Y211^{ECL1}	2.3
D3	Y161$^{1.47}$	1.6
D3	Y157$^{1.43}$	1.5
T7	K206$^{2.67}$	5.8
T7	Y211^{ECL1}	4.9
D8	R379$^{7.36}$	1.4
N9	Y150$^{1.36}$	9.9
N9	K378$^{7.35}$	2.3
N9	R379$^{7.36}$	2.2
N9	Q146$^{1.32}$	1.3
N9	N375^{ECL3}	1.2
N9	S141$^{1.27}$	1.0
Y10	D147$^{1.33}$	16.4
Y10	D215^{ECL1}	1.1
R12	D87^{ECD}	64.5
R12	Y139$^{1.25}$	1.5
R12	E140$^{1.26}$	1.2

Supplementary Table 8 continued

Hydrogen bonds partial binding

PACAP27-PAC1R			PACAP27-VPAC1R			VIP-VPAC1R			VIP-PAC1R		
Y13	D87^{ECD}	16.1	Y13	E133$^{1.30}$	8.8						
Y13	D147$^{1.33}$	4.2	Y13	D132$^{1.29}$	3.8						
Y13	D215^{ECL1}	1.3	Y13	T136$^{1.33}$	1.8						
Y13	S141$^{1.27}$	1.2									
R14	D215^{ECL1}	22.2	R14	E204^{ECL1}	39.0	R14	D287^{ECL2}	18.1	R14	D215^{ECL1}	9.6
R14	D24^{ECD}	22.2	R14	E133$^{1.30}$	8.9	R14	E210^{ECL1}	11.5	R14	D147$^{1.33}$	2.6
R14	E142$^{1.28}$	20.1	R14	D287^{ECL2}	7.1	R14	E204^{ECL1}	8.8	R14	D298^{ECL2}	1.2
R14	Q214^{ECL1}	6.2	R14	Q207^{ECL1}	4.2	R14	D196$^{2.68}$	4.0	R14	H218^{ECL1}	1.0
R14	D147$^{1.33}$	5.1	R14	E210^{ECL1}	2.0	R14	S202^{ECL1}	3.1			
R14	E140$^{1.26}$	4.8	R14	D132$^{1.29}$	1.3	R14	E36^{ECD}	1.7			
R14	H218^{ECL1}	4.5									
K15	D24^{ECD}	8.5	K15	E36^{ECD}	39.9	K15	D38^{ECD}	53.3	K15	D24^{ECD}	4.8
K15	N217^{ECL1}	2.0	K15	E210^{ECL1}	6.0	K15	E36^{ECD}	14.9	K15	E79^{ECD}	3.7
K15	H218^{ECL1}	1.0	K15	E204^{ECL1}	5.3	K15	D287^{ECL2}	7.3	K15	D215^{ECL1}	3.3
									K15	H218^{ECL1}	1.2
Q16	Y139$^{1.25}$	5.5	Q16	E36^{ECD}	13.6	Q16	Y118^{ECD}	7.9	Q16	Y139$^{1.25}$	8.1
			Q16	D132$^{1.29}$	3.2				Q16	S141$^{1.27}$	1.7
			Q16	T136$^{1.33}$	1.2				Q16	E138^{ECD}	1.0
			Q16	S130$^{1.27}$	1.1				Q16	E140$^{1.26}$	1.0
K20	E138^{ECD}	28.3	K20	E133$^{1.30}$	14.8	K20	E204^{ECL1}	11.1	K20	D137^{ECD}	19.8
K20	E140$^{1.26}$	13.8	K20	D132$^{1.29}$	9.6	K20	D206^{ECL1}	9.3	K20	E142$^{1.28}$	11.1
K20	D147$^{1.33}$	4.6	K20	D125^{ECD}	5.4	K20	S205^{ECL1}	6.6	K20	E138^{ECD}	10.2
K20	E142$^{1.28}$	4.0				K20	E210^{ECL1}	1.2	K20	E140$^{1.26}$	4.3
K20	S141$^{1.27}$	1.0							K20	S141$^{1.27}$	2.3
K21	E142$^{1.28}$	21.7	K21	E204^{ECL1}	25.3	K21	E204^{ECL1}	16.3	K21	D215^{ECL1}	1.9
K21	D147$^{1.33}$	17.3	K21	D201^{ECL1}	5.7	K21	D287^{ECL2}	11.0	K21	E142$^{1.28}$	1.0
K21	E140$^{1.26}$	14.9				K21	E210^{ECL1}	8.6			
K21	H218^{ECL1}	8.2				K21	D206^{ECL1}	6.7			
K21	D215^{ECL1}	7.9				K21	Q207^{ECL1}	1.6			
K21	D24^{ECD}	2.1				K21	S202^{ECL1}	1.1			
K21	E138^{ECD}	1.1									
Y22	Q31^{ECD}	7.8	Y22	E44^{ECD}	34.8	Y22	E44^{ECD}	17.4	Y22	Q31^{ECD}	2.9
Y22	D24^{ECD}	6.8	Y22	Q41^{ECD}	8.8	Y22	H47^{ECD}	12.9	Y22	D24^{ECD}	1.6
Y22	E30^{ECD}	2.0				Y22	E36^{ECD}	3.3			
Y22	N60^{ECD}	1.1									
						N24	Y118^{ECD}	8.1	N24	N60^{ECD}	1.2
						N24	E204^{ECL1}	3.2	N24	Y139$^{1.25}$	1.2
									S25	K28^{ECD}	7.1
						N28	S205^{ECL1}	1.6	N28	K28^{ECD}	2.5
									N28	N60^{ECD}	2.2
									N28	D24^{ECD}	1.2

Hydrogen bonds partial unbinding

PACAP27-PAC1R			PACAP27-VPAC1R			VIP-VPAC1R			VIP-PAC1R		
Y13	D147$^{1.33}$	26.6	Y13	T136$^{1.33}$	14.5						
Y13	E142$^{1.28}$	1.2	Y13	D132$^{1.29}$	14.3						
Y13	E140$^{1.26}$	1.0	Y13	E133$^{1.30}$	1.5						
			Y13	E204^{ECL1}	1.3						
			Y13	S130$^{1.27}$	1.3						
R14	D215^{ECL1}	59.3	R14	E204^{ECL1}	58.3	R14	E204^{ECL1}	17.5	R14	D215^{ECL1}	29.7
R14	D147$^{1.33}$	3.6	R14	D132$^{1.29}$	4.6	R14	Q207^{ECL1}	9.0	R14	D147$^{1.33}$	13.2
R14	Q214^{ECL1}	2.1	R14	E133$^{1.30}$	3.8	R14	D132$^{1.29}$	7.3	R14	D145$^{1.31}$	3.9
R14	E142$^{1.28}$	1.7	R14	Q207^{ECL1}	1.4	R14	D287^{ECL2}	5.6	R14	E142$^{1.28}$	1.5
R14	H218^{ECL1}	1.6	R14	E36^{ECD}	1.2	R14	E210^{ECL1}	2.0			
K15	D298^{ECL2}	4.9	K15	E36^{ECD}	39.7	K15	E36^{ECD}	40.6	K15	D298^{ECL2}	2.5
			K15	E210^{ECL1}	17.5	K15	D287^{ECL2}	31.6	K15	E79^{ECD}	1.0
			K15	D287^{ECL2}	5.6	K15	D38^{ECD}	3.8			
			K15	Q207^{ECL1}	4.2	K15	E210^{ECL1}	2.2			
Q16	D137^{ECD}	2.4	Q16	E36^{ECD}	10.2	Q16	K127^{ECD}	6.1	Q16	Y139$^{1.25}$	11.0
Q16	Y139$^{1.25}$	1.3	Q16	K127^{ECD}	3.3	Q16	D132$^{1.29}$	4.9	Q16	T143$^{1.29}$	2.5
			Q16	Y39^{ECD}	2.4	Q16	E36^{ECD}	3.0	Q16	S141$^{1.27}$	2.0
									Q16	Q146$^{1.32}$	1.4
K20	E140$^{1.26}$	23.1	K20	D132$^{1.29}$	18.6	K20	D132$^{1.29}$	13.7	K20	D137^{ECD}	14.8
K20	E138^{ECD}	9.9	K20	E133$^{1.30}$	5.1	K20	E133$^{1.30}$	3.2	K20	E142$^{1.28}$	7.0
K20	E142$^{1.28}$	8.5	K20	D125^{ECD}	4.1	K20	E204^{ECL1}	2.7	K20	T143$^{1.29}$	3.9
K20	D137^{ECD}	2.7	K20	D126^{ECD}	1.4	K20	Y118^{ECD}	2.2	K20	E138^{ECD}	2.7
K20	S141$^{1.27}$	1.2				K20	D126^{ECD}	2.1	K20	Y139$^{1.25}$	2.2
									K20	E140$^{1.26}$	1.1
K21	D215^{ECL1}	13.3	K21	E204^{ECL1}	36.0	K21	E204^{ECL1}	61.9	K21	D145$^{1.31}$	2.1
K21	E142$^{1.28}$	5.0	K21	E133$^{1.30}$	4.6	K21	D201^{ECL1}	2.9			
K21	H218^{ECL1}	3.4	K21	D132$^{1.29}$	1.9	K21	E210^{ECL1}	2.6			
K21	E140$^{1.26}$	1.4				K21	S205^{ECL1}	2.6			
K21	E213^{ECL1}	1.4									
Y22	N60^{ECD}	10.8	Y22	E44^{ECD}	22.4	Y22	E44^{ECD}	21.3	Y22	Q31^{ECD}	11.9
Y22	Q31^{ECD}	6.9	Y22	Q41^{ECD}	6.2				Y22	D24^{ECD}	1.4
			Y22	E36^{ECD}	4.1						
						N24	Y118^{ECD}	15.5	N24	N60^{ECD}	1.1
									S25	K28^{ECD}	5.6
									S25	N60^{ECD}	1.5
									N28	N60^{ECD}	3.5
									N28	W58^{ECD}	2.9
									N28	K28^{ECD}	2.3

Supplementary Table 9: MM-GBSA binding energy of PACAP27 or VIP in complex to VPAC1 or PAC1. Results refer to equilibrium simulations **without the disulphide** and **in the presence of the disulphide** bond between ECD and ECL1. Asterisk refers to the homology modelled PAC1R-VIP complex.

**Without disulfide**		
Receptor	Agonist	MM-GBSA Binding Energy (Kcal/mol)
VPAC1	PACAP27	-112.18 ± 15.40
PAC1	PACAP27	-99.09 ± 16.13
VPAC1	VIP	-91.25 ± 14.02
PAC1*	VIP*	-77.52 ± 15.29

**With disulfide**		
Receptor	Agonist	MM-GBSA Binding Energy (Kcal/mol)
VPAC1	PACAP27	-96.82 ± 15.68
PAC1	PACAP27	-90.49 ± 15.47
VPAC1	VIP	-109.41 ± 20.73
PAC1*	VIP*	-99.78± 26.48

Supplementary Table 10: Generic contacts and hydrogen bonds between receptor and G protein residues during MD equilibrium simulations (ECD-ECL1 disulphide bond absent). Columns are sorted by G protein residues as well as highest to lowest occupancy. Contacts/Hydrogen bonds are shown as occupancy of MD frames (in percent), and background is coloured on a scale with highest occupancy in red and lowest occupancy in white. Receptor residues are labelled according to the Wootten numbering system and background is coloured by residue position (TMs and ICLs).

MD generic contacts (disulphide bond absent)									MD Hydrogen bonds (disulphide bond absent)								
VPAC1R:VIP:Gs			VPAC1R:PACAP27:Gs			PAC1R:PACAP27:Gs			VPAC1R:VIP:Gs			VPAC1R:PACAP27:Gs			PAC1R:PACAP27:Gs		
									S250$^{4.37}$	Q35	5.2						
E251$^{4.38}$	R38	61.3	E251$^{4.38}$	R38	44.4	F260^{ICL2}	R38	38.2	E251$^{4.38}$	R38	60.7	E251$^{4.38}$	R38	43.6	E262^{ICL2}	R38	7.2
						R263^{ICL2}	R38	31.9							E257$^{3.60}$	R38	6.2
						P261^{ICL2}	R38	27.2									
						F260^{ICL2}	A39	57.5									
F248^{ICL2}	H41	61.2	F248^{ICL2}	H41	38.7	F259^{ICL2}	H41	61.4									
						F260^{ICL2}	K216	38.0									
F248^{ICL2}	V217	65.5	F248^{ICL2}	V217	43.4	F259^{ICL2}	V217	50.9									
						F260^{ICL2}	V217	39.3									
									W406$^{7.75}$	D291	11.4						
R329^{ICL3}	E322	32.1							K330^{ICL3}	E322	22.1	R329^{ICL3}	E322	19.3			
K330^{ICL3}	E322	27.0							R329^{ICL3}	E322	19.0						
R329^{ICL3}	D323	38.8							R329^{ICL3}	D323	32.0	R329^{ICL3}	D323	24.4			
R168^{ICL1}	F335	35.1															
K169^{ICL1}	F335	33.4															
									D327^{ICL3}	R342	12.5	D327^{ICL3}	R342	12.2	D339^{ICL3}	R342	13.9
R329^{ICL3}	D343	26.0	R329^{ICL3}	D343	42.0				R329^{ICL3}	D343	25.5	R329^{ICL3}	D343	41.8			
												K330^{ICL3}	D343	21.3			
						D339^{ICL3}	L346	31.2									
						M340^{ICL3}	L346	28.8									
			K330^{ICL3}	T350	27.8	D339^{ICL3}	T350	47.3									
						N343$^{6.30}$	T350	36.2									
						G342^{ICL3}	T350	29.1									
												R338$^{6.37}$	D354	9.7			
												E394$^{7.63}$	R356	6.4	E406$^{7.63}$	R356	10.7
						M340^{ICL3}	Y358	36.7									
						M340^{ICL3}	C359	37.2									
						M340^{ICL3}	Y360	43.6									
F248^{ICL2}	F376	51.6	F248^{ICL2}	F376	37.1	F259^{ICL2}	F376	81.7									
F248^{ICL2}	R380	46.4	S247^{ICL2}	R380	57.6	T258^{ICL2}	R380	69.3	S247^{ICL2}	R380	21.5	S247^{ICL2}	R380	12.8	T258^{ICL2}	R380	28.0
S247^{ICL2}	R380	40.3	F248^{ICL2}	R380	29.3	F259^{ICL2}	R380	55.9									
A245$^{3.59}$	R380	34.3	L345$^{6.44}$	R380	28.9	V256$^{3.59}$	R380	27.7									
V246$^{3.60}$	R380	25.6	A245$^{3.59}$	R380	28.2												
K322$^{5.64}$	D381	99.1	K322$^{5.64}$	D381	100.0	K334$^{5.64}$	D381	100.0	K322$^{5.64}$	D381	98.8	K322$^{5.64}$	D381	99.8	K334$^{5.64}$	D381	99.9
F248^{ICL2}	I383	37.8	S247^{ICL2}	I383	27.2	T258^{ICL2}	I383	75.1									
S247^{ICL2}	I383	29.9	F248^{ICL2}	I383	25.1	F259^{ICL2}	I383	41.8									

Supplementary Table 10 continued

L244$^{3.58}$	Q384	92.8	L244$^{3.58}$	Q384	93.7	K334$^{5.64}$	Q384	82.3	K322$^{5.64}$	Q384	46.4	K322$^{5.64}$	Q384	67.3	K334$^{5.64}$	Q384	61.1
K322$^{5.64}$	Q384	79.3	K322$^{5.64}$	Q384	89.8	L255$^{3.58}$	Q384	76.5	S247^{ICL2}	Q384	32.3	S247^{ICL2}	Q384	31.4	T258^{ICL2}	Q384	23.9
S247^{ICL2}	Q384	48.9	S247^{ICL2}	Q384	42.4	T258^{ICL2}	Q384	54.6									
L243$^{3.57}$	Q384	30.0															
K322$^{5.64}$	R385	60.0	K322$^{5.64}$	R385	49.6	K334$^{5.64}$	R385	77.5									
						M340^{ICL3}	R385	26.7									
L243$^{3.57}$	H387	84.4	L243$^{3.57}$	H387	78.8	L254$^{3.57}$	H387	75.1	S247^{ICL2}	H387	6.9						
			L244$^{3.58}$	H387	27.4	L255$^{3.58}$	H387	38.2									
						T258^{ICL2}	H387	27.2									
L244$^{3.58}$	L388	51.7	L244$^{3.58}$	L388	52.5	K334$^{5.64}$	L388	41.5									
K322$^{5.64}$	L388	35.1	K322$^{5.64}$	L388	48.0	L255$^{3.58}$	L388	38.3									
L319$^{5.61}$	L388	31.1	L319$^{5.61}$	L388	29.4												
															E406$^{7.63}$	R389	15.7
R174$^{2.46}$	Q390	30.0				N404$^{7.61}$	Q390	36.8	E394$^{7.63}$	Q390	10.4	E394$^{7.63}$	Q390	5.8	E406$^{7.63}$	Q390	11.4
L240$^{3.54}$	Y391	47.7	R174$^{2.46}$	Y391	59.7	R185$^{2.46}$	Y391	68.6	E236$^{3.50}$	Y391	23.1	R174$^{2.46}$	Y391	30.4	R185$^{2.46}$	Y391	7.4
Y388$^{7.57}$	Y391	42.3	L240$^{3.54}$	Y391	56.5	L251$^{3.54}$	Y391	62.4	H178$^{2.50}$	Y391	19.1						
H178$^{2.50}$	Y391	27.3	L243$^{3.57}$	Y391	34.1	L254$^{3.57}$	Y391	35.9	Y388$^{7.57}$	Y391	13.5						
			Y239$^{3.53}$	Y391	26.4	Y250$^{3.53}$	Y391	26.9									
						L357$^{6.44}$	Y391	26.4									
R338$^{6.37}$	E392	68.7	L345$^{6.44}$	E392	34.5	L357$^{6.44}$	E392	46.5	R338$^{6.37}$	E392	46.8	R338$^{6.37}$	E392	10.4	R353$^{6.40}$	E392	7.5
R341$^{6.40}$	E392	27.5				G405$^{7.62}$	E392	34.1	R341$^{6.40}$	E392	12.9						
S342$^{6.41}$	L393	89.1	S342$^{6.41}$	L393	88.4	S354$^{6.41}$	L393	87.3									
L319$^{5.61}$	L393	71.3	L319$^{5.61}$	L393	72.1	L331$^{5.61}$	L393	63.8									
L346$^{6.45}$	L393	33.9	L346$^{6.45}$	L393	44.2	L358$^{6.45}$	L393	53.0									
			L345$^{6.44}$	L393	26.8	L357$^{6.44}$	L393	30.4									
R338$^{6.37}$	L394	99.9	R338$^{6.37}$	L394	65.1	R350$^{6.37}$	L394	77.4	R338$^{6.37}$	L394	99.9	R338$^{6.37}$	L394	64.2	R350$^{6.37}$	L394	76.2
L323$^{5.65}$	L394	51.6	L323$^{5.65}$	L394	51.6	K334$^{5.64}$	L394	40.3				R341$^{6.40}$	L394	13.1	R353$^{6.40}$	L394	9.8
K322$^{5.64}$	L394	41.1	K322$^{5.64}$	L394	27.0	L335$^{5.65}$	L394	37.2									
L319$^{5.61}$	L394	25.8	L319$^{5.61}$	L394	26.2	L331$^{5.61}$	L394	28.2									
**VPAC1R:VIP:Gβ**			**VPAC1R:PACAP27:Gβ**			**PAC1R:PACAP27:Gβ**			**VPAC1R:VIP:Gβ**			**VPAC1R:PACAP27:Gβ**			**PAC1R:PACAP27:Gβ**		
															R413$^{7.70}$	D291	15.1
															R416$^{7.73}$	D291	13.4
															S417$^{7.74}$	N293	7.2
R401$^{7.70}$	D312	88.3	R401$^{7.70}$	D312	47.6	R179^{ICL1}	D312	56.5	R401$^{7.70}$	D312	75.8	K169^{ICL1}	D312	45.6	R179^{ICL1}	D312	53.2
K169^{ICL1}	D312	51.5	K169^{ICL1}	D312	46.8				K169^{ICL1}	D312	43.9	R401$^{7.70}$	D312	34.4	K414$^{7.71}$	D312	20.5
									R168^{ICL1}	D312	16.1	R404$^{7.73}$	D312	24.5	R413$^{7.70}$	D312	15.4
									K402$^{7.71}$	D312	15.6	R405$^{7.74}$	D312	16.9	K180^{ICL1}	D312	7.3
									R405$^{7.74}$	D312	8.2	K402$^{7.71}$	D312	7.2			
K169^{ICL1}	D333	60.0							K169^{ICL1}	D333	59.1				R179^{ICL1}	D333	8.2
R168^{ICL1}	D333	27.8							R168^{ICL1}	D333	27.4						

Source Data for Supplementary Information file: Uncropped Coomassie-stained SDS-PAGE of the complex samples used for cryo-EM imaging (cropped gels shown in Supplementary Figure 1)

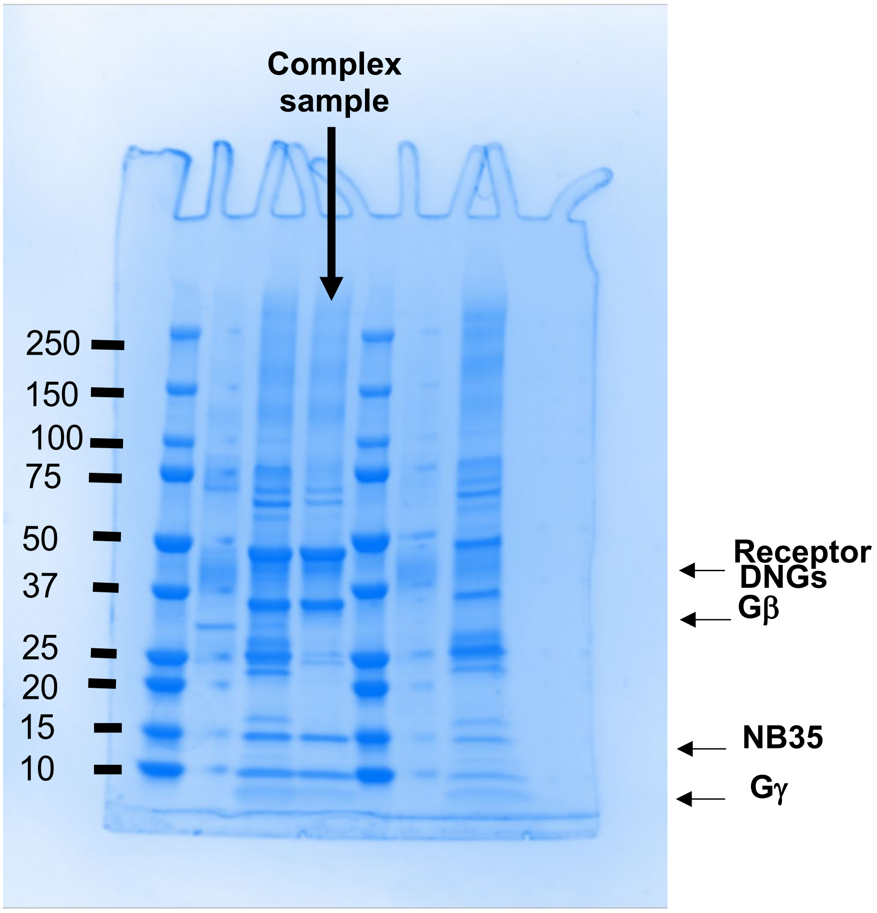

PAC1R-PACAP27

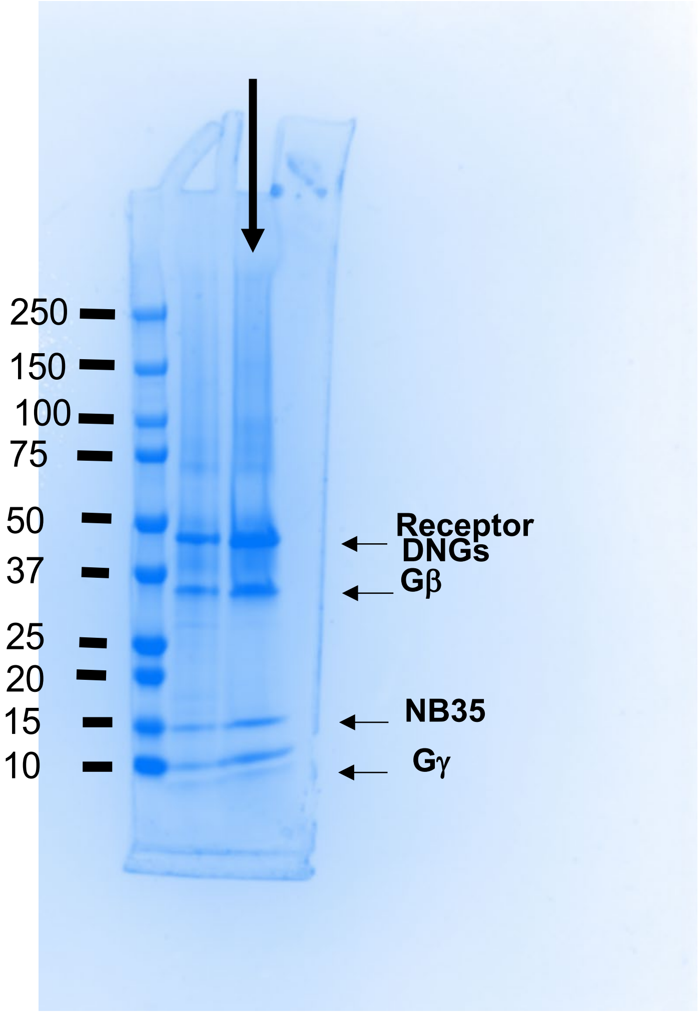

VPAC1R-PACAP27

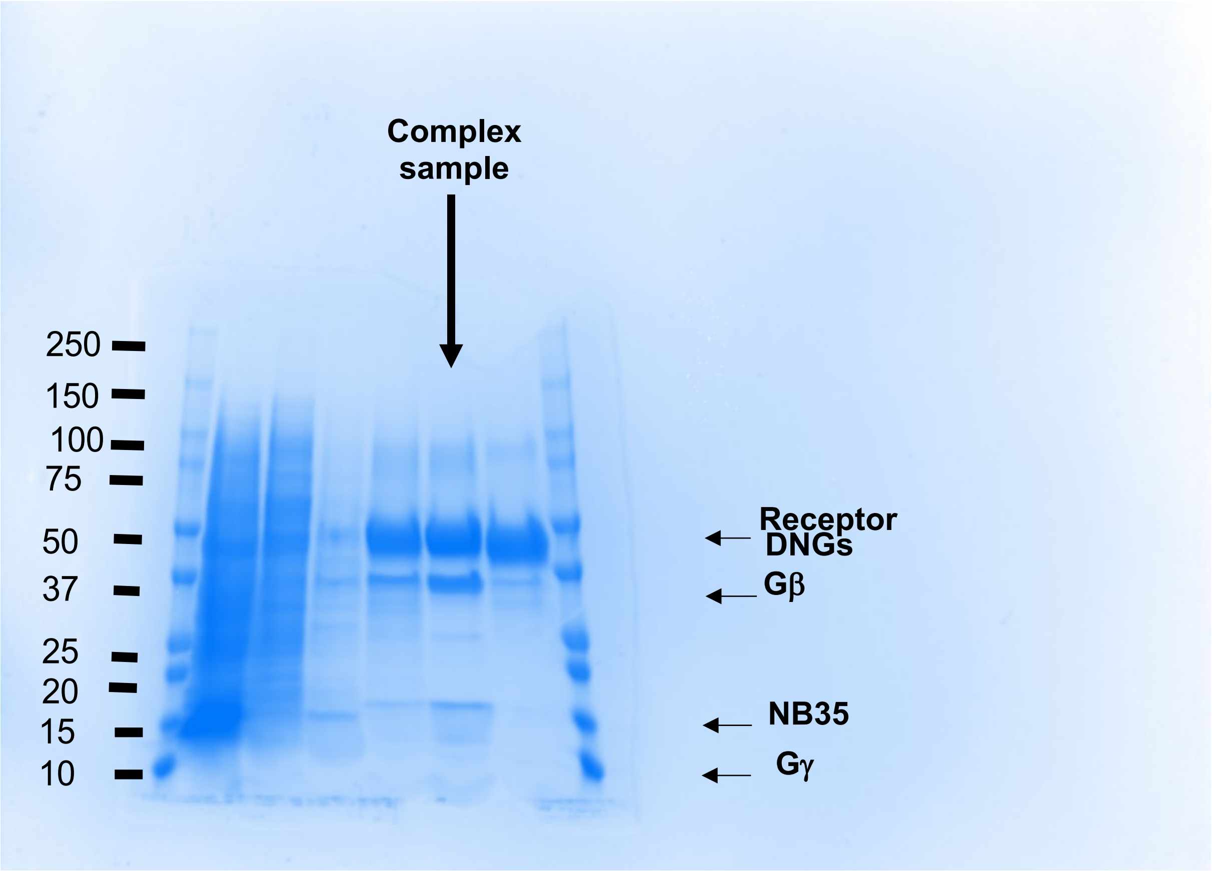

Supplement: Supplementary file 1 — Supplementary Information [file 41467_2022_34629_MOESM1_ESM.pdf]
