# Supplementary material for: Understanding VPAC receptor family peptide binding and selectivity
Source: Nat Commun. 2022 Nov 16;13:7013. doi: 10.1038/s41467-022-34629-3 (PMC9668914; doi:10.1038/s41467-022-34629-3)
Supplement: Supplementary file 3 — Description of Additional Supplementary Files [file 41467_2022_34629_MOESM3_ESM.pdf]

**File name: Supplementary Movie 1**

**Description:** CryoSPARC 3D variability analysis (3DVA) showing movements of the receptor-peptide-Gs complexes in their first, second and third principal components at different threshold levels and orientations (front and side views). Components 0, 1 and 2 are coloured in a colour gradient from dark to light. 3DVA are shown with PAC1R-PACAP27 first (pink), VPAC1R-PACAP27 second (blue) and VPAC1R-VIP third (green).

**File name: Supplementary Movie 2**

**Description:** CryoSPARC 3D variability analysis (3DVA) with model morphs between component 0 and 19 of the 3D variability analysis frames. Maps of components 0, 1 and 2 are coloured in grey transparent and models are shown as ribbons and coloured by chains (see Supplementary Figure 2A-2C). 3DVA maps and models are shown in order of PAC1R-PACAP27 (pink), VPAC1R-PACAP27 (blue) and VPAC1R-VIP (green), starting with frame 0 and then morphing to frame 19 and then back to frame 0. The last movie part shows a side-byside comparison of component 0 morphs of all three complexes.

**File name: Supplementary Movie 3**

**Description:** Example of bound-unbound (peptide unbinding; first half of the movie) and unbound-bound (peptide binding; second half of the movie) transitions from metadynamics and supervised molecular dynamics (simulations merge) of the PAC1-PACAP27, VPAC1R-VIP, VPAC1R-PACAP27 and PAC1R-VIP complex. Classic MD simulations were seeded from transition trajectories to increase the sampling along the unbinding and binding pathways. Peptides are coloured in orange; receptors are coloured in grey. ECL: Extracellular loop. ECD: Extracellular domain. TM: Transmembrane helix. NTD: Nterminal domain.
