# Supplementary material for: Understanding VPAC receptor family peptide binding and selectivity
Source: Nat Commun. 2022 Nov 16;13:7013. doi: 10.1038/s41467-022-34629-3 (PMC9668914; doi:10.1038/s41467-022-34629-3)
Supplement: Supplementary file 7 — Reporting Summary [file 41467_2022_34629_MOESM7_ESM.pdf]

nature portfolio

Corresponding author(s): Denise Wootten
Patrick Sexton

Last updated by author(s): 18th Oct 2022

Reporting Summary

Nature Portfolio wishes to improve the reproducibility of the work that we publish. This form provides structure for consistency and transparency in reporting. For further information on Nature Portfolio policies, see our Editorial Policies and the Editorial Policy Checklist.

Statistics

For all statistical analyses, confirm that the following items are present in the figure legend, table legend, main text, or Methods section.

n/a	Confirmed	
☐	☒	The exact sample size (*n*) for each experimental group/condition, given as a discrete number and unit of measurement
☐	☒	A statement on whether measurements were taken from distinct samples or whether the same sample was measured repeatedly
☐	☒	The statistical test(s) used AND whether they are one- or two-sided<br>*Only common tests should be described solely by name; describe more complex techniques in the Methods section.*
☒	☐	A description of all covariates tested
☒	☐	A description of any assumptions or corrections, such as tests of normality and adjustment for multiple comparisons
☐	☒	A full description of the statistical parameters including central tendency (e.g. means) or other basic estimates (e.g. regression coefficient) AND variation (e.g. standard deviation) or associated estimates of uncertainty (e.g. confidence intervals)
☐	☒	For null hypothesis testing, the test statistic (e.g. F, t, r) with confidence intervals, effect sizes, degrees of freedom and P value noted<br>*Give P values as exact values whenever suitable.*
☒	☐	For Bayesian analysis, information on the choice of priors and Markov chain Monte Carlo settings
☒	☐	For hierarchical and complex designs, identification of the appropriate level for tests and full reporting of outcomes
☒	☐	Estimates of effect sizes (e.g. Cohen's *d*, Pearson's *r*), indicating how they were calculated

Our web collection on statistics for biologists contains articles on many of the points above.

Software and code

Policy information about availability of computer code

Data collection	Data collection for cryo-EM data was conducted using SerialEM 3.7. BMG Pherastar platereader and MARS analysis software v3
Data analysis	Cryo-EM data analysis/visualisation softwares:<br>UCSF Chimera-v1.14/ChimeraX-v1.2.5, Relion v3.1, CryoSPARC-v2, cryOLO 1.5, MotionCor2, GCTF, EMAN2.2, Coot 0.9, Phenix 1.19, Molprobity v4, Isolde 1.1, SwissModel, LigPlotPlus v2.2<br><br>Molecular dynamics softwares:<br>PDB2PQR, PROPKA, VMD Solvate plugin 1.5, VMD autoionize plugin 1.3, ACEMD, GetContacts , SuMD<br><br>Pharmacology and mutagenesis data analysis software:<br>GraphPadPrism 9.0, FloJo 10.6.1

For manuscripts utilizing custom algorithms or software that are central to the research but not yet described in published literature, software must be made available to editors and reviewers. We strongly encourage code deposition in a community repository (e.g. GitHub). See the Nature Portfolio guidelines for submitting code & software for further information.

Data

Policy information about availability of data

All manuscripts must include a data availability statement. This statement should provide the following information, where applicable:

- Accession codes, unique identifiers, or web links for publicly available datasets
- A description of any restrictions on data availability
- For clinical datasets or third party data, please ensure that the statement adheres to our policy

CryoEM maps and atomic model coordinate files for complexes determined in this study are available on the EMDB and PDB: VPAC1R-VIP complex: PDB 8E3Z / EMDB 27874; VPAC1R-PACAP27 complex: PDB 8E3Y / EMDB 27873; PAC1R-PACAP27 complex: PDB 8E3X / EMDB 27872.
Atomic coordinates for structures not determined in this study but used for comparison or as a starting model for the PAC1R atomic coordinates are available from the PDB: 6X18 (GLP1R-GLP1), 6X1A (GLP1R-PF-06882961), 6P9Y (PAC1R-PACAP38), 6M1I (PAC1R-PACAP38), 6LPB (PAC1R-PACAP38), 6VN7 (VPAC1R-PACAP27).

Raw data for the pharmacology experiments are provided as source data

Field-specific reporting

Please select the one below that is the best fit for your research. If you are not sure, read the appropriate sections before making your selection.

☒ Life sciences ☐ Behavioural & social sciences ☐ Ecological, evolutionary & environmental sciences

For a reference copy of the document with all sections, see nature.com/documents/nr-reporting-summary-flat.pdf

Life sciences study design

All studies must disclose on these points even when the disclosure is negative.

Sample size	Each GPCR complex was prepared once for structural studies, and one cryo-EM dataset comprising several thousand micrographs was collected. The number of micrographs in each dataset was determined based on the available microscope time and our experience with GPCR complexes. No statistical methods were used to predetermine sample size. All functional data were obtained from at least three independent experiments to ensure each data point was repeatable. For MD four replicas were performed. The decision of sample size for MD was made based in the authors experience of related systems (ie Deganutti et al., 2022, Nature Commun 13 (1), 1-18, Cuzzolin et al., 2016 JCIM 56 (4) 687-705, Cary et al., Nat Chem Biol 18(3) 256-263) to obtain adequate comformational sampling for the purpose of this study.
Data exclusions	In accordance with standard cryo-EM practice, micrographs with low estimated CTF resolution were excluded from further processing. No data were excluded from the pharmacological assays.
Replication	No replication was performed for structural studies in complex preparation or data acquisition. Each cryo-EM dataset comprises millions of copies of the investigated GPCR complex and therefore has inherent replication by using random particle subsets during the analyses. For pharamcological assays, each individual experiment was performed in duplicate or triplicate. All findings were reliably reproduced.
Randomization	Cryo-EM - Random particle subsets were used during the 3D auto-refinement steps in Relion. Single particle cryo-EM data processing follows gold standard, that is to separate particle stacks by the order, even or odd, into two separate data sets, and processed separately. This separation is considered random. All biochemical experiments were initiated from independent aliquots of the frozen cells expressing the target proteins, and then subjected to indicated experimental conditions. While randomisation was not performmed for pharmacology experiments, plates layouts were altered for each independent experiment such that the location of different cell lines or drug dilutions differed in the different experimental repeats. No animals or human research participants are involved in this study.
Blinding	Blinding was not performed in this study, as protein samples are not required to be allocated into experimental groups in protein structural studies, and no animals or human research participants are involved in this study.

Reporting for specific materials, systems and methods

We require information from authors about some types of materials, experimental systems and methods used in many studies. Here, indicate whether each material, system or method listed is relevant to your study. If you are not sure if a list item applies to your research, read the appropriate section before selecting a response.

Materials & experimental systems

n/a	Involved in the study
☐	☒ Antibodies
☐	☒ Eukaryotic cell lines
☒	☐ Palaeontology and archaeology
☒	☐ Animals and other organisms
☒	☐ Human research participants
☒	☐ Clinical data
☒	☐ Dual use research of concern

Methods

n/a	Involved in the study
☒	☐ ChIP-seq
☐	☒ Flow cytometry
☒	☐ MRI-based neuroimaging

Antibodies

Antibodies used	Mouse anti-FLAG M2 antibody (MIPS TC13-03-01-03; stock concentration 10.15mg.ml - 1:5000 dilution used); Goat anti-mouse AF647 (Invitrogen #A21235 - stock concentration 2mg/ml - 1:2000 dilution used).
Validation	All antibodies were validated by the manufacturers. In our study these were used to detect FLAG tagged proteins recombinantly expressed in cells. Untransfected cells were used in our study as a control to detect any non-specific binding of the antibodies in these cells.

Eukaryotic cell lines

Policy information about cell lines

Cell line source(s)	Trichoplusia ni (Tni) insect cells (Expression systems); Chinese Hamster Ovary (CHO) cells (ATCC); COS-7 cells (ATTC)
Authentication	Grown from original stocks purchased from suppliers. Cell lines were not authenticated in house.
Mycoplasma contamination	Cells were tested regularly in our laboratory and were free of mycoplasma contamination
Commonly misidentified lines (See ICLAC register)	not relevant - No commonly misidentified cell lines were used.

Flow Cytometry

Plots

Confirm that:

☒ The axis labels state the marker and fluorochrome used (e.g. CD4-FITC).

☒ The axis scales are clearly visible. Include numbers along axes only for bottom left plot of group (a 'group' is an analysis of identical markers).

☒ All plots are contour plots with outliers or pseudocolor plots.

☒ A numerical value for number of cells or percentage (with statistics) is provided.

Methodology

Sample preparation	On the day of assay, cells were washed with PBS once and treated with Versene to detach cells from dish. Antibody staining as described in the methods section of this paper. S
Instrument	ample analysed on BD FACS Canto II flow cytometer.
Software	FlowJo v10.6.1
Cell population abundance	N/A – flow cytometry only used for analysis purposes not for cell sorting.
Gating strategy	Cells were first gated in FSC-A (x-axis) versus SSC-A (y-axis). Single cells identified in FSC-A (x-axis) versus FSC-H (y-axis), and gated for live cells (Sytox Blue negative cells) in FSC-A (x-axis) versus Sytox Blue (y-axis). Sytox Blue-negative was considered to be signal less than 913.

☒ Tick this box to confirm that a figure exemplifying the gating strategy is provided in the Supplementary Information.
